# Cardio-selective versus non-selective *β*-blockers for cardiovascular events and mortality in long-term dialysis patients: A systematic review and meta-analysis

**DOI:** 10.1371/journal.pone.0279171

**Published:** 2022-12-19

**Authors:** Shaohua Tao, Junlin Huang, Jie Xiao, Guibao Ke, Ping Fu

**Affiliations:** 1 Kidney Research Institute, Division of Nephrology, West China Hospital of Sichuan University, Chengdu, Sichuan, China; 2 Department of Cardiology, Guangdong Provincial People’s Hospital, Guangdong Academy of Medical Sciences, Guangzhou, Guangdong, China; 3 Department of Nephrology, The First Affiliated Hospital of Guangzhou Medical University, Guangzhou, Guangdong, China; Universidade Estadual Paulista Julio de Mesquita Filho, BRAZIL

## Abstract

**Background:**

Trials in patients receiving dialysis have demonstrated that *β*-blockers reduce all-cause mortality and cardiovascular events. However, differences still exist within-class comparative effectiveness studies of the therapeutic benefits of *β*-blockers in dialysis patients.

**Objective:**

The purpose of this systematic review is to examine whether cardiovascular events and all-cause mortality differed between dialysis patients receiving cardio-selective and non-selective agents.

**Methods:**

A comprehensive search of relevant articles from the PubMed, EMBASE, Cochrane Central Register and ClinicalTrials.gov was performed up to September 4, 2022, we included adults receiving *β*-blockers to evaluate the effects of cardio-selective versus non-selective agents on mortality and cardiovascular events in the dialysis population. Hazard ratios (HRs) and 95% confidence intervals (CIs) were examined for the negative outcomes of cardiovascular events and death for any reason. The risk of bias in randomized controlled trials (RCTs) was assessed using Cochrane’s risk of bias tool and the risk of bias in observational studies was assessed using a table designed according to the ROBINS-I tool, the evidence grade was assessed using the GRADE guideline. For all-cause mortality and cardiovascular events, the RevMan software (version 5.3) was used to calculate pooled HRs with 95% CI. The heterogeneity (*I*^*2*^) in statistics was used to examine the degree of heterogeneity among studies.

**Results:**

Four observational studies, including 58, 652 long-term dialysis patients, were included in the meta-analysis. Compared to dialysis patients who took non-selective *β*-blockers, who took cardio-selective *β*-blockers was probably associated with fewer cardiovascular events (hazard ratio [HR] = 0.85, 95% confidence interval [CI] = 0.81, 0.89, heterogeneity [*I*^*2*^] = 0%, three trials, 52,077 participants, *moderate-quality evidence*) and may have lower all-cause mortality (HR = 0.83, 95% CI = 0.69, 0.99, *I*^*2*^ = 91%, four trials, 54,115 participants, *low-quality evidence*).

**Conclusions:**

This systematic review showed that cardio-selective *β*-blockers are probably associated with fewer cardiovascular events and may have lower all-cause mortality in long-term dialysis patients than non-selective *β*-blockers. The present study results need to be replicated using randomized controlled trials with longer observation durations.

## Background

Cardiovascular disease is the major killer in long-term dialysis patients [1, 2], for several reasons. Among these reasons, uremic states weaken long-term dialysis patients’ immune systems, which could trigger cardiovascular events [3]. Another reason is that patients who undergo long-term intermittent hemodialysis are more likely to experience high variability in electrolytes, hemodynamics, and heart rate, each of which can result in cardiovascular events and mortality. Finally, approximately 80% of patients with end-stage renal disease (ESRD) who receive dialysis therapy have at least one cardiac disease and are at a higher risk of cardiovascular events. Compared to the general population, ESRD patients receiving long-term maintenance dialysis therapy are more likely to die from cardiovascular events (about 10%–15% per year in Europe and 20% in the United States) [4, 5]. However, even with recent developments in dialysis treatment, long-term dialysis patients still have cardiovascular mortality rates five to seven times higher than those in the general population [4], partly due to a lack of evidence-based drug therapy strategies to improve the outcome of cardiac diseases in dialysis patients.

It is known that cardioprotective medications, such as *β*-blockers, effectively reduce cardiovascular events and all-cause mortality [6–8]. According to their pharmacological targets, *β*-blockers have been subdivided into those with non-selective properties (*β*_1+2_ blockers or *α*_1_+*β*_1+2_ blockers such as carteolol, nadolol, penbutolol, pindolol, propranolol, sotalol, timolol, carvedilol, bucindolol, labetalol, arotinolol, and arotinolol) and those with cardio-selective properties (*β*_1_ blockers such as betaxolol, esmolol, celiprolol, acebutolol, atenolol, bisoprolol, metoprolol, and nebivolol) [9, 10]. However, existing data on the effects of cardio-selective versus non-selective agents on the incidence of cardiovascular events and all-cause mortality in dialysis patients are sparse, and there is a lack of well-powered evidence to help guide healthcare providers’ decisions about which specific *β*-blocker to prescribe to dialysis patients. Therefore, we conducted a systematic review of dialysis patients to clarify whether cardiovascular events and all-cause mortality varied between cardio-selective and non-selective agents.

## Methods

This systematic review and meta-analysis were reported with adherence to the Preferred Reporting Items for Systematic Reviews and Meta-Analyses (PRISMA) guidelines, an evidence-based minimum set of items for reporting in systematic reviews and meta-analyses. The research question was designed according to the Population/Intervention /Comparison/ Outcome(s) criteria (PICO):

**P**: CKD patients receiving long-term maintenance dialysis therapy (1. Confirmed CKD diagnosis; 2. Received long-term maintenance dialysis therapy; 3. Adult patients (males and females)).

**I**: Patients were intervened with *β* blockers.

**C**: Cardio-selective and non-selective *β*-blockers relative to match outcomes at the competitive level.

**O**: Measured outcomes in all studies included cardiovascular event incidence and all-cause mortality indices.

**Design**: Systematic review.

**Time filter**: From inception date to September 4, 2022.

**Language filter**: No restrictions.

Original investigations published in scholarly and peer-reviewed journals and unpublished data from studies designed as randomised controlled trial and observational study. All studies were included if they reported at least one of the all-cause mortality and cardiovascular events.

Publications comparing cardio-selective and non-selective *β*-blockers between CKD patients who did not receive long-term maintenance dialysis therapy were excluded. Publications without quantitative information and details, duplicate publications, reviews, case reports, editorials, abstracts, comments, animal experiments, and cell experiments were excluded.

### Data sources and search strategy

A thorough and comprehensive investigation was conducted on articles referenced in PubMed, EMBASE, Cochrane Central Register, and ClinicalTrials.gov from the inception date to September 4, 2022, without restrictions on the follow-up period. To evaluate the effects of cardio-selective (*β*_1_ blockers) versus non-selective agents (*β*_1+2_ blockers or *α*_1_+*β*_1+2_ blockers) on mortality and cardiovascular events in the dialysis population, we used free-text terms and MeSH terms in our search, which primarily included the following search terms: “Carvedilol,” “Bucindolol,” “Gencaro,” “Labetalol,” “Arotinolol,” “Almarl,” “Betaxolol,” “Esmolol,” “Celiprolol,” “Acebutolol,” “Atenolol,” “Bisoprolol,” “Metoprolol,” “Nebivolol,” “Carteolol,” “Nadolol,” “Penbutolol,” “Pindolol,” “Propranolol,” “Sotalol,” “Timolol,” “Cardio-selective *β*-blockers,” “Non-selective agents,” “Dialysis,” “Renal Replacement Therapy,” “Hemodialysis,” and “Hemodialyses”. Other relevant studies were identified by reviewing the reference lists and other systematic reviews (S1 File). Two reviewers (G. K. and J. H.) independently performed the studies selection process. Any study that did not meet the predetermined criteria was excluded. If there was any disagreement about the selection process, a third reviewer (J. X.) would assess them and discuss with other reviewers (G. K. and J. H.) to made a consensus decision about whether to include or exclude. An additional search of Google Scholar of relative studies was also included to ensure that any unpublished studies were identified for relevant use. The additional search was to avoid bias due to the selective inclusion of trial effect estimates. All retrieved studies were exported to EndNote to remove duplicates.

### Data extraction and study quality assessment

Two reviewers (G. K. and J. H.) obtained the following information from each of the included studies: the first author, year of publication, study center, study design, enrollment period, sample size, interventions, length of the follow-up period, the incidence of cardiovascular events, and death for any reason (all-cause mortality). Hazard ratios (HRs) and 95% confidence intervals (CIs) were examined for the negative outcomes of cardiovascular events and death for any reason. Data were extracted from survival curves when HRs were not available. Data extraction was performed using a standardized form.

Quality assessment was performed using a standardized form. The evidence grade was assessed using the Grading of Recommendations, Assessment, Development, and Evaluation (GRADE) guideline, and the certainty of each outcome was assessed as high, moderate, low, or very low through consideration of five judgment domains: imprecision, inconsistency, indirectness, publication bias, imprecision, inconsistency, indirectness, and publication bias [11, 12]. The risk of bias in randomized controlled trials (RCTs) was assessed using Cochrane’s risk of bias tool for assessing bias risk in randomized trials, and the risk of bias in observational studies was assessed using a table designed according to the principle of the Risk of Bias in Non-Randomized Studies–of Interventions (ROBINS-I) [13]. Assessment of publications quality, risk of bias and the evidence grade were carried out by two reviewers (G. K. and J. H.) independently. If there was any disagreement about the assessment, a third reviewer (J. X.) would reassess them and discuss with other reviewers (G. K. and J. H.) to made a consensus decision.

### Statistical analyses

Pooled HRs and 95% CIs were calculated to examine whether the use of cardio-selective or non-selective agents was associated with differences in all-cause mortality and cardiovascular events. The heterogeneity (*I*^*2*^) in statistics was used to examine the degree of heterogeneity among studies. A fixed-effects model was used for no significant heterogeneity (*I*^*2*^ < 50%); otherwise, a random-effects model was employed. If studies available for meta-analysis with at least 10 enrolled studies, funnel plots and statistical tests were used to identify possible publication bias; where there are fewer than 10 studies available for inclusion in a meta-analysis, we will describe the potential publication bias. When HRs were unavailable, data were collected from the survival curves using Engauge Digitizer software version 10.8 [14, 15]. RevMan software (version 5.3) was used to prepare and maintain reviews from different databases, and statistical analyses were performed using Stata 12.0. Statistical significance was defined as *P* <0.05.

## Results

### Search results

**Fig 1:**
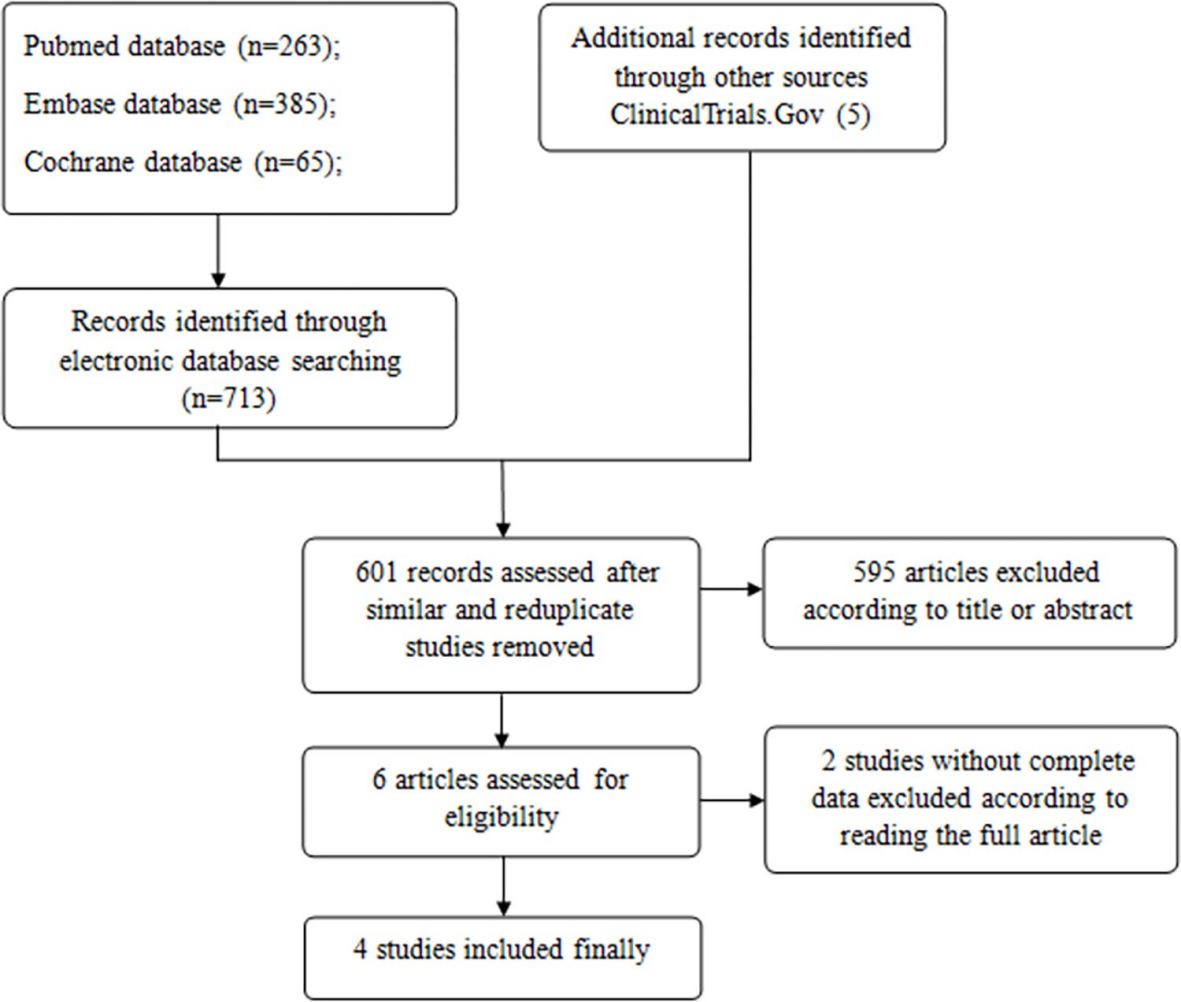
PRISMA flow diagram of study selection.

Initially, 713 primary literature were identified, and 5 were identified through the search of ClinicalTrials.gov. Next, 601 records were assessed after similar and duplicate studies were removed, of which 595 were removed after a careful review of all titles and abstracts in detail. The full texts of the 6 remaining studies were then scrutinized, and as a result, 2 studies with little necessary data regarding our study were eliminated (S2 File). No unpublished works were available since no unpublished results met the inclusion criteria. Finally, 4 observational studies with a total of 58,652 participants met all the inclusion criteria and those 4 studies were included in the meta-analysis (Fig 1) [6, 7, 16, 17] (S3 File). The PRISMA checklist is listed in S4 File.

### Study characteristics

The descriptive statistics for the participants in each of the 4 studies are presented in Table 1. Patients in the non-selective *β*-blockers group received treatment with a *β*_1+2_ or *α*_1_+*β*_1+2_ blocker, while patients in the cardio-selective *β*-blockers group received *β*_1_ blockers.

**Table 1:** General characteristics of all included studies.

Study	Study Design	Journal	Enrollment Period (year)	Age Cardio-selective / non-selective *β*-blockers	N Cardio-selective / non-selective *β*-blockers	Interventions Cardio-selective / non-selective *β*-blockers		Follow-up	Main outcomes
Wu ^et al^, 2021	Observational study	Clin Kidney J	2004–2011	56.3±13.0/57.1± 13.1	9305/11171	1)high-dose bisoprolol (≥10 mg/day) or low-dose bisoprolol (1.25–<10 mg/day); 2) Dialyzed regularly (detail not mentioned; patients underwent chronic maintenance HD for >90 days).	1)high-dose carvedilol (≥50 mg/day) and low dose carvedilol (6.25–<50 mg/day); 2) Dialyzed regularly (detail not mentioned; patients underwent chronic maintenance HD for >90 days).	2 years	All-cause mortality Cardiovascular events
Assimon ^et al^, 2018	Observational study	Am J Kidney Dis	2007–2012	59.5±14.9/59.8±14.4	17506/9558	1)Received metoprolol (detail not mentioned); 2) α-Blocker; ACEI; CCB; Angiotensin receptor blocker; Diuretic; Vasodilatorl; Statin; cholesterol medication; Digoxin; nitrate; Antiplatelet medication; Anticoagulant medication; Midodrine; inhibitor of CYP2D6; 3) Dialyzed regularly (detail not mentioned; patients received peritoneal dialysis or home hemodialysis during the baseline period were excluded).	1) Received carvedilol (detail not mentioned); 2) α-Blocker; ACEI; CCB; Angiotensin receptor blocker; Diuretic; Vasodilatorl; Statin; cholesterol medication; Digoxin; nitrate; Antiplatelet medication; Anticoagulant medication; Midodrine; inhibitor of CYP2D6; 3) Dialyzed regularly (detail not mentioned; patients received peritoneal dialysis or home hemodialysis during the baseline period were excluded).	1 year	All-cause mortality Cardiovascular events
Shireman ^et al^, 2016	Observational study	BMC Cardiovasc Disord	2000–2005	60.4±15.1/58.3±15.9 or 60.1±15.2/57.6±16.0	7276/2199	1) Received atenolol or metoprolol (detail not mentioned); 2) CCB, ACEI; 3) Dialyzed regularly (detail not mentioned; home hemodialysis or peritoneal dialysis included).	1) Received carvedilol or labetalol (detail not mentioned); 2) CCB, ACEI; 3) Dialyzed regularly (detail not mentioned; home hemodialysis or peritoneal dialysis included).	2000 days	All-cause mortality Cardiovascular events
Tang ^et al^, 2016	Observational study	J Am Heart Assoc	1999–2010	67.3±11.1	629/1008	1) Received 4.4 mg bisoprolol a day (detail not mentioned); 2) Dialyzed regularly (patients on peritoneal dialysis were excluded; detail not mentioned).	1) Received 16.4mg carvedilol a day (detail not mentioned); 2) Dialyzed regularly (patients on peritoneal dialysis were excluded; detail not mentioned).	60 months	All-cause mortality

Data shown as mean [SD]. N: Number; CCB: Calcium channel blocker; ACEI: Angiotensin-converting enzyme inhibitor.

### GRADE evidence profile

By following the GRADE guideline, the quality of the evidence profile is shown in the S5 File, and the ROBINS-I-based bias risk assessment table of observational studies is shown in S6 File. According to ROBINS-I assessment in the item of bias due to confounding, such potential confounder was not further analyzed as a subgroup, or adjusted in the original data analysis 3 studies (serious ROBINS-1) [7, 16, 17], only one study by Tang *et al* [6] reported chronic obstructive pulmonary disease (COPD) as a factor in baseline characteristic comparation). According to the GRADE evaluation of two outcomes, the quality of the evidence of all-cause mortality if low due to lower certainty caused by the wide the 95% CI range of pooled HR in the item “imprecision”, and the lower certainty caused by ROBINS-I assessment in the item “risk of bias”, consequently, low confidence is placed in the estimates obtained from pooling studies in meta-analysis; the quality of the evidence of cardiovascular event is moderate due to the lower certainty caused by ROBINS-I assessment in the item “risk of bias”.

### All-cause mortality

**Fig 2:**
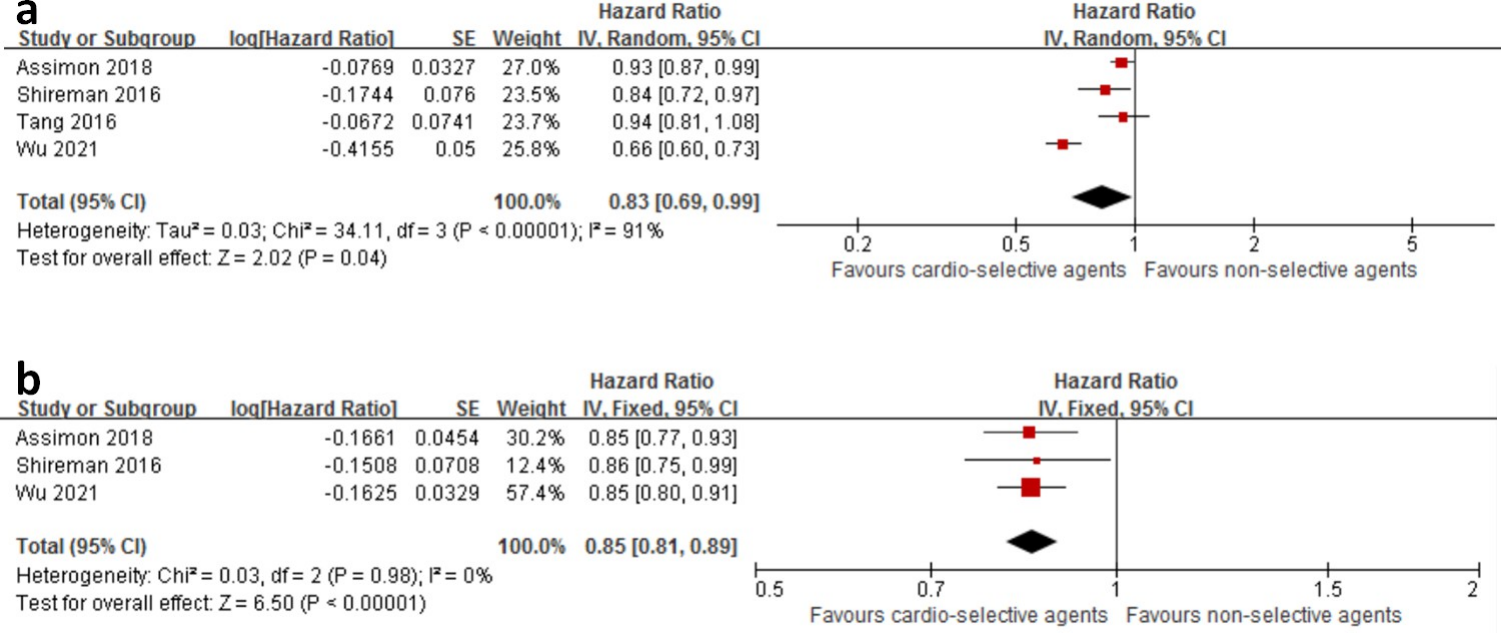
Forest plots for all-cause mortality and cardiovascular events. Cardio-selective *β*-blockers is associated with lower all-cause mortality (a) and cardiovascular events (b) in long-term dialysis patients when compared with non-selective *β*-blockers.

Pooling the data from the 4 studies in which all-cause mortality was assessed in 54,115 patients showed in a random-effects model that receiving treatment with cardio-selective *β*-blockers may have lower all-cause mortality (HR = 0.83, 95% CI = 0.69, 0.99, *I*^*2*^ = 91%, four trials, 54,115 participants, *low-quality evidence*) (Fig 2A). Significant heterogeneity was observed between the studies (*p* < 0.05, *I*^*2*^ = 91%).

### Cardiovascular events

Pooling the data from the 3 studies [7, 16, 17] that assessed the occurrence of cardiovascular events in 52,077 patients showed that compared with patients who received treatment with non-selective *β*-blockers, those who received treatment with cardio-selective *β*-blockers probably associated with fewer cardiovascular events (HR = 0.85, 95% CI = 0.81, 0.89, *I*^*2*^ = 0%, three trials, 52,077 participants, *moderate-quality evidence*, in the fixed effects model, Fig 2B). There was no heterogeneity among studies (*p* > 0.05, *I*^*2*^ = 0%).

### Public bias

There were too few studies in any one comparison to be able to adequately evaluate the risk of publication bias, we described the potential risk of each studies. Reporting and nonreporting bias were assessed to be low risk in all 4 studies. As all 4 studies performed from relatively large dataset, small-study effects bias were also assessed to be low risk.

## Discussion

To the best of our knowledge, this systematic review is the first to focus on patients undergoing long-term dialysis who were treated with either selective or non-selective *β*-blockers. Our analysis included 4 observational studies that included 58,652 long-term dialysis patients. Our results showed that participants who received cardio-selective *β*-blockers probably associated with fewer cardiovascular events and may have lower all-cause mortality than among those who received non-selective *β*-blockers. These findings could help shape the future role of cardio-selective *β*-blockers in treating dialysis patients.

Cardiovascular disease is the major killer in patients undergoing long-term dialysis [1, 2], with approximately 80% of long-term dialysis patients having one or more heart diseases [18]. Furthermore, it is likely that patients who undergo long-term intermittent hemodialysis are at an increased risk of cardiovascular events and experience higher all-cause mortality rates because of their exposure to high variability in heart rate, electrolytes, and hemodynamics [19]. Long-term dialysis patients are at an increased risk of cardiovascular events due to weakened or compromised immune systems [3]. Research has demonstrated that treatment with *β*-blockers can improve cardiovascular outcomes in patients undergoing dialysis [7, 8, 20]. However, whether cardio-selective and non-selective agents have differential effects on the incidence of cardiovascular events and all-cause mortality in patients receiving dialysis has not been studied and reported before the current study. Our meta-analysis helps fill this knowledge gap.

It is universally recognized that *β*-blockers have heterogeneous pharmacological and pharmacokinetic properties. The degree to which cardiac outcomes are affected by cardio-selective *β*-blockers (*β*1 selectivity) remains unknown. Both non-selective and cardio-selective *β*-blockers have been shown to reduce cardiovascular events, all-cause mortality, and hospitalizations [2, 21–23], Our results suggest that cardio-selective *β*-blockers may benefit long-term dialysis patients more. Why might this be true? One reason is that cardio-selective *β*-blockers have greater *β*1 selectivity than non-selective *β*-blockers and lower blood pressure by reducing cardiac output without affecting vascular resistance [16]. Second, cardio-selective *β*-blockers increase *β*1 receptor sensitivity to adrenergic stimulation and upregulate *β*1 receptor density [24]. In addition, since *β*2 receptors are involved in potassium influx into cells, *β*2 receptor antagonists could increase the possibility of hyperkalemia [25]. Finally, during ultrafiltration, *α*-receptor antagonists may reduce peripheral vasoconstriction mediated by compensatory sympathetic nervous system activity, thereby increasing the risk of hemodynamic instability during dialysis [7].

This systematic review had several limitations. First, this systematic review was not registered in PROSPERO; we tried to register our review in PROSPERO in 2020, but it was difficult at that moment because of the COVID-19 pandemic, and thus, we have performed data extraction without PROSPERO registration, according to the guidance notes for registering a systematic review protocol from the National Institute for Health Research (NIHR) in the UK, this systematic review was unable to register in PROSPERO as we have already initiated literature searches. Second, we could not examine the effects of dosage and duration of *β*-blocker use on the negative outcomes. Third, although the sample size in this meta-analysis was sufficiently large, we identified only 4 studies with relevant data that could be extracted. Therefore, our results require further validation. Fourth, the studies in this meta-analysis were non-randomized; and the limited quality of included studies according to the ROBINS-I risk assessment also limited the strength of this review, COPD and asthma as potential confounders were not further analyzed as a subgroup, or adjusted in the original data analysis 3 studies (serious ROBINS-1) [7, 16, 17], only one study by Tang *et al* [6] COPD as a factor in baseline characteristic comparation). Finally, our results indicate that the risk of publication bias in observational studies may be more significant than that in RCTs [26, 27]. In addition, according to the GRADE guidelines, the quality of the evidence is low.

In summary, the systematic review with low-quality evidence in the meta-analysis suggests that cardio-selective *β*-blockers were probably more effective at lowering the risk of cardiovascular events and may have lower all-cause mortality than non-selective agents in treating dialysis patients. However, 4 of the included observational studies had methodological limitations regarding study design. The certainty of the evidence was low according to the GRADE guideline-based assessment, mainly because of the risk of bias and inconsistency. Additional randomized controlled trials with larger sample sizes and a wider range of patient-important outcomes (e.g. description of the hemodialysis modality) and detailed information on the dosages of the drugs used are required.

## Conclusions

This systematic review with low-quality evidence in the meta-analysis showed that treating dialysis patients with cardio-selective *β*-blockers was probably associated with fewer cardiovascular events and may have lower all-cause mortality than with non-selective agents; higher-quality evidence is needed to replicate and confirm the validity of our findings.

## Supporting information

S1 File Search strategy. (DOCX)

S2 File Excluded studies with reasons. (XLSX)

S3 File Text files of four included studies. (PDF)

S4 File PRISMA 2020 checklist. (DOCX)

S5 File GRADE evidence profile. (DOCX)

S6 File Risk of bias assessment for observational studies (ROBINS-I). (XLSX)

S7 File Original data. (XLSX)

## References

[1] Foley RN, Parfrey PS, Sarnak MJ. Clinical epidemiology of cardiovascular disease in chronic renal disease. Am J Kidney Dis. 1998;32(5 Suppl 3):S112–9. doi: 10.1053/ajkd.1998.v32.pm9820470 9820470

[2] Tan Z, Ke G, Huang J, Yang D, Pi M, Li L, et al. Effects of Carvedilol on Cardiovascular Events and Mortality in Hemodialysis Patients, A Systematic Review and Meta-Analysis. Iran J Kidney Dis. 2020;14(4):256–66. 32655020

[3] Betjes MG. Immune cell dysfunction and inflammation in end-stage renal disease. Nat Rev Nephrol. 2013;9(5):255–65. doi: 10.1038/nrneph.2013.44 23507826

[4] Saran R, Robinson B, Abbott KC, Agodoa LY, Albertus P, Ayanian J, et al. US Renal Data System 2016 Annual Data Report: Epidemiology of Kidney Disease in the United States. Am J Kidney Dis. 2017;69(3 Suppl 1):A7–8. doi: 10.1053/j.ajkd.2016.12.004 28236831 PMC6605045

[5] Zazzeroni L, Pasquinelli G, Nanni E, Cremonini V, Rubbi I. Comparison of Quality of Life in Patients Undergoing Hemodialysis and Peritoneal Dialysis: a Systematic Review and Meta-Analysis. Kidney Blood Press Res. 2017;42(4):717–27. doi: 10.1159/000484115 29049991

[6] Tang CH, Wang CC, Chen TH, Hong CY, Sue YM. Prognostic Benefits of Carvedilol, Bisoprolol, and Metoprolol Controlled Release/Extended Release in Hemodialysis Patients with Heart Failure: A 10-Year Cohort. J Am Heart Assoc. 2016;5(1): e002584. doi: 10.1161/JAHA.115.002584 26738790 PMC4859376

[7] Assimon MM, Brookhart MA, Fine JP, Heiss G, Layton JB, Flythe JE. A Comparative Study of Carvedilol Versus Metoprolol Initiation and 1-Year Mortality Among Individuals Receiving Maintenance Hemodialysis. Am J Kidney Dis. 2018;72(3):337–48. doi: 10.1053/j.ajkd.2018.02.350 29653770 PMC6477681

[8] Jin J, Guo X, Yu Q. Effects of Beta-Blockers on Cardiovascular Events and Mortality in Dialysis Patients: A Systematic Review and Meta-Analysis. Blood Purif. 2019;48(1):51–9. doi: 10.1159/000496083 31039562

[9] Furgeson SB, Chonchol M. Beta-blockade in chronic dialysis patients. Semin Dial. 2008;21(1):43–8. doi: 10.1111/j.1525-139X.2007.00367.x 18251957

[10] Aoun M, Tabbah R. Beta-blockers use from the general to the hemodialysis population. Nephrol Ther. 2019;15(2):71–6. doi: 10.1016/j.nephro.2018.10.003 30718084

[11] Higgins JP, Thomas J, Chandler J, Cumpston M, Li T, Page MJ, et al. Cochrane Handbook for Systematic Reviews of Interventions version 6.3 (updated February 2022). Cochrane. 2022. Available from www.training.cochrane.org/handbook

[12] Brignardello-Petersen R, Mustafa RA, Siemieniuk RAC, Murad MH, Agoritsas T, Izcovich A, et al. GRADE approach to rate the certainty from a network meta-analysis: addressing incoherence. J Clin Epidemiol. 2019;108:77–85. doi: 10.1016/j.jclinepi.2018.11.025 30529648

[13] Sterne JA, Hernán MA, Reeves BC, Savović J, Berkman ND, Viswanathan M, et al. ROBINS-I: a tool for assessing risk of bias in non-randomised studies of interventions. BMJ. 2016;355:i4919. doi: 10.1136/bmj.i4919 27733354 PMC5062054

[14] Parmar MK, Torri V, Stewart L. Extracting summary statistics to perform meta-analyses of the published literature for survival endpoints. Stat Med. 1998;17(24):2815–34. 10.1002/(sici)1097-0258(19981230)17:24<2815::aid-sim110>3.0.co;2-8 9921604

[15] Tierney JF, Stewart LA, Ghersi D, Burdett S, Sydes MR. Practical methods for incorporating summary time-to-event data into meta-analysis. Trials. 2007;8:16. doi: 10.1186/1745-6215-8-16 17555582 PMC1920534

[16] Shireman TI, Mahnken JD, Phadnis MA, Ellerbeck EF. Effectiveness comparison of cardio-selective to non-selective β-blockers and their association with mortality and morbidity in end-stage renal disease: a retrospective cohort study. BMC Cardiovasc Disord. 2016;16:60. doi: 10.1186/s12872-016-0233-3 27012911 PMC4807583

[17] Wu PH, Lin YT, Liu JS, Tsai YC, Kuo MC, Chiu YW, et al. Comparative effectiveness of bisoprolol and carvedilol among patients receiving maintenance hemodialysis. Clin Kidney J. 2021;14(3):983–90. doi: 10.1093/ckj/sfaa248 33779636 PMC7986334

[18] Cheung AK, Sarnak MJ, Yan G, Berkoben M, Heyka R, Kaufman A, et al. Cardiac diseases in maintenance hemodialysis patients: results of the HEMO Study. Kidney Int. 2004;65(6):2380–9. doi: 10.1111/j.1523-1755.2004.00657.x 15149351

[19] Ku E, McCulloch CE, Ahearn P, Grimes BA, Mitsnefes MM. Trends in Cardiovascular Mortality Among a Cohort of Children and Young Adults Starting Dialysis in 1995 to 2015. JAMA Netw Open. 2020;3(9):e2016197. doi: 10.1001/jamanetworkopen.2020.16197 32902652 PMC7489869

[20] Weir MA, Herzog CA. Beta blockers in patients with end-stage renal disease-Evidence-based recommendations. Semin Dial. 2018;31(3):219–25. doi: 10.1111/sdi.12691 29482260

[21] Chatzidou S, Kontogiannis C, Tsilimigras DI, Georgiopoulos G, Kosmopoulos M, Papadopoulou E, et al. Propranolol Versus Metoprolol for Treatment of Electrical Storm in Patients With Implantable Cardioverter-Defibrillator. J Am Coll Cardiol. 2018;71(17):1897–906. doi: 10.1016/j.jacc.2018.02.056 29699616

[22] Peck KY, Andrianopoulos N, Dinh D, Roberts L, Duffy SJ, Sebastian M, et al. Role of beta blockers following percutaneous coronary intervention for acute coronary syndrome. Heart. 2021;107(9):728–33. doi: 10.1136/heartjnl-2020-316605 32887736

[23] Wu PH, Lin YT, Kuo MC, Liu JS, Tsai YC, Chiu YW, et al. β-blocker dialyzability and the risk of mortality and cardiovascular events in patients undergoing hemodialysis. Nephrol Dial Transplant. 2020;35(11):1959–65. doi: 10.1093/ndt/gfaa058 32719861

[24] DiNicolantonio JJ, Lavie CJ, Fares H, Menezes AR, O’Keefe JH. Meta-analysis of carvedilol versus beta 1 selective beta-blockers (atenolol, bisoprolol, metoprolol, and nebivolol). Am J Cardiol. 2013;111(5):765–9. doi: 10.1016/j.amjcard.2012.11.031 23290925

[25] Nowicki M, Miszczak-Kuban J. Nonselective Beta-adrenergic blockade augments fasting hyperkalemia in hemodialysis patients. Nephron. 2002;91(2):222–7. doi: 10.1159/000058396 12053057

[26] Dickersin K, Min YI, Meinert CL. Factors influencing publication of research results. Follow-up of applications submitted to two institutional review boards. JAMA. 1992;267(3):374–8. 10.1001/jama.1992.03480030052036 1727960

[27] Guyatt GH, Oxman AD, Montori V, Vist G, Kunz R, Brozek J, et al. GRADE guidelines: 5. Rating the quality of evidence—publication bias. J Clin Epidemiol. 2011;64(12):1277–82. doi: 10.1016/j.jclinepi.2011.01.011 21802904

