# Supplementary material for: Cardio-selective versus non-selective β-blockers for cardiovascular events and mortality in long-term dialysis patients: A systematic review and meta-analysis
Source: PLoS One. 2022 Dec 19;17(12):e0279171. doi: 10.1371/journal.pone.0279171 (PMC9762568; doi:10.1371/journal.pone.0279171)
Supplement: S1 File — (DOCX) [file pone.0279171.s001.docx]

eTable 1. Search Strategy details

Pubmed (Up to Sep 04, 2022)

| # | Searches | Results |
| --- | --- | --- |
| 1 | Search dialysis [mh] | 146,066 |
| 2 | Search dialysis [Title/Abstract] | 115,492 |
| 3 | Search (((((((Dialyses, Renal[Title/Abstract]) OR Renal Dialyses[Title/Abstract]) OR Renal Dialyses[Title/Abstract]) OR Hemodialyses[Title/Abstract]) OR Hemodialysis[Title/Abstract]) OR Dialysis, Extracorporeal[Title/Abstract]) OR Dialyses, Extracorporeal[Title/Abstract]) | 118,008 |
| 4 | Search (1 or 2 or 3) | 212,233 |
| 5 | Search Adrenergic Antagonists [mh] | 58,318 |
| 6 | Search (((Antagonists, Adrenergic[Title/Abstract]) OR Antagonists, Adrenergic Receptor[Title/Abstract]) OR Adrenomimetics[Title/Abstract]) OR Receptor Antagonists, Adrenergic [Title/Abstract]) | 115 |
| 7 | Search ((non-cardioselect*[ Title/Abstract]) OR no* cardio-select* [Title/Abstract]) | 96 |
| 8 | Search ((((Bucindolol[Title/Abstract]) OR Carvedilol [Title/Abstract]) OR Labetalol[Title/Abstract]) OR Arotinolol[Title/Abstract] OR Gencaro[Title/Abstract] OR Almarl[Title/Abstract] OR (Carteolol[Title/Abstract])) OR (Nadolol[Title/Abstract])) OR (Penbutolol[Title/Abstract])) OR (Pindolol[Title/Abstract])) OR (Propranolol[Title/Abstract])) OR (Sotalol[Title/Abstract])) OR (Timolol[Title/Abstract]))) | 48,449 |
| 9 | Search (5 or 6 or 7 or 8) | 94,633 |
| 10 | Search Adrenergic beta1- Antagonists [mh] | 1,329 |
| 11 | Search (((Adrenergic beta 1 Receptor Antagonist* [Title/Abstract]) OR Adrenergic beta1-Antagonists [Title/Abstract]) OR beta1- Antagonists, Adrenergic [Title/Abstract]) | 19 |
| 12 | Search ((((cardioselect*[ Title/Abstract]) OR cardio-select* [Title/Abstract]) OR cardio* select*[ Title/Abstract]) | 1,659 |
| 13 | Search ((((Metoprolol [Title/Abstract]) OR Betaxolol[Title/Abstract]) OR Atenolol[Title/Abstract]) OR Esmolol[Title/Abstract] OR Celiprolol[Title/Abstract] OR Acebutolol[Title/Abstract] OR Bisoprolol[Title/Abstract] OR Nebivolol[Title/Abstract]) | 18,992 |
| 14 | Search (10 or 11 or 12 or 13) | 20,162 |
| 15 | Search (4 and 9 and 14) | 263 |

Embase (Up to Sep 04, 2022)

| # | Searches | Results |
| --- | --- | --- |
| 1 | ' dialysis '/exp | 319,254 |
| 2 | 'dialyses, renal':ab,ti OR 'renal dialyses':ab,ti OR hemodialyses:ab,ti OR hemodialysis:ab,ti OR 'dialysis, extracorporeal':ab,ti OR 'dialyses, extracorporeal':ab,ti | 103,185 |
| 3 | 1 or 2 | 336,424 |
| 4 | ' Adrenergic Antagonists '/exp | 591,450 |
| 5 | ' Antagonists, Adrenergic*':ab,ti OR ' Antagonists, Adrenergic Receptor':ab,ti OR 'Adrenomimetics' OR ' Receptor Antagonists, Adrenergic ':ab,ti | 126 |
| 6 | ' non-cardioselect*':ab,ti OR ' no* cardio-select*':ab,ti | 166 |
| 7 | ' Bucindolol ':ab,ti OR ' Carvedilol ':ab,ti OR ' Arotinolol ':ab,ti OR ' Labetalol ':ab,ti OR ' Gencaro ':ab,ti OR ' Almarl ':ab,ti OR ' Carteolol ':ab,ti OR ' Nadolol ':ab,ti OR ' Penbutolol ':ab,ti OR ' Pindolol ':ab,ti OR ' Propranolol ':ab,ti OR ' Sotalol ':ab,ti OR ' Timolol ':ab,ti | 63,268 |
| 8 | 4 or 5 or 6 or 7 | 595,708 |
| 9 | 'cardioselect*':ab,ti OR 'cardio-select* ':ab,ti OR 'cardio* select*':ab,ti | 2,366 |
| 10 | 'Metoprolol':ab,ti OR 'Betaxolol':ab,ti OR 'Atenolol':ab,ti OR 'Esmolol':ab,ti OR ' Celiprolol ':ab,ti OR ' Acebutolol ':ab,ti OR ' Bisoprolol ':ab,ti OR ' Nebivolol ':ab,ti | 28,282 |
| 11 | 9 or 10 | 29,494 |
| 12 | 3 AND 8 AND 11 | 385 |

Cochrane Library (Up to Sep 04, 2022)

| # | Searches | Results |
| --- | --- | --- |
| 1 | [mh " dialysis "] | 18,428 |
| 2 | (Dialyses, Renal OR Renal Dialyses OR Renal Dialyses OR Hemodialyses OR Hemodialysis OR Dialysis, Extracorporeal OR Dialyses, Extracorporeal):ti,ab,kw | 13,843 |
| 3 | #1 or #2 | 22,966 |
| 4 | [mh " Adrenergic Antagonists "] | 6,894 |
| 5 | (Antagonists Adrenergic* or Antagonists, Adrenergic Receptor or Adrenomimetics or Receptor Antagonists, Adrenergic):ti,ab,kw | 6,899 |
| 6 | ( Bucindolol or Carvedilol or Arotinolol or Labetalol or Gencaro or Almarl or Carteolol or Nadolol or Penbutolol or Pindolol or Propranolol or Sotalol or Timolol ):ti,ab,kw | 11,432 |
| 7 | (non-cardioselect* or no* cardio-select* or bucindolol or Carvedilol or arotinolol or labetalol or Gencaro or Almarl):ti,ab,kw | 2,553 |
| 8 | #4 or #5 or #6 or #7 | 16,095 |
| 9 | (Metoprolol or Betaxolol or Atenolol or Esmolol or Celiprolol or Acebutolol or Bisoprolol or Nebivolol):ti,ab,kw | 8,998 |
| 10 | (cardioselect* OR cardio-select* OR cardio* select*):ti,ab,kw | 18,344 |
| 11 | （#9 or #10）and #8 | 5,583 |
| 12 | animal*:ti,ab,kw | 40,495 |
| 13 | human*:ti,ab,kw | 1,168,335 |
| 14 | #12 not #13 | 5,157 |
| 15 | #3 and #11 | 65 |
| 16 | #15 not #14 | 65 |

ClinicalTrials.gov (Up to Sep 04, 2022)

| # | Searche Conditions | Results |
| --- | --- | --- |
| 1 | (Dialyses OR Hemodialyses) AND (Bucindolol OR Carvedilol OR Labetalol OR Arotinolol OR Gencaro OR Almarl OR Carteolol OR Nadolol OR Penbutolol OR Pindolol OR Propranolol OR Sotalol OR Timolol) | 3 |
| 2 | (Dialyses OR Hemodialyses) AND (Metoprolol OR Betaxolol OR Atenolol OR Esmolol OR Celiprolol OR Acebutolol OR Bisoprolol OR Nebivolol) | 2 |
| 3 | 1 OR 2 | 5 |
