# Supplementary material for: Cardio-selective versus non-selective β-blockers for cardiovascular events and mortality in long-term dialysis patients: A systematic review and meta-analysis
Source: PLoS One. 2022 Dec 19;17(12):e0279171. doi: 10.1371/journal.pone.0279171 (PMC9762568; doi:10.1371/journal.pone.0279171)
Supplement: S3 File — (PDF) [file pone.0279171.s003.pdf]

Clinical Kidney Journal, 2021, vol. 14, no. 3, 983–990

doi: 10.1093/ckj/sfaa248
Advance Access Publication Date: 4 January 2021
Original Article

ORIGINAL ARTICLE

Comparative effectiveness of bisoprolol and carvedilol among patients receiving maintenance hemodialysis

Ping-Hsun Wu [1,2,3], Yi-Ting Lin [2,3,4], Jia-Sin Liu [5], Yi-Chun Tsai [1,3,6,7], Mei-Chuan Kuo [1,3,7], Yi-Wen Chiu [1,3,7], Shang-Jyh Hwang [1,3,7,8] and Juan-Jesus Carrero [9]

[1] Division of Nephrology, Department of Internal Medicine, Kaohsiung Medical University Hospital, Kaohsiung Medical University, Kaohsiung, Taiwan, [2]Institute of Clinical Medicine, College of Medicine, Kaohsiung Medical University, Kaohsiung, Taiwan, [3]Faculty of Medicine, College of Medicine, Kaohsiung Medical University, Kaohsiung, Taiwan, [4]Department of Family Medicine, Kaohsiung Medical University Hospital, Kaohsiung Medical University, Kaohsiung, Taiwan, [5]Graduate Institute of Public Health, College of Health Science, Kaohsiung Medical University, Kaohsiung, Taiwan, [6]Division of General Medicine, Kaohsiung Medical University Hospital, Kaohsiung Medical University, Kaohsiung, Taiwan, [7]Faculty of Renal Care, College of Medicine, Kaohsiung Medical University, Kaohsiung, Taiwan, [8]Institute of Population Sciences, National Health Research Institutes, Miaoli, Taiwan and [9]Department of Medical Epidemiology and Biostatistics, Karolinska Institutet, Stockholm, Sweden

Correspondence to: Yi-Wen Chiu; E-mail: chiuyiwen@kmu.edu.tw

ABSTRACT

Background. Despite widespread use, there is no trial evidence to inform β-blocker's (BB) relative safety and efficacy among patients undergoing hemodialysis (HD). We herein compare health outcomes associated with carvedilol or bisoprolol use, the most commonly prescribed BBs in these patients.

Methods. We created a cohort study of 9305 HD patients who initiated bisoprolol and 11 171 HD patients who initiated carvedilol treatment between 2004 and 2011. We compared the risk of all-cause mortality and major adverse cardiovascular events (MACEs) between carvedilol and bisoprolol users during a 2-year follow-up.

Results. Bisoprolol initiators were younger, had shorter dialysis vintage, were women, had common comorbidities of hypertension and hyperlipidemia and were receiving statins and antiplatelets, but they had less heart failure and digoxin prescriptions than carvedilol initiators. During our observations, 1555 deaths and 5167 MACEs were recorded. In the multivariable-adjusted Cox model, bisoprolol initiation was associated with a lower all-cause mortality {hazard ratio [HR] 0.66 [95% confidence interval (CI) 0.60–0.73]} compared with carvedilol initiation. After accounting for the competing risk of death, bisoprolol use (versus carvedilol) was associated with a lower risk of MACEs [HR 0.85 (95% CI 0.80–0.91)] and attributed to a lower risk of heart failure [HR 0.83 (95% CI 0.77–0.91)] and ischemic stroke [HR 0.84 (95% CI 0.72–0.97)], but not

Received: 2.8.2020; Editorial decision: 26.10.2020

© The Author(s) 2021. Published by Oxford University Press on behalf of ERA-EDTA.
This is an Open Access article distributed under the terms of the Creative Commons Attribution Non-Commercial License (http://creativecommons.org/licenses/by-nc/4.0/), which permits non-commercial re-use, distribution, and reproduction in any medium, provided the original work is properly cited. For commercial re-use, please contact journals.permissions@oup.com

Clinical Kidney Journal

to differences in the risk of acute myocardial infarction [HR 1.03 (95% CI 0.93–1.15)]. Results were confirmed in propensity score matching analyses, stratified analyses and analyses that considered prescribed dosages or censored patients discontinuing or switching BBs.

Conclusions. Relative to carvedilol, bisoprolol initiation by HD patients was associated with a lower 2-year risk of death and MACEs, mainly attributed to lower heart failure and ischemic stroke risk.

Keywords: acute coronary syndrome, bisoprolol, cardiovascular event, carvedilol, heart failure, hemodialysis, mortality, stroke

INTRODUCTION

Persons with end-stage kidney disease (ESKD) undergoing dialysis are at high risk of developing and dying from cardiovascular (CV) disease [1], for which they often receive CV prevention medications. β-blockers (BBs) are the most commonly used CV medications in hemodialysis (HD) patients, despite scarce interventional evidence on their benefit in them [2–4]. To the best of our knowledge, only two small trials have evaluated BB safety and efficacy in HD patients. First, carvedilol compared with placebo was associated with improved survival among 114 HD patients with dilated cardiomyopathy [5]. Second, atenolol had a lower risk of CV events than lisinopril in 200 HD patients with hypertension and left ventricular hypertrophy [6].

The BB class is heterogeneous with respect to pharmacodynamics and pharmacokinetics. Network meta-analyses of BB trials in patients with heart failure suggest that there are no obvious differences when comparing the different BBs for the risk of death, sudden cardiac death, death due to pump failure or drug discontinuation [7]. However, this may not be the case in patients undergoing HD, given both the unique risk profile of these patients [8, 9] and the possibility that BBs hemodialytic clearance may influence their effectiveness [10, 11]. A large retrospective US cohort study showed that new carvedilol users had an increased risk of 1-year all-cause and CV mortality compared with new metoprolol users [12]. The relative effectiveness of other BBs is unknown and knowledge of these potential differences may support clinicians in their day-to-day decisions [13].

In many health systems [11, 14–16], bisoprolol and carvedilol are the two most commonly used BBs. Bisoprolol may offer advantages over carvedilol because of its β_1 selectivity [9] and moderate dialyzability with less intradialytic hypotension potential [10, 12]. The objective of this study was to evaluate the risk for all-cause mortality and CV events associated with bisoprolol compared with carvedilol in HD patients.

MATERIALS AND METHODS

Study design and data sources

All HD subjects were registered in the Taiwan National Health Insurance Research Database (NHIRD) [17]. The inclusion in the dialysis register requires a medical examination by two nephrologists that investigate underlying disease, laboratory data, renal ultrasonography and indications for dialysis treatment. Diagnosis of HD was confirmed by International Classification of Diseases, Ninth Revision code 585, consecutive HD procedure codes and inclusion in the Catastrophic Illness Patient Database. For this study we enrolled all adult (>18 years) patients who underwent chronic maintenance HD for >90 days with BB use ($n = 58\,476$) between 1 January 2004 and 31 December 2011. We selected those who initiated bisoprolol or carvedilol therapy after HD initiation (identified as the first prescription post-dialysis with an absence of any other BB prescription in the previous 90 days). The date of bisoprolol or carvedilol prescription was set as the index date (Supplementary data, Figure S1). Furthermore, to assess the dose effect, we analyzed the risk of outcomes according to the dose groups as per heart failure guidelines [18, 19] during the 90-day exposure period. The study subjects were assigned to one of the following groups: high-dose bisoprolol (≥10 mg/day), low-dose bisoprolol (≥1.25–<10 mg/day), high-dose carvedilol (≥50 mg/day) and low-dose carvedilol (≥6.25–<50 mg/day).

Study covariates

Comorbidities were defined by the presence of at least one hospital discharge or three consistent diagnoses in medical records during the 180-day time window before the index date. Comorbidities included diabetes mellitus, hypertension, hyperlipidemia, coronary artery disease, myocardial infarction, heart failure, peripheral vascular disease, cerebrovascular disease and tachyarrhythmias (Supplementary data, Table S1). Concurrent medications, including renin–angiotensin–aldosterone system inhibitors, calcium channel blockers, warfarin, statins, digoxin and antiplatelets (aspirin or clopidogrel), were identified by Anatomical Therapeutic Chemical codes (Supplementary data, Table S2).

Study outcomes and follow-up

The main study outcomes were all-cause mortality and major adverse CV events (MACEs). A MACE was defined as a hospital admission with a primary diagnosis of acute myocardial infarction, heart failure or ischemic stroke. All outcome definitions are detailed in Supplementary data, Table S1. Information on deaths was collected from the Catastrophic Illness Database. Patients were followed up until death, deregistration, dialysis modality change, renal transplantation, events or until 2 years from the index date, whichever occurred first. Our main analysis followed an intention-to-treat (ITT) design, whereby we assumed that the patient remained on therapy until the event or end of follow-up.

Statistical analysis

Data are presented as the mean [standard deviation (SD)] for normally distributed continuous variables and proportions for categorical variables. Kaplan–Meier curves were generated showing cumulative probabilities of study outcomes over the 2-year observation time and differences were tested using a log-rank test in the full cohort. After ensuring the fulfillment of proportional hazards assumptions by the Schoenfeld residuals trend test, we applied univariate and multivariable Cox proportional hazards regression for the study of all-cause mortality

associated with bisoprolol or carvedilol use. Covariates included in the multivariable adjustments were age, sex, dialysis vintage, comorbidities and use of concomitant medications. Because dialysis patients are at high risk of death, we applied competing risk analyses to estimate the associated risks of nonfatal events (MACEs and single vascular events) using cause-specific hazard regression. Our main analysis followed an ITT design whereby we assumed that the patient remained on therapy until the event or the end of follow-up.

To address confounding by indication resulting from non-random treatment allocation, 1:1 propensity score (PS) matching [20, 21] was performed to balance confounders (age, sex, dialysis vintage, comorbidities and concomitant medications) between bisoprolol and carvedilol users. We created PS-matched pairs with the Mahalanobis metric method [22, 23] without replacement by the nearest number matching with a caliper of 0.0001. Baseline characteristics were then compared before and after PS matching using a standardized mean difference. A standardized mean difference <0.1 was considered to indicate an adequate balance in variables between groups. A Cox regression model and cause-specific hazard regression were applied for all-cause mortality and MACEs, respectively.

In order to evaluate the robustness of our findings, we performed various sensitivity analyses, including subgroup analyses stratifying by baseline coronary artery disease or heart failure comorbidity, modification of our definition of new user as the first identified prescription post-dialysis with an absence of any other BB prescription in the previous 120 and 180 days, censoring patients at the time of BB treatment discontinuation or switching to another BB during follow-up (as-treated analyses), excluding subjects who switched BBs during follow-up and, to assess the impact of residual confounding [24, 25], by applying the E-value methodology [26]. The E-value identifies the minimum strength of the association that unmeasured confounders would need to have with both treatment and outcome, conditional on the measured covariates, to explain the observed association fully. This estimates what the relative risk would have to be for any unmeasured confounder to overcome the observed association of BB with death or MACEs [26]. All analyses were performed using Stata version 14 (StataCorp, College Station, TX, USA). A two-tailed P-value <0.05 was considered significant.

RESULTS

Patient characteristics

During 2004–11, a total of 58 476 patients initiated dialysis treatment in Taiwan. After excluding prevalent BB users ($n = 18\,240$), there were 36 603 patients who initiated BB after incident dialysis treatment. From this pool we then identified patients initiating bisoprolol ($n = 9305$) or carvedilol ($n = 11\,171$) (Figure 1).

The characteristics of the included patients are listed in Table 1. Before PS matching, patients receiving bisoprolol were younger, had shorter dialysis vintage, were more often women, had a higher proportion of hypertension and hyperlipidemia and more commonly used statins and antiplatelets than patients receiving carvedilol. Conversely, carvedilol users had a higher proportion of heart failure and more often used digoxin compared with bisoprolol users. PS matching resulted in 4107 matched pairs with well-balanced baseline characteristics (all standardized differences <0.1). The mean patient age was 55.4 ± 12.3 years, 51.6% were men, 39.9% had diabetes and 78.6% had hypertension (Table 1).

Primary analysis

In the full cohort ($n = 20\,476$), the mean follow-up time was 1.79 years in the bisoprolol group and 1.39 years in the carvedilol group. During this period, 1555 deaths and 5167 MACEs were recorded (Supplementary data, Table S3). Kaplan–Meier curves graphically showed a lower incidence of all-cause mortality and MACEs among patients taking bisoprolol compared with carvedilol users (Figure 2). After multivariable adjustment, patients initiating bisoprolol had a lower mortality risk {adjusted hazard ratio [HR] 0.66 [95% confidence interval (CI) 0.60–0.73]} compared with patients using carvedilol (Table 2). Using cause-specific hazards analysis, users of bisoprolol were at a lower risk of MACEs [HR 0.85 (95% CI 0.80–0.91)], mainly attributed to a lower risk of heart failure [HR 0.83 (95% CI 0.77–0.91)] and ischemic stroke [HR 0.84 (95% CI 0.72–0.97)] (Table 2). No suggestion for heterogeneity was observed in the stratified analysis (Supplementary data, Table S4). Compared with patients who took low-dose carvedilol, those who were prescribed low- or high-dose bisoprolol had a lower risk of all-cause mortality, MACEs and heart failure, but did not differ in their risk of myocardial infarction (Table 3).

Secondary analysis: PS-matched cohort

We identified 4107 pairs of new users of carvedilol and bisoprolol with comparable characteristics as identified through PS matching (Table 1). Compared with carvedilol initiators, bisoprolol initiators had a lower risk of all-cause mortality, MACEs, heart failure and ischemic stroke (Table 2).

Sensitivity analyses

Redefining a new user in our study with larger predispensation windows (120 and 180 days) yielded similar results as the main analysis (Supplementary data, Tables S5 and S6). Censoring at the time of bisoprolol or carvedilol discontinuation/switch (Supplementary data, Table S7), as well as excluding these patients (Supplementary data, Table S8), yielded similar results to our primary analysis.

E-values suggested that unmeasured confounding of considerable strength would be needed to fully explain the observed associations in the unmatched cohort with the multivariable-adjusted model (Supplementary data, Table S9); for example, E-values of bisoprolol compared with carvedilol indicated that the observed HR of 0.66 for all-cause mortality could only be explained by unmeasured confounders that were associated with both initiation of bisoprolol and risk of death by a risk ratio >2.40 over that of the confounders that were measured in this study (upper confidence bound 2.08). The adjusted HRs of most covariates in our multivariable model lie below this value. For example, the adjusted HR was 1.58 for diabetes mellitus and 1.42 for CV disease.

DISCUSSION

In persons with ESKD undergoing dialysis, both interventional [5, 27] and observational studies [3, 14, 28] agree that BBs, compared with nonuse, offer cardioprotection and improved survival. However, BB classes possess different pharmacologic and pharmacokinetic properties, vasodilatory capabilities and beta-adrenergic receptor selectivity [9, 11, 12] that may alter risk–benefit profiles. As emphasized by recent HD guidelines, comparative effectiveness studies on CV medications are needed to inform treatment decisions [13]. Nevertheless,

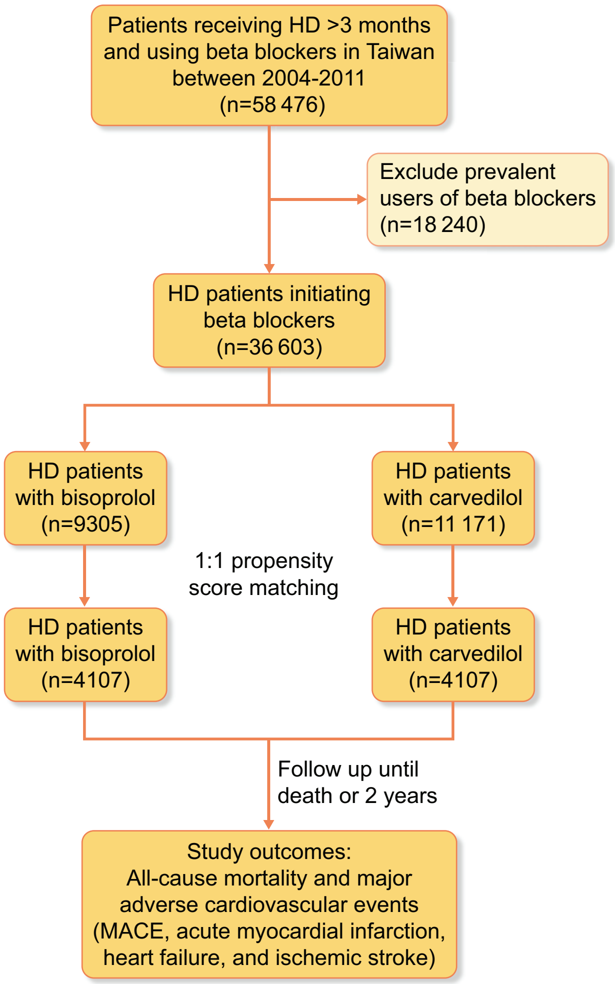

FIGURE 1: Study design and patient selection flow chart.

recruiting patients for such trials has proved challenging, resulting in the premature termination of the BB to LOwer CV Dialysis Events (BLOCADE) trial [29] and accentuating the need to rely on observational studies from routine clinical practice.

Bisoprolol and carvedilol are two of the most commonly used BBs for HD patients in many countries [11, 14–16], but we are not aware of previous studies comparing their relative efficacy. In this nationwide study, we observed that relative to carvedilol, bisoprolol initiation was associated with a 20% lower risk of all-cause mortality and a 13% lower risk of MACEs. Supporting this observation, we also found that both low-dose bisoprolol (≥1.25–<10 mg/day) or high-dose bisoprolol (≥10 mg/day) were associated with a lower risk of all-cause mortality, MACEs and heart failure compared with low-dose carvedilol (≥6.25–<50 mg/day). However, we did not find a dose-dependent protective effect for bisoprolol. Because bisoprolol exerts a strong β_1 selectivity effect that is estimated as 10-fold higher than that of propranolol [9, 30], it is possible that even at regular or low doses, bisoprolol may provide a benefit. Further supporting our observations, cardioselective BBs (atenolol and metoprolol) were found to reduce both all-cause and CV mortality compared with nonselective BBs (carvedilol and labetalol) in a cohort of 4398 incident US hemodialysis and peritoneal dialysis patients [31]. Another carefully designed pharmacoepidemiological analysis from the USA noted that carvedilol initiation had slightly increased rates of all-cause, CV mortality and intradialytic hypotension than metoprolol initiation [12]. Unfortunately, the infrequent use of metoprolol in our study prevented us from confirming or refuting that study.

This is an observational study, and despite our careful design to avoid biases, residual confounding and confounding by indication may explain the observed differences between BBs. Nonetheless, there are differences in bisoprolol and carvedilol pharmacokinetics that provide a rationale in support of bisoprolol's observed superiority. Both drugs lower cardiac contractility and heart rate, but carvedilol has, in addition, α-blocking effects that confer increased vasodilation [32]. It has been proposed that carvedilol's α blockade may increase the risk of intradialytic

Table 1. Baseline characteristics of HD patients initiating bisoprolol or carvedilol before and after PS matching

	Full cohort			1:1 PS matched cohort		
Baseline characteristics	Bisoprolol (*n* = 9305)	Carvedilol (*n* = 11 171)	Standardized differences	Bisoprolol (*n* = 4107)	Carvedilol (*n* = 4107)	Standardized differences
Age (years), mean ± SD	56.3 ± 13.0	57.1 ± 13.1	0.06	55.4 ± 12.3	55.3 ± 12.3	0.003
Men, *n* (%)	4716 (50.7)	5853 (52.4)	0.034	2121 (51.6)	2124 (51.7)	0.001
Dialysis vintage (years), mean ± SD	4.72 ± 2.65	4.89 ± 2.72	0.061	5 ± 2.69	5.26 ± 2.76	0.096
Comorbidities, *n* (%)						
Diabetes mellitus	4581 (49.2)	5536 (49.6)	0.007	1640 (39.9)	1638 (39.9)	0.001
Hypertension	7313 (78.6)	8476 (75.9)	0.065	3228 (78.6)	3226 (78.5)	0.001
Hyperlipidemia	2063 (22.2)	2270 (20.3)	0.045	442 (10.8)	444 (10.8)	0.002
Coronary artery disease[a]	3374 (36.3)	4122 (36.9)	0.013	955 (23.3)	952 (23.2)	0.002
Myocardial infarction	830 (8.9)	949 (8.5)	0.015	131 (3.2)	129 (3.1)	0.003
Heart failure	2391 (25.7)	3348 (30.0)	0.095	731 (17.8)	729 (17.8)	0.001
Peripheral vascular disease	615 (6.6)	718 (6.4)	0.007	54 (1.3)	51 (1.2)	0.007
Cerebrovascular disease	1059 (11.4)	1265 (11.3)	0.002	113 (2.8)	108 (2.6)	0.008
Tachyarrhythmias[b]	418 (4.5)	448 (4.0)	0.024	155 (3.8)	116 (2.8)	0.053
Concomitant drugs, *n* (%)						
RAAS inhibitors	1157 (12.4)	1296 (11.6)	0.026	205 (5.0)	203 (4.9)	0.002
Calcium channel blockers	588 (6.3)	683 (6.1)	0.008	39 (0.9)	39 (0.9)	0
Warfarin	134 (1.4)	149 (1.3)	0.009	32 (0.8)	28 (0.7)	0.011
Statins	1988 (21.4)	2082 (18.6)	0.068	405 (9.9)	404 (9.8)	0.001
Digoxin	190 (2.0)	290 (2.6)	0.037	56 (1.4)	62 (1.5)	0.012
Antiplatelets (aspirin, clopidogrel)	2530 (27.2)	2860 (25.6)	0.036	496 (12.1)	492 (12.0)	0.003
PS probability, mean ± SD	0.46 ± 0.04	0.45 ± 0.04	0.162	0.45 ± 0.03	0.45 ± 0.03	0

[a]Coronary artery disease includes myocardial infarction, history of percutaneous coronary interventions and history of coronary artery bypass surgery.
[b]Tachyarrhythmias included paroxysmal supraventricular tachycardia, atrial flutter and atrial fibrillation.
RAAS: renin–angiotensin–aldosterone system.

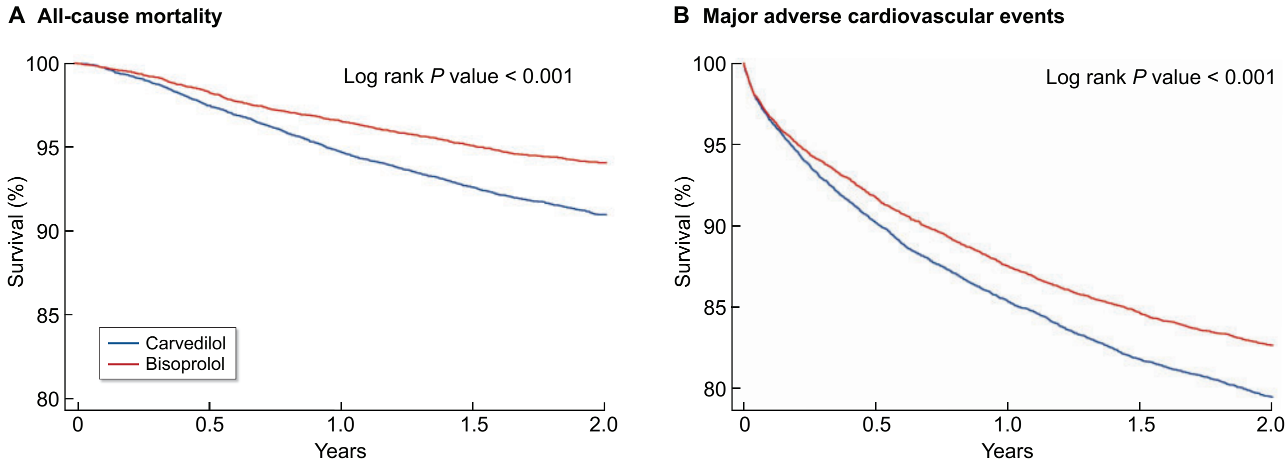

FIGURE 2: Kaplan–Meier curves for the incidence of (**A**) all-cause death and (**B**) MACEs according to the initiation of bisoprolol or carvedilol in patients undergoing HD (full cohort).

hemodynamic instability during HD because it inhibits the compensatory effect of sympathetic nervous system–mediated peripheral vasoconstriction [12]. Unfortunately, we lack records of intradialytic blood pressure in our study to test this hypothesis. On the other hand, the removal of BBs by HD may also affect intradialytic blood pressure. While carvedilol is poorly dialyzed, bisoprolol is moderately dialyzed [10]. Thus the blood pressure–lowering effects of carvedilol may persist throughout the dialysis course, whereas the blood pressure–lowering effects of bisoprolol may be reduced as circulating drug concentrations decrease during HD therapy [33–36]. Although carvedilol has antioxidant properties and a good metabolic profile compared with other BBs [37, 38], the hypotensive side effects of carvedilol exacerbated by marked fluctuations in extracellular fluid volume in HD may counteract the CV protection benefit. In a secondary analysis of BLOCADE, treatment with carvedilol did not modify surrogate cardiac biomarkers, but instead increased both brain natriuretic peptide and N-terminal pro-B-type natriuretic peptide natriuretic peptide levels [39].

Strengths and limitations

This study has several strengths, including large sample sizes, comprehensive follow-up and outcome applicable to real-world

Table 2. Outcomes associated with the initiation of bisoprolol versus carvedilol in patients undergoing HD (ITT analysis)

		HR (95% CI)		
Main outcomes	Full cohort, no. of events/no. of subjects	Full cohort, crude ($n = 20\,476$)	Full cohort, adjusted[b] ($n = 20\,476$)	1:1 PS-matched cohort ($n = 8214$)
All-cause mortality				
Bisoprolol	550/9305	0.65 (0.58–0.72)	0.66 (0.60–0.73)	0.80 (0.67–0.96)
Carvedilol	1005/11 171	1 (Reference)	1 (Reference)	1 (Reference)
MACE[a]				
Bisoprolol	2184/9305	0.83 (0.78–0.89)	0.85 (0.80–0.91)	0.87 (0.77–0.98)
Carvedilol	2983/11 171	1 (Reference)	1 (Reference)	1 (Reference)
Single MACE				
Acute myocardial infarction				
Bisoprolol	789/9305	1.01 (0.91–1.13)	1.03 (0.93–1.15)	1.03 (0.85–1.26)
Carvedilol	941/11 171	1 (Reference)	1 (Reference)	1 (Reference)
Heart failure				
Bisoprolol	1560/9305	0.80 (0.73–0.86)	0.83 (0.77–0.91)	0.81 (0.71–0.94)
Carvedilol	2182/11 171	1 (Reference)	1 (Reference)	1 (Reference)
Ischemic stroke				
Bisoprolol	366/9305	0.82 (0.71–0.95)	0.84 (0.72–0.97)	0.70 (0.54–0.91)
Carvedilol	509/11 171	1 (Reference)	1 (Reference)	1 (Reference)

[a]MACE events included myocardial infarction, heart failure hospitalization and ischemic stroke. Major CV outcomes and single CV outcomes were analyzed by a cause-specific hazard model as a competing risk model.
[b]The multivariable-adjusted model was obtained from Cox regression models adjusted for age, sex, dialysis vintage, comorbidities and concomitant medications.

Table 3. Outcomes associated with carvedilol or bisoprolol use by prescribed dose categories (ITT analysis)

		HR (95% CI)	
Outcomes	No. of events/no. of subjects	Crude	Multivariable adjusted model[b]
All-cause mortality			
High-dose bisoprolol	193/2870	0.73 (0.63–0.86)	0.81 (0.69–0.94)
Low-dose bisoprolol	357/6435	0.60 (0.53–0.68)	0.61 (0.54–0.69)
High-dose carvedilol	27/358	0.83 (0.57–1.22)	1.32 (0.90–1.94)
Low-dose carvedilol	978/10 813	1 (Reference)	1 (Reference)
MACE[a]			
High-dose bisoprolol	558/2870	0.75 (0.68–0.83)	0.88 (0.79–0.97)
Low-dose bisoprolol	1626/6435	0.85 (0.79–0.92)	0.84 (0.78–0.91)
High-dose carvedilol	54/358	0.58 (0.43–0.79)	0.87 (0.64–1.17)
Low-dose carvedilol	2929/10 813	1 (Reference)	1 (Reference)
Acute myocardial infarction			
High-dose bisoprolol	200/2870	0.92 (0.78–1.08)	1.07 (0.90–1.26)
Low-dose bisoprolol	589/6435	1.04 (0.92–1.17)	1.02 (0.91–1.15)
High-dose carvedilol	16/358	0.65 (0.39–1.06)	1.01 (0.62–1.66)
Low-dose carvedilol	925/10 813	1 (Reference)	1 (Reference)
Heart failure			
High-dose bisoprolol	379/2870	0.70 (0.62–0.80)	0.84 (0.74–0.96)
Low-dose bisoprolol	1181/6 435	0.82 (0.75–0.90)	0.83 (0.75–0.90)
High-dose carvedilol	36/358	0.50 (0.34–0.75)	0.78 (0.52–1.16)
Low-dose carvedilol	2146/10 813	1 (Reference)	1 (Reference)
Ischemic stroke			
High-dose bisoprolol	102/2870	0.80 (0.64–1.01)	0.88 (0.70–1.10)
Low-dose bisoprolol	264/6435	0.82 (0.69–0.97)	0.82 (0.70–0.97)
High-dose carvedilol	11/358	0.71 (0.38–1.33)	0.99 (0.53–1.85)
Low-dose carvedilol	498/10 813	1 (Reference)	1 (Reference)

BB dosage definition: high-dose bisoprolol, ≥10 mg/day; low-dose bisoprolol, ≥1.25–<10 mg/day; high-dose carvedilol, ≥50 mg/day; low-dose carvedilol, ≥6.25–<50 mg/day). Low-dose carvedilol users are the reference group.
[a]Major CV events included myocardial infarction, heart failure hospitalization and ischemic stroke. Major CV outcome was analyzed by a cause-specific hazard model as a competing risk model.
[b]Multivariable adjusted model was obtained from Cox regression models adjusted for age, sex, dialysis vintage, comorbidities and concomitant medications.

Clinical Kidney Journal

practice. However, this study also has limitations. Despite careful control for potential confounders, observational studies can never eliminate the influence of indication bias, coding errors or misdiagnoses in administrative records. This being said, the diagnostic accuracy of NHIRD claims was previously found be high for our study outcomes [17, 40, 41]. Some confounders were not available in our study and must be acknowledged, including body mass index, heart rate, blood pressure, echocardiography parameters, lifestyle and actual drug utility time. Because of this, the bisoprolol or carvedilol effect on HD patients with heart failure with preserved ejection fraction could not be evaluated. Our efforts to quantify this risk of residual confounding (falsification outcomes and E value estimations) suggest, however, that this bias risk is moderately low. In addition, although we tried to evaluate the dose–effect between bisoprolol and carvedilol, we still need to acknowledge that BB prescription does not guarantee that the patient complies with the treatment. Finally, our study pertains to Taiwan healthcare in a population of Asian ethnicity. Extrapolation to other practices and populations should be done with caution.

CONCLUSION

Our study showed that relative to carvedilol, bisoprolol initiation in patients undergoing HD was associated with a lower risk of death and MACEs. While our findings may inform clinical decisions regarding the choice of BBs in this high-risk population. In the absence of trial evidence, this study may inform the choice of BB therapy in this high-CV-risk population with a void of evidence.

SUPPLEMENTARY DATA

Supplementary data are available at *ckj* online.

ACKNOWLEDGEMENTS

This study is based in part on data from the NHIRD provided by the Bureau of National Health Insurance, Department of Health and managed by the National Health Research Institutes. The interpretation and conclusions contained herein do not represent the views of the Bureau of National Health Insurance, Department of Health or National Health Research Institutes.

FUNDING

This work was supported by grants from the Swedish Research Council (grant 2019-01059), the Swedish Heart and Lung Foundation, Kaohsiung Medical University (KMU-Q108024) and Kaohsiung Medical University Hospital (KMUH108-8M11, KMUH107-7R16, KMUH106-6T03, KMUH 106-6R17, KMUH104-4R11 and KMUH103-3R10).

AUTHORS' CONTRIBUTIONS

P.H.W. and Y.T.L. had full access to all of the data in the study and take responsibility for the integrity of the data and the accuracy of the data analysis. P.H.W., Y.T.L. and J.J.C. were involved in the study concept and design. P.H.W, Y.T.L. and J.S.L. were responsible for the acquisition, analysis and interpretation of data. P.H.W. and Y.T.L. drafted the manuscript. Y.C.T., M.C.K., Y.W.C. and S.J.H. were responsible for critical revision of the manuscript for important intellectual content. Y.T.L and J.S.L. were responsible for the statistical analysis. P.H.W. and Y.W.C. obtained funding. J.S.L., M.C.K. and Y.W.C. were responsible for administrative, technical or material support. Y.W.C., S.J.H. and J.J.C were responsible for supervision.

CONFLICT OF INTEREST STATEMENT

None declared.

REFERENCES

1. Jha V, Garcia-Garcia G, Iseki K *et al.* Chronic kidney disease: global dimension and perspectives. *Lancet* 2013; 382: 260–272
2. Frankenfield DL, Weinhandl ED, Powers CA *et al.* Utilization and costs of cardiovascular disease medications in dialysis patients in Medicare part D. *Am J Kidney Dis* 2012; 59: 670–681
3. Foley RN, Herzog CA, Collins AJ *et al.* Blood pressure and long-term mortality in United States hemodialysis patients: USRDS Waves 3 and 4 Study. *Kidney Int* 2002; 62: 1784–1790
4. Kitchlu A, Clemens K, Gomes T *et al.* Beta-blockers and cardiovascular outcomes in dialysis patients: a cohort study in Ontario, Canada. *Nephrol Dial Transplant* 2012; 27: 1591–1598
5. Cice G, Ferrara L, D'Andrea A *et al.* Carvedilol increases two-year survivalin dialysis patients with dilated cardiomyopathy: a prospective, placebo-controlled trial. *J Am Coll Cardiol* 2003; 41: 1438–1444
6. Agarwal R, Sinha AD, Pappas MK *et al.* Hypertension in hemodialysis patients treated with atenolol or lisinopril: a randomized controlled trial. *Nephrol Dial Transplant* 2014; 29: 672–681
7. Chatterjee S, Biondi-Zoccai G, Abbate A *et al.* Benefits of beta blockers in patients with heart failure and reduced ejection fraction: network meta-analysis. *BMJ* 2013; 346: f55
8. Shroff GR, Herzog CA. β-Blockers in dialysis patients: a nephrocardiology perspective. *J Am Soc Nephrol* 2015; 26: 774–776
9. Weir MA, Herzog CA. Beta blockers in patients with end-stage renal disease—evidence-based recommendations. *Semin Dial* 2018; 31: 219–225
10. Tieu A, Velenosi TJ, Kucey AS *et al.* β-Blocker dialyzability in maintenance hemodialysis patients: a randomized clinical trial. *Clin J Am Soc Nephrol* 2018; 13: 604–611
11. Weir MA, Dixon SN, Fleet JL *et al.* β-Blocker dialyzability and mortality in older patients receiving hemodialysis. *J Am Soc Nephrol* 2015; 26: 987–996
12. Assimon MM, Brookhart MA, Fine JP *et al.* A comparative study of carvedilol versus metoprolol initiation and 1-year mortality among individuals receiving maintenance hemodialysis. *Am J Kidney Dis* 2018; 72: 337–348
13. Levin NW, Kotanko P, Eckardt KU *et al.* Blood pressure in chronic kidney disease stage 5D-report from a Kidney Disease: Improving Global Outcomes controversies conference. *Kidney Int* 2010; 77: 273–284
14. Tang CH, Wang CC, Chen TH *et al.* Prognostic benefits of carvedilol, bisoprolol, and metoprolol controlled release/extended release in hemodialysis patients with heart failure: a 10-year cohort. *J Am Heart Assoc* 2016; 5: e002584
15. Lazarus DL, Jackevicius CA, Behlouli H *et al.* Population-based analysis of class effect of β blockers in heart failure. *Am J Cardiol* 2011; 107: 1196–1202
16. Frohlich H, Torres L, Tager T *et al.* Bisoprolol compared with carvedilol and metoprolol succinate in the treatment of

patients with chronic heart failure. *Clin Res Cardiol* 2017; 106: 711–721
17. Cheng CL, Kao YH, Lin SJ *et al*. Validation of the National Health Insurance Research Database with ischemic stroke cases in Taiwan. *Pharmacoepidem Drug Safe* 2011; 20: 236–242
18. Lindenfeld J, Albert NM Boehmer JP *et al*. HFSA 2010 comprehensive heart failure practice guideline. *J Card Fail* 2010; 16: e1–194
19. Su VY, Chang YS, Hu YW *et al*. Carvedilol, bisoprolol, and metoprolol use in patients with coexistent heart failure and chronic obstructive pulmonary disease. *Medicine (Baltimore)* 2016; 95: e2427
20. Sturmer T, Wyss R, Glynn RJ *et al*. Propensity scores for confounder adjustment when assessing the effects of medical interventions using nonexperimental study designs. *J Intern Med* 2014; 275: 570–580
21. Austin PC. The performance of different propensity score methods for estimating marginal hazard ratios. *Stat Med* 2013; 32: 2837–2849
22. Leacy FP, Stuart EA. On the joint use of propensity and prognostic scores in estimation of the average treatment effect on the treated: a simulation study. *Stat Med* 2014; 33: 3488–3508
23. D'Agostino RB Jr. Propensity score methods for bias reduction in the comparison of a treatment to a non-randomized control group. *Stat Med* 1998; 17: 2265–2281
24. Lipsitch M, Tchetgen Tchetgen E et al, . Negative controls: a tool for detecting confounding and bias in observational studies. *Epidemiology* 2010; 21: 383–388
25. Prasad V, Jena AB. Prespecified falsification end points: can they validate true observational associations? *JAMA* 2013; 309: 241–242
26. VanderWeele TJ, Ding P. Sensitivity analysis in observational research: introducing the e-value. *Ann Intern Med* 2017; 167: 268–274
27. Cice G, Ferrara L, Di Benedetto A *et al*. Dilated cardiomyopathy in dialysis patients—beneficial effects of carvedilol: a double-blind, placebo-controlled trial. *J Am Coll Cardiol* 2001; 37: 407–411
28. Abbott KC, Trespalacios FC, Agodoa LY *et al*. β-Blocker use in long-term dialysis patients: association with hospitalized heart failure and mortality. *Arch Intern Med* 2004; 164: 2465–2471
29. Roberts MA, Pilmore HL, Ierino FL *et al*. The β-Blocker to Lower Cardiovascular Dialysis Events (BLOCADE) feasibility study: a randomized controlled trial. *Am J Kidney Dis* 2016; 67: 902–911
30. Frishman WH. β-Adrenergic blockers: a 50-year historical perspective. *Am J Ther* 2008; 15: 565–576
31. Shireman TI, Mahnken JD, Phadnis MA *et al*. Effectiveness comparison of cardio-selective to non-selective beta-blockers and their association with mortality and morbidity in end-stage renal disease: a retrospective cohort study. *BMC Cardiovasc Disord* 2016; 16: 60
32. DiNicolantonio JJ, Hackam DG. Carvedilol: a third-generation beta-blocker should be a first-choice β-blocker. *Expert Rev Cardiovasc Ther* 2012; 10: 13–25
33. Van Buren PN, Inrig JK. Mechanisms and treatment of intradialytic hypertension. *Blood Purif* 2016; 41: 188–193
34. Van Buren PN, Toto R, Inrig JK. Interdialytic ambulatory blood pressure in patients with intradialytic hypertension. *Curr Opin Nephrol Hypertens* 2012; 21: 15–23
35. Kanegae K, Hiroshige K, Suda T *et al*. Pharmacokinetics of bisoprolol and its effect on dialysis refractory hypertension. *Int J Artif Organs* 1999; 22: 798–804
36. Kiss I, Farsang C, Rodicio JL. Treatment of hypertension in dialysed patients. *J Hypertens* 2005; 23: 222–226
37. Bakris GL, Fonseca V, Katholi RE *et al*. Metabolic effects of carvedilol vs metoprolol in patients with type 2 diabetes mellitus and hypertension: a randomized controlled trial. *JAMA* 2004; 292: 2227–2236
38. Bakris GL, Hart P, Ritz E. Beta blockers in the management of chronic kidney disease. *Kidney Int* 2006; 70: 1905–1913
39. Roberts MA, Darssan D, Badve SV *et al*. Carvedilol and cardiac biomarkers in dialysis patients: secondary analysis of a randomized controlled trial. *Kidney Blood Press Res* 2017; 42: 1033–1044
40. Hsieh CY, Chen CH, Li CY *et al*. Validating the diagnosis of acute ischemic stroke in a National Health Insurance claims database. *J Formos Med Assoc* 2015; 114: 254–259
41. Lin CC, Lai MS, Syu CY *et al*. Accuracy of diabetes diagnosis in health insurance claims data in Taiwan. *J Formos Med Assoc* 2005; 104: 157–163

A Comparative Study of Carvedilol Versus Metoprolol Initiation and 1-Year Mortality Among Individuals Receiving Maintenance Hemodialysis

Magdalene M. Assimon, M. Alan Brookhart, Jason P. Fine, Gerardo Heiss, J. Bradley Layton, and Jennifer E. Flythe

Background: Carvedilol and metoprolol are the β-blockers most commonly prescribed to US hemodialysis patients, accounting for ∼80% of β-blocker prescriptions. Despite well-established pharmacologic and pharmacokinetic differences between the 2 medications, little is known about their relative safety and efficacy in the hemodialysis population.

Study Design: A retrospective cohort study using a new-user design.

Setting & Participants: Medicare-enrolled hemodialysis patients treated at a large US dialysis organization who initiated carvedilol or metoprolol therapy from January 1, 2007, through December 30, 2012.

Predictor: Carvedilol versus metoprolol initiation.

Outcomes: All-cause mortality, cardiovascular mortality, and intradialytic hypotension (systolic blood pressure decrease ≥ 20 mm Hg during hemodialysis plus intradialytic saline solution administration) during a 1-year follow-up period.

Measurements: Survival models were used to estimate HRs and 95% CIs in mortality analyses. Poisson regression was used to estimate incidence rate ratios (IRRs) and 95% CIs in intradialytic hypotension analyses. Inverse probability of treatment weighting was used to adjust for several demographic, clinical, laboratory, and dialysis treatment covariates in all analyses.

Results: 27,064 individuals receiving maintenance hemodialysis were included: 9,558 (35.3%) carvedilol initiators and 17,506 (64.7%) metoprolol initiators. Carvedilol (vs metoprolol) initiation was associated with greater all-cause (adjusted HR, 1.08; 95% CI, 1.02-1.16) and cardiovascular mortality (adjusted HR, 1.18; 95% CI, 1.08-1.29). In subgroup analyses, similar associations were observed among patients with hypertension, atrial fibrillation, heart failure, and a recent myocardial infarction, the main cardiovascular indications for β-blocker therapy. During follow-up, carvedilol (vs metoprolol) initiators had a higher rate of intradialytic hypotension (adjusted IRR, 1.10; 95% CI, 1.09-1.11).

Limitations: Residual confounding may exist.

Conclusions: Relative to metoprolol initiation, carvedilol initiation was associated with higher 1-year all-cause and cardiovascular mortality. One potential mechanism for these findings may be the increased occurrence of intradialytic hypotension after carvedilol (vs metoprolol) initiation.

Complete author and article information provided before references.

Correspondence to M.M. Assimon (masimon@live.unc.edu)

Am J Kidney Dis. XX(XX): 1-12. Published online Month X, 2017.

doi: 10.1053/j.ajkd.2018.02.350

© 2018 by the National Kidney Foundation, Inc.

Individuals receiving maintenance hemodialysis have cardiovascular mortality rates that exceed those of the general population by 5- to 7-fold.[1] Cardioprotective medications such as β-blockers, among others, are often prescribed to reduce cardiovascular risk. However, clinical trials establishing the cardioprotective nature and safety of β-blockers largely excluded individuals with end-stage renal disease (ESRD).[2,3] Approximately 65% of the US hemodialysis population is treated with a β-blocker.[4] Despite widespread use, surprisingly little is known about the relative safety and efficacy of different β-blockers in hemodialysis patients, a population with special drug dosing considerations.

Within the β-blocker class, individual medications possess different pharmacologic and pharmacokinetic properties. Pharmacologically, β-blockers differ with respect to their β-adrenergic receptor selectivity and vasodilatory capabilities. Kinetically, physiochemical factors, such as molecular size, hydrophilicity, plasma protein binding, and volume of distribution, influence the extent of β-blocker clearance by the hemodialysis procedure (ie, dialyzability). These key differences may plausibly alter the hemodynamic and antiarrhythmic risk-benefit profiles of individual β-blockers in the setting of ESRD.

Observational data suggest that the potential survival benefit conferred by β-blockers may differ across agents. In a Canadian cohort, Weir et al[5] found that the risk of all-cause death was significantly higher among hemodialysis patients treated with high-dialyzability β-blockers (acebutolol, atenolol, and metoprolol tartrate) as compared to patients treated with low-dialyzability β-blockers (bisoprolol and propranolol). However, carvedilol and metoprolol succinate, 2 commonly prescribed β-blockers in the United States,[4] were not considered due to Canadian provincial prescription formulary restrictions. Carvedilol is a nonselective β-blocker with α-blocking effects and is minimally cleared by hemodialysis. Metoprolol (tartrate and succinate) is a cardioselective β-blocker and is

extensively cleared by hemodialysis. The marked pharmacologic and pharmacokinetic heterogeneity between carvedilol and metoprolol may differentially influence clinical outcomes and safety among individuals receiving maintenance hemodialysis and warrants further study.

Although a head-to-head randomized clinical trial would be the ideal approach to investigate the comparative safety and efficacy of carvedilol and metoprolol in the dialysis population, a recent feasibility study suggests that recruitment for such a trial may be challenging.[6] Well-designed pharmacoepidemiologic studies are thus needed to inform clinical decision making. We undertook this study to investigate the association between carvedilol versus metoprolol initiation and 1-year mortality in a cohort of prevalent hemodialysis patients treated at a large US dialysis organization.

Methods

This study was approved by the University of North Carolina at Chapel Hill Institutional Review Board (#15-2651). A waiver of consent was granted due to the study's large size, data anonymity, and retrospective nature.

Data Source

Study data were extracted from the clinical database of a large US dialysis organization and the US Renal Data System (USRDS). Data were linked at the patient level. The dialysis organization operates more than 1,500 outpatient dialysis clinics throughout the nation. Its database captures detailed demographic, clinical, laboratory, and dialysis treatment data. Laboratory data were measured on a biweekly or monthly basis. Hemodialysis treatment parameters were recorded on a treatment-to-treatment basis. The USRDS is a national ESRD surveillance system that includes the Medical Evidence and ESRD Death Notification forms, the Medicare Enrollment database (a repository of Medicare beneficiary enrollment and entitlement data), and Medicare standard analytic files (final action administrative claims data including Medicare parts A, B, and D).

Study Design and Population

We conducted a retrospective cohort study using an active comparator new-user design,[7] the observational analogue to a head-to-head randomized controlled trial, to investigate the association between carvedilol versus metoprolol initiation and 1-year all-cause and cardiovascular mortality (separately) among individuals receiving maintenance hemodialysis. Using a new-user study design to evaluate the comparative safety and/or effectiveness of medications in retrospective investigations helps mitigate biases common to observational studies of prescription drugs, such as selection and immortal time biases.

Figure 1 displays the study design. First, using Medicare Part D claims, we identified dialysis patients treated at the large dialysis organization who initiated oral β-blocker therapy from January 1, 2007, to December 30, 2012, following a 180-day baseline period free of any documented oral β-blocker use (ie, a β-blocker washout period). We then applied the following exclusion criteria: (1) age older than 18 years at the start of the baseline period; (2) dialysis vintage of 90 days or less at the start of the baseline period (to ensure that all potential study patients were eligible for Medicare coverage regardless of their age); (3) lack of continuous Medicare parts A, B, and D coverage during the entire baseline period; (4) receipt of home hemodialysis or peritoneal dialysis during the baseline period; (5) receipt of fewer than 6 center-based hemodialysis treatments in the last 30 days of the baseline period; (6) receipt of hospice care during the baseline period; (7) missing demographic or laboratory data; and (8) initiation of treatment with an oral β-blocker other than carvedilol or metoprolol. The study cohort consisted of prevalent center-based hemodialysis patients who were carvedilol or metoprolol new-users.

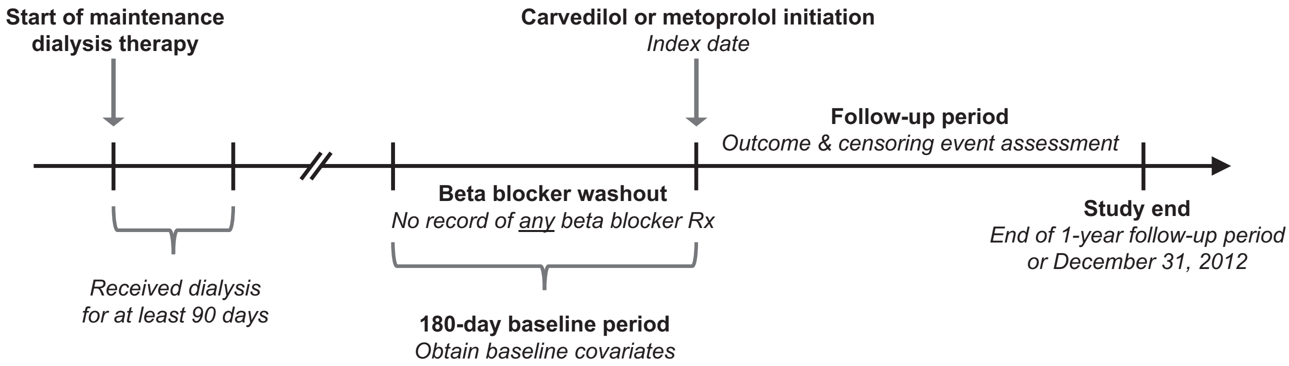

Figure 1. Study design. Carvedilol and metoprolol initiators were defined as hemodialysis patients who had no record of a β-blocker prescription in the previous 180 days (β-blocker washout period). Among these patients, the index date was defined as the date of carvedilol or metoprolol initiation. Baseline covariates were identified in the 180-day period before the index date. Study follow-up began immediately after the index date. To ensure that all potential study patients were eligible for Medicare coverage regardless of their age, individuals needed to have dialysis vintage longer than 90 days at the start of the baseline period. Abbreviation: Rx, prescription.

Study Exposure, Outcomes, and Censoring Events

Exposures of interest were carvedilol and metoprolol initiation. The index date was designated as the date of the first carvedilol or metoprolol prescription after the washout period. Primary study outcomes were 1-year all-cause and cardiovascular mortality (assessed separately). Secondary outcomes were all-cause and cardiovascular hospitalizations (assessed separately) during the 1-year follow-up period. Mortality and hospitalization outcomes were defined using established USRDS definitions (Table S1).[8] Censoring events included kidney transplantation; dialysis modality change; recovery of kidney function; loss of Medicare Part A, B, or D coverage; being lost to follow-up; reaching 1-year of follow-up post–index date; or study end (December 31, 2012).

Baseline Covariate Determination

Baseline covariates included potential confounders and variables known to be strong risk factors for death in the hemodialysis population.[9] Similar to previous pharmacoepidemiologic analyses using USRDS data,[10-13] covariates were identified in the 180 days before the index date and included patient demographics, comorbid conditions, laboratory data, dialysis treatment parameters, and prescription medication use (Table S2). Use of a 180-day baseline period enabled us to maximize cohort generalizability and facilitated capture of patient characteristics that: (1) occurred close to study medication initiation that may have influenced β-blocker prescribing decisions,[14] and (2) are highly predictive of the study outcomes.[15]

Statistical Analysis

All analyses were performed using SAS, version 9.4 (SAS Institute Inc). Baseline characteristics were described across carvedilol and metoprolol initiators as count and percent for categorical variables and mean ± standard deviation for continuous variables. Baseline covariate distributions were compared using standardized differences. A standardized difference > 0.1 represents meaningful imbalance between treatment groups.[16]

In primary analyses, we used an intent-to-treat approach to evaluate the association between carvedilol (vs metoprolol) initiation and 1-year all-cause and cardiovascular mortality. Individuals were followed forward in historical time from the index date to the first occurrence of a study outcome or censoring event. Cox proportional hazards models were used to assess the study β-blocker–all-cause mortality association. Fine and Gray proportional subdistribution hazards models,[17] that treated noncardiovascular death as a competing risk, were used to assess the study β-blocker–cardiovascular mortality association. Both models estimate hazard ratios (HRs) and their 95% confidence intervals (CIs). Robust variance estimation was used in all analyses.[18] Inverse probability of treatment (IPT) weighting was used to control for confounding. We used multivariable logistic regression to calculate the predicted probability (ie, propensity score) of receiving carvedilol (vs metoprolol) as a function of baseline covariates. Propensity scores were used to generate IPT weights.[19,20] We estimated adjusted HRs by applying IPT weights in regression models.

We conducted several sensitivity analyses to assess the robustness of our primary results. First, because the effect of metoprolol (vs carvedilol) on all-cause mortality may differ by metoprolol formulation,[21] we repeated primary analyses and separately compared: (1) carvedilol versus metoprolol tartrate (the immediate-release formulation), and (2) carvedilol versus metoprolol succinate (the controlled/extended-release formulation). Second, we repeated primary analyses using an on-treatment (ie, per-protocol) approach. In these analyses, index β-blocker treatment discontinuation and switching to a nonindex β-blocker during follow-up were considered as additional censoring events. Third, to further minimize the influence of potential confounding by indication (ie, indication bias), we evaluated the association between carvedilol (vs metoprolol) initiation and 1-year mortality among individuals who did not experience a cardiovascular hospitalization during the last 30 days of the baseline period. Fourth, we tested the specificity of our findings by examining the association between carvedilol (vs metoprolol) initiation and hospitalized bowel obstruction, a tracer (ie, negative control) outcome that we did not expect to be influenced by the use of either of the study medications.

In secondary analyses, we evaluated the study β-blocker–mortality associations within clinically relevant subgroups. We assessed the association between carvedilol (vs metoprolol) initiation and 1-year mortality among individuals with hypertension, atrial fibrillation, heart failure, and a recent myocardial infraction, the main cardiovascular indications for β-blocker therapy. In additional analyses, we assessed the associations between carvedilol (vs metoprolol) initiation and the occurrence of hospitalizations during the 1-year follow-up by estimating incidence rate ratios (IRRs) and their 95% CIs using Poisson regression.

We also conducted post hoc analyses to evaluate potential mechanistic explanations for our study findings. We assessed the association between carvedilol (vs metoprolol) initiation and the occurrence of intradialytic hypotension during the 1-year follow-up period by estimating IRRs and their 95% CIs using Poisson regression. Episodes of intradialytic hypotension were identified using 2 different definitions: (1) a systolic blood pressure decrease ≥ 20 mm Hg during hemodialysis plus intradialytic saline solution administration (a guideline-based definition),[22-24] and (2) an intradialytic nadir systolic blood pressure < 90 mm Hg (a definition shown to associate with mortality).[25] We also evaluated study β-blocker–mortality associations among patients with and without a recent history of frequent intradialytic hypotension. Patients were classified as having a recent history of frequent intradialytic hypotension if they experienced

an episode of intradialytic hypotension (defined both ways, separately) in at least 30% of outpatient hemodialysis treatments during the last 30 days of the baseline period.[25]

Results

Study Cohort Characteristics

Figure 2 displays a flow diagram of study cohort selection. A total of 27,064 individuals receiving maintenance hemodialysis were included in the study: 9,558 (35.3%) carvedilol initiators and 17,506 (64.7%) metoprolol initiators. Overall, study patients had an average age of 59.6 ± 14.7 years, 46.7% were women, 42.9% were black, 19.5% were Hispanic, and the most common ESRD cause was diabetes (49.0%). Cardiovascular comorbid conditions were common; 13.9% of the cohort had atrial fibrillation, 29.9% had coronary atherosclerosis, 72.7% had hypertension, 34.6% had heart failure, 6.6% had a recent myocardial infarction, and 21.7% had peripheral arterial disease.

The propensity score distribution of carvedilol and metoprolol initiators exhibited substantial overlap (Fig S1), indicating that the study groups were highly comparable. Patient baseline characteristics stratified by study β-blocker are presented in Table 1. Before IPT weighting, baseline covariates were generally well balanced between treatment groups (standardized differences ≤ 0.1), with a few exceptions (year of index carvedilol or metoprolol initiation, heart failure, and an ESRD cause of diabetes). After IPT weighting, all baseline covariates were well balanced between treatment groups.

Primary Analyses

Under the intent-to-treat paradigm, the study cohort was followed up for a total of 20,863 person-years (7,219 person-years for carvedilol initiators and 13,644 person-years for metoprolol initiators). Average durations of follow-up were 276 days for carvedilol initiators and 285 days for metoprolol initiators. During follow-up, 4,296 all-cause deaths (1,625 in the carvedilol group and 2,671 in the metoprolol group) and 1,943 cardiovascular deaths (782 in the carvedilol group and 1,161 in the metoprolol group) occurred. Figure 3 displays the associations between carvedilol (vs metoprolol) initiation and 1-year all-cause and cardiovascular mortality. Compared with individuals initiating metoprolol treatment, individuals initiating carvedilol treatment had a higher rate of all-cause mortality (225.1 vs 195.8 events/1,000 person-years; adjusted HR, 1.08 [95% CI, 1.02-1.16]) and cardiovascular mortality (108.3

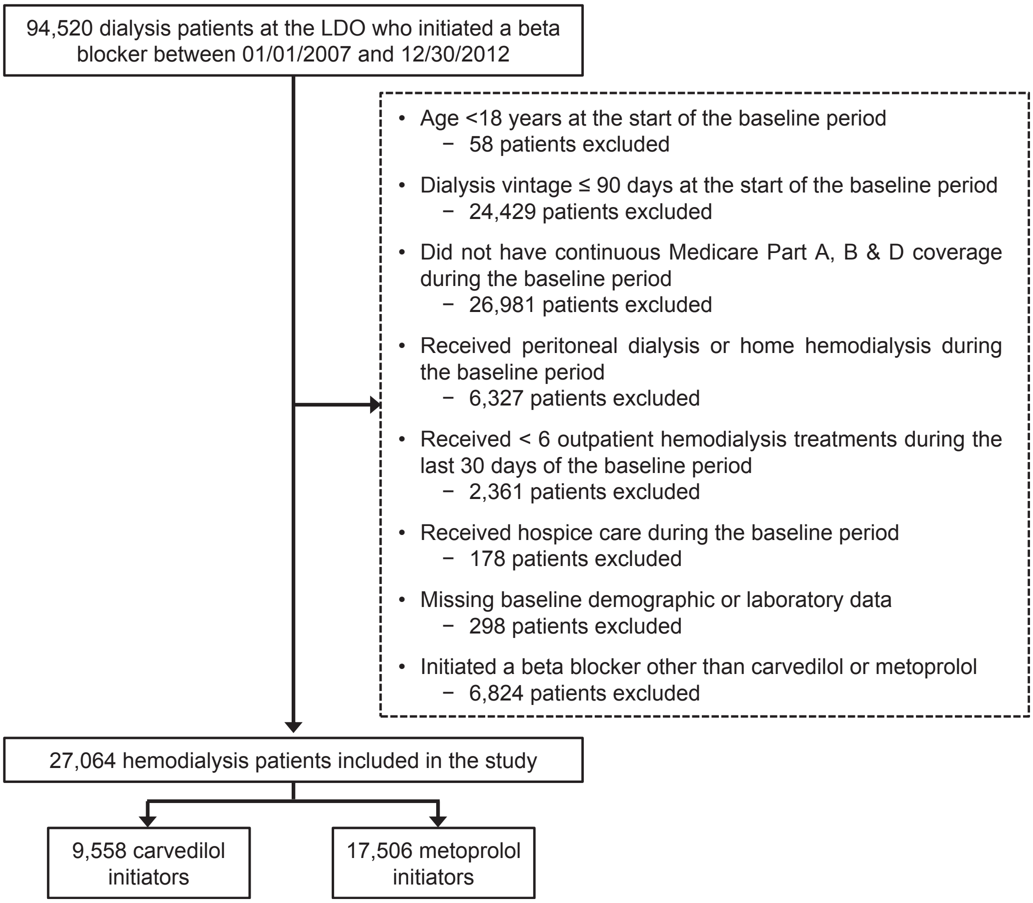

Figure 2. Flow diagram depicts the assembly of the study cohort. Abbreviation: LDO, large dialysis organization.

Table 1. Baseline Characteristics of Study Patients Initiating Carvedilol and Metoprolol

Characteristic	Unweighted			Weighted		
	Carvedilol (n = 9,558)	Metoprolol (n = 17,506)	Std Diff[a]	Carvedilol (n = 9,533)	Metoprolol (n = 17,521)	Std Diff[a]
Age, y	59.8 ±14.4	59.5 ±14.9	0.026	59.8 ± 14.4	59.5 ± 14.9	0.026
Female sex	4,314 (45.1%)	8,316 (47.5%)	0.048	4,444 (46.6%)	8,183 (46.7%)	0.002
Race						
White	4,848 (50.7%)	9,054 (51.7%)	0.020	4,881 (51.2%)	8,991 (51.3%)	0.002
Black	4,186 (43.8%)	7,419 (42.4%)	0.029	4,103 (43.0%)	7,524 (42.9%)	0.002
Other	524 (5.5%)	1,033 (5.9%)	0.018	549 (5.8%)	1,006 (5.7%)	0.001
Hispanic ethnicity	1,925 (20.1%)	3,351 (19.1%)	0.025	1,874 (19.7%)	3,428 (19.6%)	0.002
Low-income subsidy	7,259 (75.9%)	13,524 (77.3%)	0.031	7,328 (76.9%)	13,463 (76.8%)	0.001
Year index β-blocker was prescribed						
2007	1,339 (14.0%)	3,364 (19.2%)	0.140	1,631 (17.1%)	3,034 (17.3%)	0.005
2008	1,385 (14.5%)	3,011 (17.2%)	0.074	1,534 (16.1%)	2,833 (16.2%)	0.002
2009	1,440 (15.1%)	2,561 (14.6%)	0.012	1,406 (14.8%)	2,588 (14.8%)	0.000
2010	1,524 (15.9%)	2,696 (15.4%)	0.015	1,497 (15.7%)	2,736 (15.6%)	0.002
2011	1,804 (18.9%)	2,852 (16.3%)	0.068	1,665 (17.5%)	3,029 (17.3%)	0.005
2012	2,066 (21.6%)	3,022 (17.3%)	0.110	1,801 (18.9%)	3,302 (18.8%)	0.001
Cause of ESRD						
Diabetes	5,027 (52.6%)	8,227 (47.0%)	0.112	4,703 (49.3%)	8,606 (49.1%)	0.004
Hypertension	2,563 (26.8%)	5,051 (28.9%)	0.045	2,686 (28.2%)	4,927 (28.1%)	0.001
Glomerular disease	909 (9.5%)	1,936 (11.1%)	0.051	982 (10.3%)	1,828 (10.4%)	0.004
Other	1,059 (11.1%)	2,292 (13.1%)	0.062	1,163 (12.2%)	2,160 (12.3%)	0.004
Body mass index						
<18.5 kg/m^2	474 (5.0%)	844 (4.8%)	0.006	464 (4.9%)	854 (4.9%)	0.000
18.5-24.9 kg/m^2	3,555 (37.2%)	6,285 (35.9%)	0.027	3,475 (36.5%)	6,371 (36.4%)	0.002
25.0-29.9 kg/m^2	2,761 (28.9%)	4,978 (28.4%)	0.010	2,719 (28.5%)	5,005 (28.6%)	0.001
≥30.0 kg/m^2	2,768 (29.0%)	5,399 (30.8%)	0.041	2,875 (30.2%)	5,292 (30.2%)	0.001
History of prior kidney transplantation	502 (5.3%)	1,204 (6.9%)	0.068	594 (6.2%)	1,103 (6.3%)	0.003
Dialysis vintage						
0.7-0.9 y	595 (6.2%)	935 (5.3%)	0.038	536 (5.6%)	988 (5.6%)	0.001
1.0-1.9 y	2,118 (22.2%)	3,705 (21.2%)	0.024	2,053 (21.5%)	3,778 (21.6%)	0.001
2.0-2.9 y	1,668 (17.5%)	2,778 (15.9%)	0.042	1,556 (16.3%)	2,875 (16.4%)	0.002
≥3.0 y	5,177 (54.2%)	10,088 (57.6%)	0.070	5,388 (56.5%)	9,881 (56.4%)	0.003
CV admission during the last 30 d of baseline	1,801 (18.8%)	2,815 (16.1%)	0.073	1,618 (17.0%)	2,989 (17.1%)	0.002
Atrial fibrillation	1,236 (12.9%)	2,525 (14.4%)	0.043	1,300 (13.6%)	2,426 (13.8%)	0.006
Other arrhythmia	930 (9.7%)	1,630 (9.3%)	0.014	906 (9.5%)	1,657 (9.5%)	0.002
Angina	210 (2.2%)	302 (1.7%)	0.034	182 (1.9%)	334 (1.9%)	0.000
Cancer	312 (3.3%)	661 (3.8%)	0.028	335 (3.5%)	627 (3.6%)	0.003
Conduction disorder	367 (3.8%)	496 (2.8%)	0.056	304 (3.2%)	559 (3.2%)	0.000
COPD/asthma	1,704 (17.8%)	2,795 (16.0%)	0.050	1,601 (16.8%)	2,922 (16.7%)	0.003
Coronary atherosclerosis	3,126 (32.7%)	4,960 (28.3%)	0.095	2,867 (30.1%)	5,251 (30.0%)	0.002
Diabetes	5,473 (57.3%)	9,286 (53.0%)	0.085	5,236 (54.9%)	9,586 (54.7%)	0.004
GI bleed	471 (4.9%)	932 (5.3%)	0.018	503 (5.3%)	911 (5.2%)	0.004
Heart failure	4,107 (43.0%)	5,251 (30.0%)	0.272	3,332 (34.9%)	6,087 (34.7%)	0.004
Hypertension	7,021 (73.5%)	12,652 (72.3%)	0.027	6,960 (73.0%)	12,763 (72.8%)	0.004
Liver disease	421 (4.4%)	783 (4.5%)	0.003	434 (4.6%)	784 (4.5%)	0.004
Myocardial infarction	642 (6.7%)	1,151 (6.6%)	0.006	644 (6.8%)	1,171 (6.7%)	0.003
Peripheral artery disease	2,149 (22.5%)	3,729 (21.3%)	0.029	2,095 (22.0%)	3,820 (21.8%)	0.004
Stroke	975 (10.2%)	1,876 (10.7%)	0.017	1,030 (10.8%)	1,861 (10.6%)	0.006
Valvular disease	904 (9.5%)	1,337 (7.6%)	0.065	795 (8.3%)	1,457 (8.3%)	0.001

(Continued)

Table 1 (Cont'd). Baseline Characteristics of Study Patients Initiating Carvedilol and Metoprolol

	Unweighted			Weighted		
Characteristic	Carvedilol (n = 9,558)	Metoprolol (n = 17,506)	Std Diff[a]	Carvedilol (n = 9,533)	Metoprolol (n = 17,521)	Std Diff[a]
History of treatment nonadherence[b]	594 (6.2%)	1,021 (5.8%)	0.016	581 (6.1%)	1,051 (6.0%)	0.004
Vascular access						
Fistula	5,645 (59.1%)	10,054 (57.4%)	0.033	5,516 (57.9%)	10,150 (57.9%)	0.001
Graft	2,428 (25.4%)	4,451 (25.4%)	0.001	2,448 (25.7%)	4,470 (25.5%)	0.004
Catheter	1,485 (15.5%)	3,001 (17.1%)	0.043	1,570 (16.5%)	2,902 (16.6%)	0.003
Interdialytic weight gain ≥ 3 kg	2,377 (24.9%)	4,196 (24.0%)	0.021	2,310 (24.2%)	4,253 (24.3%)	0.001
Delivered dialysis treatment time < 240 min	7,657 (80.1%)	13,940 (79.6%)	0.012	7,628 (80.0%)	13,989 (79.8%)	0.004
Predialysis systolic BP						
<130 mm Hg	1,384 (14.5%)	2,159 (12.3%)	0.063	1,241 (13.0%)	2,289 (13.1%)	0.001
130-149 mm Hg	2,696 (28.2%)	4,744 (27.1%)	0.025	2,621 (27.5%)	4,808 (27.4%)	0.001
150-169 mm Hg	3,175 (33.2%)	6,084 (34.8%)	0.032	3,253 (34.1%)	5,997 (34.2%)	0.002
≥170 mm Hg	2,303 (24.1%)	4,519 (25.8%)	0.040	2,419 (25.4%)	4,427 (25.3%)	0.002
Recent history of frequent IDH[c]	1,349 (14.1%)	2,363 (13.5%)	0.018	1,321 (13.9%)	2,415 (13.8%)	0.002
Albumin						
≤3.0 g/dL	468 (4.9%)	883 (5.0%)	0.007	483 (5.1%)	877 (5.0%)	0.003
3.1-4.0 g/dL	6,221 (65.1%)	11,057 (63.2%)	0.040	6,092 (63.9%)	11,191 (63.9%)	0.001
>4.0 g/dL	2,869 (30.0%)	5,566 (31.8%)	0.038	2,959 (31.0%)	5,453 (31.1%)	0.002
Calcium						
<8.5 mg/dL	1,338 (14.0%)	2,497 (14.3%)	0.008	1,352 (14.2%)	2,488 (14.2%)	0.001
8.5-10.2 mg/dL	7,756 (81.1%)	14,159 (80.9%)	0.007	7,714 (80.9%)	14,180 (80.9%)	0.000
>10.2 mg/dL	464 (4.9%)	850 (4.9%)	0.000	467 (4.9%)	853 (4.9%)	0.002
Phosphorus						
<3.5 mg/dL	1,088 (11.4%)	1,907 (10.9%)	0.016	1,050 (11.0%)	1,936 (11.0%)	0.001
3.5-5.5 mg/dL	5,224 (54.7%)	9,431 (53.9%)	0.016	5,175 (54.3%)	9,495 (54.2%)	0.002
>5.5 mg/dL	3,246 (34.0%)	6,168 (35.2%)	0.027	3,309 (34.7%)	6,091 (34.8%)	0.001
Potassium						
<4.0 mEq/L	1,064 (11.1%)	1,918 (11.0%)	0.006	1,047 (11.0%)	1,931 (11.0%)	0.001
4.0-6.0 mEq/L	8,152 (85.3%)	14,915 (85.2%)	0.003	8,127 (85.2%)	14,934 (85.2%)	0.000
>6.0 mEq/L	342 (3.6%)	673 (3.8%)	0.014	360 (3.8%)	656 (3.7%)	0.002
Hemoglobin						
<9.5 g/dL	663 (6.9%)	1,166 (6.7%)	0.011	650 (6.8%)	1,185 (6.8%)	0.002
9.5-12.0 mg/dL	6,164 (64.5%)	10,709 (61.2%)	0.069	5,972 (62.6%)	10,942 (62.4%)	0.004
>12.0 mg/dL	2,731 (28.6%)	5,631 (32.2%)	0.078	2,912 (30.5%)	5,394 (30.8%)	0.005
Equilibrated Kt/V < 1.2	2,235 (23.4%)	3,850 (22.0%)	0.033	2,145 (22.5%)	3,944 (22.5%)	0.000
No. of medications in last 30 d of baseline	5.5 ± 3.8	5.5 ± 3.9	0.014	5.5 ± 3.9	5.5 ± 3.9	0.014
α-Blocker	63 (0.7%)	168 (1.0%)	0.034	83 (0.9%)	151 (0.9%)	0.001
ACE inhibitor	2,232 (23.4%)	4,040 (23.1%)	0.006	2,224 (23.3%)	4,070 (23.2%)	0.002
Angiotensin receptor blocker	1,212 (12.7%)	1,848 (10.6%)	0.066	1,103 (11.6%)	2,004 (11.4%)	0.004
Calcium channel blocker	3,060 (32.0%)	5,959 (34.0%)	0.043	3,195 (33.5%)	5,853 (33.4%)	0.002
Central α-agonist	1,272 (13.3%)	2,486 (14.2%)	0.026	1,339 (14.0%)	2,446 (14.0%)	0.003
Diuretic	1,239 (13.0%)	1,845 (10.5%)	0.075	1,095 (11.5%)	2,010 (11.5%)	0.000
Vasodilator	997 (10.4%)	1,916 (10.9%)	0.017	1,030 (10.8%)	1,893 (10.8%)	0.000
Statin	2,578 (27.0%)	4,509 (25.8%)	0.028	2,512 (26.4%)	4,606 (26.3%)	0.001
Other cholesterol medication[d]	394 (4.1%)	717 (4.1%)	0.001	394 (4.1%)	720 (4.1%)	0.001
Digoxin	258 (2.7%)	332 (1.9%)	0.054	205 (2.2%)	382 (2.2%)	0.002
Long-acting nitrate	845 (8.8%)	1,216 (6.9%)	0.070	733 (7.7%)	1,344 (7.7%)	0.001
Antiplatelet medication	1,280 (13.4%)	2,065 (11.8%)	0.048	1,202 (12.6%)	2,187 (12.5%)	0.004
Anticoagulant medication	711 (7.4%)	1,458 (8.3%)	0.033	754 (7.9%)	1,401 (8.0%)	0.003

(Continued)

Table 1 (Cont'd). Baseline Characteristics of Study Patients Initiating Carvedilol and Metoprolol

	Unweighted			Weighted		
Characteristic	Carvedilol (n = 9,558)	Metoprolol (n = 17,506)	Std Diff[a]	Carvedilol (n = 9,533)	Metoprolol (n = 17,521)	Std Diff[a]
Midodrine	192 (2.0%)	350 (2.0%)	0.001	192 (2.0%)	352 (2.0%)	0.000
Use of ≥ 1 potent inhibitor of CYP2D6[e]	2,690 (29.5%)	5,162 (28.1%)	0.030	2,767 (29.0%)	5,090 (29.0%)	0.001

Note: All-covariates were measured during the baseline period before carvedilol or metoprolol initiation. Values are given as number (percent) for categorical variables and as mean ± standard deviation for continuous variables. The weighted cohort is the pseudo-population that was generated by the inverse probability of treatment weighting process.
Abbreviations: ACE, angiotensin-converting enzyme; BP, blood pressure; COPD, chronic obstructive pulmonary disease; CV, cardiovascular; CYP2D6, cytochrome P450 2D6; ESRD, end-stage renal disease; GI, gastrointestinal; IDH, intradialytic hypotension; std diff, standardized difference.
[a]A std diff > 0.1 represents meaningful imbalance between groups.[16]
[b]Claims-based definition of nonadherence included *International Classification of Diseases, Ninth Revision* discharge diagnosis codes V15.81 (personal history of noncompliance with medical treatment, presenting hazards to health) and V45.12 (noncompliance with renal dialysis).
[c]Patients were considered as having a recent history of frequent IDH if they had an intradialytic nadir systolic BP < 90 mm Hg in at least 30% of outpatient hemodialysis treatments during the last 30 days of the baseline period.[25]
[d]Other cholesterol medications included the following nonstatin cholesterol medications: bile acid sequestrants, cholesterol absorption inhibitors, fibrates, and niacin.
[e]Both carvedilol and metoprolol are metabolized by CYP2D6. Concomitant use of medications that are potent inhibitors of CYP2D6 may increase serum concentrations of both carvedilol and metoprolol, putting patients at increased risk for β-blocker–related adverse events such as hypotension. CYP2D6 inhibitors included amiodarone, bupropion, chloroquine, cinacalcet, diphenhydramine, fluoxetine, haloperidol, imatinib, paroxetine, propafenone, propoxyphene, quinidine, terbinafine, and thioridazine.

vs 85.1 events/1,000 person-years; adjusted HR, 1.18 [95% CI, 1.08-1.29]) (Figs 3 and S2).

Secondary Analyses

Secondary analyses assessing associations between carvedilol (vs metoprolol) initiation and mortality among individuals with hypertension, atrial fibrillation, heart failure, or a recent myocardial infarction produced results analogous to primary study findings (Tables 2 and S3).

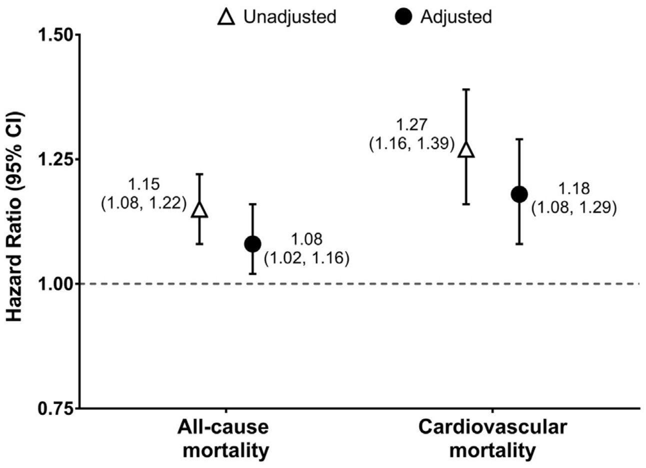

Figure 3. Association between carvedilol versus metoprolol initiation and 1-year mortality: intent-to-treat analysis. An intent-to-treat design was used in all analyses. Cox proportional hazards models were used to estimate the association between carvedilol (vs metoprolol) initiation and 1-year all-cause mortality. Fine and Gray proportional subdistribution hazards models were used to estimate the association between carvedilol (vs metoprolol) initiation and 1-year cardiovascular mortality. In cardiovascular mortality analyses, noncardiovascular death was treated as a competing risk. Inverse probability of treatment weighting was used in adjusted analyses to control for all baseline covariates listed in Table 1. Abbreviation: CI, confidence interval.

In secondary analyses evaluating the associations between study β-blockers and hospitalizations, individuals who initiated carvedilol (vs metoprolol) had similar rates of all-cause hospitalizations (2,383.8 vs 2,270.3 events/1,000 person-years; adjusted IRR, 1.00 [95% CI, 0.97-1.04]) and higher rates of cardiovascular hospitalizations (827.1 vs 726.5 events/1,000 person-years; adjusted IRR, 1.06 [95% CI, 1.01-1.12]) during the 1-year follow-up period.

Sensitivity Analyses

Sensitivity analyses comparing carvedilol initiators with metoprolol tartrate and metoprolol succinate treatment initiators (separately) generated results similar to primary analyses. Treatment with carvedilol (vs metoprolol) was associated with greater 1-year all-cause and cardiovascular mortality, regardless of the comparator metoprolol formulation (Table S4).

In sensitivity analyses using an on-treatment analytic paradigm, the study cohort was followed up for a total of 14,460 person-years (5,127 person-years for carvedilol-treated patients and 9,333 person-years for metoprolol-treated patients). During follow-up, there were 2,941 all-cause deaths (1,117 in the carvedilol group and 1,824 in the metoprolol group) and 1,341 cardiovascular deaths (544 in the carvedilol group and 797 in the metoprolol group). A total of 11,110 individuals discontinued index β-blocker therapy and 1,662 switched to a different β-blocker during follow-up. The average duration of continuous index medication use was 195 days for both carvedilol initiators and metoprolol initiators. Individuals who remained on carvedilol (vs metoprolol) treatment had nominally higher rates of all-cause mortality (217.9 vs 195.4 events/1,000 person-years; adjusted HR, 1.06 [95%, 0.98-1.14]) and higher rates of cardiovascular mortality (106.3 vs 85.4 events/1,000 person-years; adjusted HR, 1.15 [95% CI, 1.03-1.28]).

Sensitivity analyses assessing β-blocker–mortality associations among individuals who did not experience a

Table 2. Association Between Carvedilol Versus Metoprolol Initiation and 1-Year Mortality Among Clinically Relevant Subgroups: Intent-to-Treat Analysis[a]

		1-y All-Cause Mortality[a]		1-y Cardiovascular Mortality[b]	
β-Blocker	n	Rate per 1,000 p-y	Adjusted HR (95% CI)	Rate per 1,000 p-y	Adjusted HR (95% CI)
Patients with hypertension (n = 19,673)					
Metoprolol	12,652	234.7	1.00 (reference)	100.7	1.00 (reference)
Carvedilol	7,021	266.0	1.09 (1.02-1.17)	126.1	1.18 (1.07-1.31)
Patients with atrial fibrillation (n = 3,761)					
Metoprolol	2,525	406.1	1.00 (reference)	174.1	1.00 (reference)
Carvedilol	1,236	458.4	1.08 (0.94-1.23)	215.9	1.12 (0.94-1.35)
Patients with heart failure (n = 9,358)					
Metoprolol	5,251	336.7	1.00 (reference)	144.9	1.00 (reference)
Carvedilol	4,107	335.8	1.02 (0.94-1.11)	157.6	1.09 (0.96-1.23)
Patients with a recent MI (n = 1,793)					
Metoprolol	1,151	395.6	1.00 (reference)	187.1	1.00 (reference)
Carvedilol	642	443.6	1.02 (0.84-1.23)	244.7	1.19 (0.92-1.53)

Note: An intent-to-treat design was used in all analyses. Adjusted analyses controlled for baseline covariates listed in Table 1 using inverse probability of treatment weighting. Subgroups of interest were excluded in the corresponding propensity score models. For example, in subgroup analyses of patients with hypertension, the hypertension covariate was excluded from the propensity score model. Presented patient counts and outcome event rates are based on the unweighted cohort. Abbreviations: CI, confidence interval; HR, hazard ratio; p-y, person-year; MI, myocardial infarction.
[a]Cox proportional hazards models were used to estimate the associations between carvedilol (vs metoprolol) initiation and 1-year all-cause mortality.
[b]Fine and Gray proportional subdistribution hazards models were used to estimate the associations between carvedilol (vs metoprolol) initiation and 1-year cardiovascular mortality. Noncardiovascular death was treated as a competing risk.

cardiovascular hospitalization in the last 30 days of the baseline period produced results analogous to primary study findings. Carvedilol (vs metoprolol) initiation was associated with higher 1-year all-cause and cardiovascular mortality in this patient subgroup (Table S5). In sensitivity analyses evaluating the study β-blocker–tracer outcome association, carvedilol (vs metoprolol) initiation was not associated with the occurrence of hospitalized bowel obstruction (rate of 30.3 vs 28.7 events/1,000 person-years; adjusted HR, 1.02 [95% CI, 0.86-1.20]).

Post Hoc Analyses

The rate of intradialytic hypotension (systolic blood pressure decrease ≥ 20 mm Hg during hemodialysis plus intradialytic saline solution administration) during study follow-up was higher among carvedilol (vs metoprolol) initiators (57.5 vs 55.2 episodes/1,000 person-treatments; adjusted IRR, 1.10 [95% CI, 1.09-1.11]). Similar findings were observed when an episode of intradialytic hypotension was defined as an intradialytic nadir systolic blood pressure < 90 mm Hg (comparing carvedilol with metoprolol initiators: rate of 144.4 vs 136.5 episodes/1,000-person-treatments; adjusted IRR, 1.02 [95% CI, 1.01-1.03]). In additional post hoc analyses, all-cause and cardiovascular mortality associations were higher among individuals with versus without a recent history of frequent intradialytic hypotension (Fig 4; Table S6).

Discussion

This observational study evaluated the comparative mortality risk of carvedilol and metoprolol initiation among individuals receiving maintenance hemodialysis. We found evidence that carvedilol (vs metoprolol) initiation was associated with greater 1-year all-cause and cardiovascular mortality. The associations were consistent within clinically relevant subgroups and robust across sensitivity analyses. We also found that carvedilol initiators experienced higher rates of intradialytic hypotension during follow-up compared with metoprolol initiators. In addition, the observed study β-blocker–mortality associations were more pronounced among individuals with versus without a recent history of frequent intradialytic hypotension.

To date, there have been no randomized clinical trials comparing the efficacy and safety of individual β-blockers in the dialysis population. Prior β-blocker clinical trials were either placebo controlled[6,26] or compared β-blockers with other antihypertensive medication classes (eg, angiotensin-converting enzyme inhibitors).[27] Existing observational investigations of β-blockers have predominantly focused on comparing β-blocker users with nonusers,[28-34] and only 2 observational studies have considered head-to-head β-blocker comparisons. Weir et al[5] assessed the association between β-blocker dialyzability and 180-day mortality in a cohort of 6,588 elderly Canadian hemodialysis patients. Initiation of a highly versus a minimally dialyzable β-blocker was associated with higher all-cause death. This study provided initial evidence that β-blocker heterogeneity may differentially affect clinical outcomes in the hemodialysis population; however, carvedilol (a minimally dialyzable β-blocker) and metoprolol succinate (a highly dialyzable β-blocker) were not considered. In the US, carvedilol and metoprolol succinate account for 50% of all β-blocker prescriptions.

In a second epidemiologic study, Shireman et al[35] evaluated the association between β-blocker selectivity

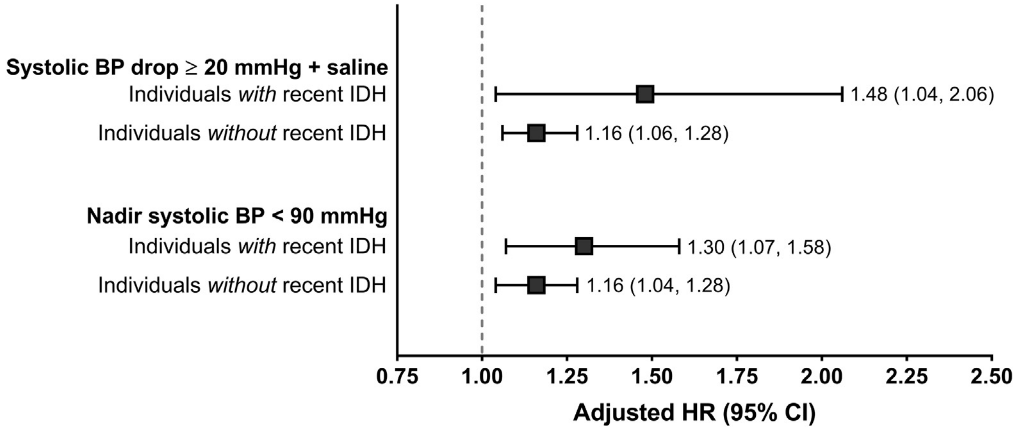

Figure 4. Association between carvedilol versus metoprolol initiation and 1-year cardiovascular mortality among individuals with and without a recent history of intradialytic hypotension (IDH): intent-to-treat analysis. An intent-to-treat design was used in all analyses. Fine and Gray proportional subdistribution hazards models were used to estimate the association between carvedilol (vs metoprolol) initiation and 1-year cardiovascular mortality. In these analyses, noncardiovascular death was treated as a competing risk. Inverse probability of treatment weighting was used in adjusted analyses to control for all baseline covariates listed in Table 1. Abbreviations: BP, blood pressure; CI, confidence interval; HR, hazard ratio; IDH, intradialytic hypotension.

and mortality in a cohort of 4,398 incident US hemodialysis and peritoneal dialysis patients with dual Medicare/Medicaid coverage and hypertension. Initiation of a cardioselective β-blocker (atenolol and metoprolol) versus a nonselective β-blocker (carvedilol and labetalol) was associated with greater survival. However, the relative contributions of carvedilol and metoprolol to the observed association are unclear, and this investigation relied on data from 2000 to 2005. In the last decade, carvedilol use has increased,[4,36] rendering a contemporary analysis important. International guideline bodies have called for additional comparative effectiveness research on putative cardioprotective drugs such as β-blockers in the hemodialysis population.[37]

To begin to address this evidence gap, we performed a head-to-head comparison of the 2 most commonly prescribed β-blockers in the United States; carvedilol and metoprolol. We found that carvedilol (vs metoprolol) initiation was associated with higher 1-year all-cause and cardiovascular mortality. Results were consistent among individuals with hypertension, atrial fibrillation, heart failure, and a recent myocardial infarction. Furthermore, the observed study β-blocker–mortality associations were robust across sensitivity analyses comparing carvedilol to immediate-release metoprolol tartrate and extended/controlled-release metoprolol succinate (separately). In post hoc analyses, we found that the association between carvedilol (vs metoprolol) initiation and mortality was more potent among individuals with a recent history of frequent intradialytic hypotension. In addition, the occurrence of intradialytic hypotension (defined 2 ways) was more common after carvedilol (vs metoprolol) initiation. Given that recurrent intradialytic hypotension is associated with increased morbidity and mortality in the hemodialysis population,[25,38-40] the results from our post hoc analyses support the notion that hemodynamic instability may play a mechanistic role in the observed association between carvedilol (vs metoprolol) initiation and greater mortality.

Pharmacologic and kinetic differences between carvedilol and metoprolol may plausibly explain the observed differences in mortality and intradialytic hypotension. First, the extent to which a β-blocker is removed from circulation by hemodialysis may affect intradialytic blood pressure. Carvedilol is minimally dialyzed, and metoprolol is highly dialyzed. As a result, carvedilol's antihypertensive effects are likely maintained over the course of dialysis, whereas metoprolol's antihypertensive effects may be diminished as serum drug concentrations decrease during treatment. Second, carvedilol and metoprolol differ with respect to their β-adrenergic receptor selectivity and vasodilatory capabilities. Carvedilol is a nonselective β-blocker (a β_1- and β_2-adrenergic receptor antagonist) with additional α-blocking activity (an α_1-adrenergic receptor antagonist). In contrast, metoprolol is a cardioselective β-blocker with high β_1-adrenergic receptor affinity. Both medications reduce heart rate and cardiac contractility, but due to its α-blocking effects, carvedilol is also a vasodilator. It is plausible that carvedilol-induced α-blockade may blunt compensatory sympathetic nervous system–mediated peripheral vasoconstriction during ultrafiltration, increasing the risk for intradialytic hemodynamic instability. These proposed clinical mechanisms likely act in concert in carvedilol-treated patients.

Ultimately, randomized controlled clinical trials are needed to definitively determine the relative safety and efficacy of carvedilol and metoprolol in the hemodialysis population. However, in the interim, our results suggest that the potential adverse hemodynamic effects of carvedilol (vs metoprolol) require consideration when

prescribing β-blockers to hemodialysis patients, particularly among individuals with a history of intradialytic hemodynamic instability. For example, it may be reasonable to: (1) consider metoprolol over carvedilol among individuals at higher risk for intradialytic hypotension, or (2) recommend that patients at higher risk for intradialytic hypotension withhold carvedilol doses before hemodialysis treatments to minimize potential intradialytic hypotensive effects. However, such decisions must be made carefully on an individual basis with consideration of comorbid cardiovascular conditions, historical blood pressure patterns, and concomitant antihypertensive medication use and dosing.

Our study has several strengths. First, we used a modern pharmacoepidemiologic study design to evaluate the comparative 1-year mortality risks associated with carvedilol and metoprolol initiation. To minimize the influence of bias due to confounding by indication or disease severity, we selected study medications with similar indications and therapeutic roles.[41] Notably, the carvedilol and metoprolol initiators were highly comparable, and all baseline covariate imbalances between treatment groups were diminished after IPT weighting. Additionally, we chose to study the 2 most commonly prescribed β-blockers to closely mirror a real-world clinical practice decision.[41] Second, unlike previous claims-based studies, we used a linked data set with detailed clinical data that enabled us to account for many important biochemical indexes and dialysis treatment parameters in our analyses. Finally, we performed multiple sensitivity analyses to test the robustness of our findings.

Our results should be considered within the context of study limitations. Because our study was observational, there may be residual confounding. However, we controlled for variables including albumin concentrations, phosphorus concentrations, and a history of nonadherence to treatment to minimize confounding from difficult-to-measure factors such as ambient health status. Reassuringly, carvedilol (vs metoprolol) initiation was not associated with the occurrence of the tracer outcome, hospitalized bowel obstruction. Second, although our linked data source contained detailed administrative and clinical data, information for some potentially important factors, such as the timing of medication dosing, subspecialty of the index β-blocker prescriber, and cardiac status (eg, ejection fraction and left ventricular hypertrophy) were not available. In particular, it is possible that a clinician's decision to prescribe carvedilol over metoprolol was influenced by left ventricular hypertrophy severity or other markers of cardiac function. As such, it is possible that residual confounding by indication (ie, indication bias)[41] may have influenced our results. Third, comorbid condition designations were based upon *International Classification of Diseases, Ninth Revision* diagnosis codes. Administrative claims data are generated for reimbursement and billing purposes. These data may not always reflect clinical subtleties and may not capture all patient characteristics, potentially affecting the accuracy of claims-identified comorbid conditions. For example, only a limited number of discharge diagnoses can be coded for each billable health care encounter, possibly reducing comorbid condition ascertainment. In addition, comorbid conditions not requiring a health care encounter during the 180-day baseline period may have been missed. Reassuringly, our approach facilitated capture of the most severe conditions and thus strongest potential confounders.[15,42] Fourth, our study population was composed of prevalent patients with ESRD receiving in-center hemodialysis. Our results may not be generalizable to excluded populations such as incident hemodialysis, home hemodialysis, or peritoneal dialysis patients. Understanding the relative risk-benefit profiles of carvedilol and metoprolol in these excluded patient populations is an area for future inquiry. Finally, our study evaluated a cohort of US hemodialysis patients. Our results may not apply to other countries that have national or regional prescription formularies which limit metoprolol and/or carvedilol prescribing.

In conclusion, we observed that carvedilol (vs metoprolol) initiation was associated with higher 1-year all-cause and cardiovascular mortality in a cohort of prevalent US hemodialysis patients. Data from our post hoc analyses suggest that one potential mechanism for the observed mortality associations may be an increased rate of intradialytic hypotension after carvedilol (vs metoprolol) initiation. Given the unique pharmacokinetic and hemodynamic considerations in the ESRD population, additional study of the efficacy and safety of β-blockers, as well as other cardioprotective medications with antihypertensive properties, is needed.

Supplementary Material

Figure S1: Propensity score distribution of patients treated with carvedilol and metoprolol.

Figure S2: The 1-year cumulative incidence of all-cause and CV mortality among carvedilol and metoprolol initiators: intent-to-treat analysis.

Table S1: Outcome definitions.

Table S2: Baseline covariate definitions.

Table S3: Association between carvedilol versus metoprolol initiation and 1-year mortality among clinically relevant subgroups: intent-to-treat analysis.

Table S4: Association between the initiation of carvedilol versus the initiation of the different metoprolol formulations and 1-year mortality: intent-to-treat analysis.

Table S5: Association between carvedilol versus metoprolol initiation and 1-year mortality among individuals who did not have a CV hospitalization during the last 30 days of the baseline period: intent-to-treat analysis.

Table S6: Association between carvedilol versus metoprolol initiation and 1-year mortality among individuals with and without a recent history of frequent IDH: intent-to-treat analysis.

Article Information

Authors' Full Names and Academic Degrees: Magdalene M. Assimon, PharmD, PhD, M. Alan Brookhart, PhD, Jason P. Fine, ScD, Gerardo Heiss, MD, PhD, J. Bradley Layton, PhD, and Jennifer E. Flythe, MD, MPH.

Authors' Affiliations: University of North Carolina Kidney Center, Division of Nephrology and Hypertension, Department of Medicine, UNC School of Medicine (MMA, JEF); Departments of Epidemiology (MMA, MAB, GH, JBL) and Biostatistics (JPF), UNC Gillings School of Global Public Health, Chapel Hill; RTI Health Solutions, Research Triangle Park (JBL); and Cecil G. Sheps Center for Health Services Research, University of North Carolina, Chapel Hill, NC (JEF).

Address for Correspondence: Magdalene M. Assimon, PharmD, PhD, University of North Carolina Kidney Center, 7024 Burnett-Womack CB #7155, Chapel Hill, NC 27599-7155. E-mail: masimon@live.unc.edu

Authors' Contributions: Research idea and study design: MMA, MAB, JEF; data acquisition: MMA, MAB, JEF; data analysis/interpretation: MMA, MAB, JPF, GH, JBL, JEF; statistical analysis: MMA; and supervision or mentorship: MAB, JEF. Each author contributed important intellectual content during manuscript drafting or revision and accepts accountability for the overall work by ensuring that questions pertaining to the accuracy or integrity of any portion of the work are appropriately investigated and resolved.

Support: Dr Assimon was supported by grant F32 DK109561, and Dr Flythe, by grant K23 DK109401, both awarded by the National Institute of Diabetes and Digestive and Kidney Diseases of the National Institutes of Health. The funders of this study had no role in study design; collection, analysis, or interpretation of data; writing the manuscript; or the decision to submit the report for publication.

Financial Disclosure: Drs Assimon and Flythe have received investigator-initiated research funding from the Renal Research Institute, a subsidiary of Fresenius Medical Care, North America. Dr Brookhart has received research support from Amgen and AstraZeneca; has served as a scientific advisor for Merck, Amgen, Genentech, and RxAnte; and owns equity in NoviSci, LLC, a data sciences company. Dr Layton was formerly an employee of University of North Carolina (UNC), where he received salary support from the Center for Pharmacoepidemiology of the UNC Department of Epidemiology (center member companies included GlaxoSmithKline, Merck, and UCB Biosciences) and is currently an employee of RTI International, an independent research organization that does work for government and pharmaceutical companies. Dr Flythe has received speaking honoraria from Dialysis Clinic Inc, Renal Ventures, American Renal Associates, American Society of Nephrology, Baxter, National Kidney Foundation, and multiple universities. The other authors declare that they have no relevant financial interests.

Disclaimer: Some of the data reported here have been supplied by DaVita Clinical Research. DaVita Clinical Research had no role in the design or implementation of this study or in the decision to publish. Additionally, some of the data reported here have been provided by the USRDS. The interpretation and reporting of these data are the responsibility of the authors and in no way should be seen as official policy or interpretation of the US government.

Peer Review: Received September 22, 2017. Evaluated by 3 external peer reviewers, with direct editorial input from a Statistics/Methods Editor, an Associate Editor, and the Editor-in-Chief. Accepted in revised form February 4, 2018.

References

1. Saran R, Robinson B, Abbott KC, et al. US Renal Data System 2016 annual data report: epidemiology of kidney disease in the United States. *Am J Kidney Dis*. 2017;69(3)(suppl 1):A7-A8.
2. Coca SG, Krumholz HM, Garg AX, Parikh CR. Underrepresentation of renal disease in randomized controlled trials of cardiovascular disease. *JAMA*. 2006;296(11):1377-1384.
3. Konstantinidis I, Nadkarni GN, Yacoub R, et al. Representation of patients with kidney disease in trials of cardiovascular interventions: an updated systematic review. *JAMA Intern Med*. 2016;176(1):121-124.
4. St Peter WL, Sozio SM, Shafi T, et al. Patterns in blood pressure medication use in US incident dialysis patients over the first 6 months. *BMC Nephrol*. 2013;14:249.
5. Weir MA, Dixon SN, Fleet JL, et al. β-Blocker dialyzability and mortality in older patients receiving hemodialysis. *J Am Soc Nephrol*. 2015;26(4):987-996.
6. Roberts MA, Pilmore HL, Ierino FL, et al. The beta-Blocker to Lower Cardiovascular Dialysis Events (BLOCADE) feasibility study: a randomized controlled trial. *Am J Kidney Dis*. 2016;67(6):902-911.
7. Ray WA. Evaluating medication effects outside of clinical trials: new-user designs. *Am J Epidemiol*. 2003;158(9):915-920.
8. United States Renal Data System. ESRD Analytic Methods. https://www.usrds.org/2016/view/v2_00_appx.aspx. Accessed July 11, 2017.
9. Brookhart MA, Schneeweiss S, Rothman KJ, Glynn RJ, Avorn J, Sturmer T. Variable selection for propensity score models. *Am J Epidemiol*. 2006;163(12):1149-1156.
10. Brookhart MA, Freburger JK, Ellis AR, Wang L, Winkelmayer WC, Kshirsagar AV. Infection risk with bolus versus maintenance iron supplementation in hemodialysis patients. *J Am Soc Nephrol*. 2013;24(7):1151-1158.
11. Kshirsagar AV, Freburger JK, Ellis AR, Wang L, Winkelmayer WC, Brookhart MA. The comparative short-term effectiveness of iron dosing and formulations in US hemodialysis patients. *Am J Med*. 2013;126(6):541.e1-541.e14.
12. Yusuf AA, Weinhandl ED, St Peter WL. Comparative effectiveness of calcium acetate and sevelamer on clinical outcomes in elderly hemodialysis patients enrolled in Medicare Part D. *Am J Kidney Dis*. 2014;64(1):95-103.
13. Weinhandl ED, Nieman KM, Gilbertson DT, Collins AJ. Hospitalization in daily home hemodialysis and matched thrice-weekly in-center hemodialysis patients. *Am J Kidney Dis*. 2015;65(1):98-108.
14. Brookhart MA. Counterpoint: the treatment decision design. *Am J Epidemiol*. 2015;182(10):840-845.
15. Gilbertson DT, Bradbury BD, Wetmore JB, et al. Controlling confounding of treatment effects in administrative data in the presence of time-varying baseline confounders. *Pharmacoepidemiol Drug Saf*. 2016;25(3):269-277.
16. Austin PC. Using the standardized difference to compare the prevalence of a binary variable between two groups in observational research. *Commun Stat Simul Comput*. 2009;38(6):1228-1234.
17. Fine JP, Gray RJ. A proportional hazards model for the subdistribution of a competing risk. *J Am Stat Assoc*. 1999;94(446):496-509.
18. Austin PC. Variance estimation when using inverse probability of treatment weighting (IPTW) with survival analysis. *Stat Med*. 2016;35(30):5642-5655.
19. Cole SR, Hernan MA. Constructing inverse probability weights for marginal structural models. *Am J Epidemiol*. 2008;168(6):656-664.
20. Brookhart MA, Wyss R, Layton JB, Sturmer T. Propensity score methods for confounding control in nonexperimental research. *Circ Cardiovasc Qual Outcomes*. 2013;6(5):604-611.
21. Briasoulis A, Palla M, Afonso L. Meta-analysis of the effects of carvedilol versus metoprolol on all-cause mortality and hospitalizations in patients with heart failure. *Am J Cardiol*. 2015;115(8):1111-1115.

22. K/DOQI Workgroup. K/DOQI clinical practice guidelines for cardiovascular disease in dialysis patients. *Am J Kidney Dis.* 2005;45(4)(suppl 3):S1-S153.
23. Kooman J, Basci A, Pizzarelli F, et al. EBPG guideline on haemodynamic instability. *Nephrol Dial Transplant.* 2007;22(suppl 2):ii22-ii44.
24. Mactier R, Hoenich N, Breen C. UK Renal Association clinical practice guidelines: haemodialysis. http://www.renal.org/guidelines/modules/haemodialysis#sthash.eBdbSrRk.dpbs, 2009. Accessed December 23, 2017.
25. Flythe JE, Xue H, Lynch KE, Curhan GC, Brunelli SM. Association of mortality risk with various definitions of intradialytic hypotension. *J Am Soc Nephrol.* 2015;26(3):724-734.
26. Cice G, Ferrara L, D'Andrea A, et al. Carvedilol increases two-year survival in dialysis patients with dilated cardiomyopathy: a prospective, placebo-controlled trial. *J Am Coll Cardiol.* 2003;41(9):1438-1444.
27. Agarwal R, Sinha AD, Pappas MK, Abraham TN, Tegegne GG. Hypertension in hemodialysis patients treated with atenolol or lisinopril: a randomized controlled trial. *Nephrol Dial Transplant.* 2014;29(3):672-681.
28. Foley RN, Herzog CA, Collins AJ; United States Renal Data System. Blood pressure and long-term mortality in United States hemodialysis patients: USRDS Waves 3 and 4 Study. *Kidney Int.* 2002;62(5):1784-1790.
29. Griffith TF, Chua BS, Allen AS, Klassen PS, Reddan DN, Szczech LA. Characteristics of treated hypertension in incident hemodialysis and peritoneal dialysis patients. *Am J Kidney Dis.* 2003;42(6):1260-1269.
30. Abbott KC, Trespalacios FC, Agodoa LY, Taylor AJ, Bakris GL. beta-Blocker use in long-term dialysis patients: association with hospitalized heart failure and mortality. *Arch Intern Med.* 2004;164(22):2465-2471.
31. Ishani A, Herzog CA, Collins AJ, Foley RN. Cardiac medications and their association with cardiovascular events in incident dialysis patients: cause or effect? *Kidney Int.* 2004;65(3):1017-1025.
32. Nakao K, Makino H, Morita S, et al. Beta-blocker prescription and outcomes in hemodialysis patients from the Japan Dialysis Outcomes and Practice Patterns Study. *Nephron Clin Pract.* 2009;113(3):c132-c139.
33. Tangri N, Shastri S, Tighiouart H, et al. beta-Blockers for prevention of sudden cardiac death in patients on hemodialysis: a propensity score analysis of the HEMO Study. *Am J Kidney Dis.* 2011;58(6):939-945.
34. Kitchlu A, Clemens K, Gomes T, et al. Beta-blockers and cardiovascular outcomes in dialysis patients: a cohort study in Ontario, Canada. *Nephrol Dial Transplant.* 2012;27(4):1591-1598.
35. Shireman TI, Mahnken JD, Phadnis MA, Ellerbeck EF. Effectiveness comparison of cardio-selective to non-selective beta-blockers and their association with mortality and morbidity in end-stage renal disease: a retrospective cohort study. *BMC Cardiovasc Disord.* 2016;16:60.
36. Wetmore JB, Mahnken JD, Mukhopadhyay P, et al. Geographic variation in cardioprotective antihypertensive medication usage in dialysis patients. *Am J Kidney Dis.* 2011;58(1):73-83.
37. Levin NW, Kotanko P, Eckardt KU, et al. Blood pressure in chronic kidney disease stage 5D-report from a Kidney Disease: Improving Global Outcomes controversies conference. *Kidney Int.* 2010;77(4):273-284.
38. Stefansson BV, Brunelli SM, Cabrera C, et al. Intradialytic hypotension and risk of cardiovascular disease. *Clin J Am Soc Nephrol.* 2014;9(12):2124-2132.
39. Chou JA, Streja E, Nguyen DV, et al. Intradialytic hypotension, blood pressure changes and mortality risk in incident hemodialysis patients. *Nephrol Dial Transplant.* 2017;33(1):149-159.
40. Chang TI, Paik J, Greene T, et al. Intradialytic hypotension and vascular access thrombosis. *J Am Soc Nephrol.* 2011;22(8):1526-1533.
41. Velentgas P, Dreyer NA, Nourjah P, Smith SR, Torchia MM, eds. *Developing a Protocol for Observational Comparative Effectiveness Research: A User's Guide.* Rockville, MD: Agency for Healthcare Research and Quality (US); 2013.
42. Goldstein BA, Pencina MJ, Montez-Rath ME, Winkelmayer WC. Predicting mortality over different time horizons: which data elements are needed? *J Am Med Inform Assoc.* 2017;24(1):176-181.

Shireman et al. BMC Cardiovascular Disorders (2016) 16:60
DOI 10.1186/s12872-016-0233-3

BMC Cardiovascular Disorders

RESEARCH ARTICLE Open Access

Effectiveness comparison of cardio-selective to non-selective β-blockers and their association with mortality and morbidity in end-stage renal disease: a retrospective cohort study

Theresa I. Shireman[1*], Jonathan D. Mahnken[2], Milind A. Phadnis[2] and Edward F. Ellerbeck[3,4]

Abstract

Background: Within-class comparative effectiveness studies of β-blockers have not been performed in the chronic dialysis setting. With widespread cardiac disease in these patients and potential mechanistic differences within the class, we examined whether mortality and morbidity outcomes varied between cardio-selective and non-selective β-blockers.

Methods: Retrospective observational study of within class β-blocker exposure among a national cohort of new chronic dialysis patients (*N* = 52,922) with hypertension and dual eligibility (Medicare-Medicaid). New β-blocker users were classified according to their exclusive use of one of the subclasses. Outcomes were all-cause mortality (ACM) and cardiovascular morbidity and mortality (CVMM). The associations of cardio-selective and non-selective agents on outcomes were adjusted for baseline characteristics using Cox proportional hazards.

Results: There were 4938 new β-blocker users included in the ACM model and 4537 in the CVMM model: 77 % on cardio-selective β-blockers. Exposure to cardio-selective and non-selective agents during the follow-up period was comparable, as measured by proportion of days covered (0.56 vs. 0.53 in the ACM model; 0.56 vs 0.54 in the CVMM model). Use of cardio-selective β-blockers was associated with lower risk for mortality (AHR = 0.84; 99 % CI = 0.72–0.97, $p = 0.0026$) and lower risk for CVMM events (AHR = 0.86; 99 % CI = 0.75–0.99, $p = 0.0042$).

Conclusion: Among new β-blockers users on chronic dialysis, cardio-selective agents were associated with a statistically significant 16 % reduction in mortality and 14 % in cardiovascular morbidity and mortality relative to non-selective β-blocker users. A randomized clinical trial would be appropriate to more definitively answer whether cardio-selective β-blockers are superior to non-selective β-blockers in the setting of chronic dialysis.

Keywords: Dialysis, End stage renal disease, Hypertension, Mortality, β-blockers, Comparative effectiveness

* Correspondence: theresa_shireman@brown.edu
[1]Health Services Policy & Practice and the Center for Gerontology & Health Care Research, Brown University School of Public Health, 121 South Main St, Box-G-S121-6, Providence, RI 02912, USA
Full list of author information is available at the end of the article

© 2016 Shireman et al. **Open Access** This article is distributed under the terms of the Creative Commons Attribution 4.0 International License (http://creativecommons.org/licenses/by/4.0/), which permits unrestricted use, distribution, and reproduction in any medium, provided you give appropriate credit to the original author(s) and the source, provide a link to the Creative Commons license, and indicate if changes were made. The Creative Commons Public Domain Dedication waiver (http://creativecommons.org/publicdomain/zero/1.0/) applies to the data made available in this article, unless otherwise stated.

Background

Given their high rates of hypertension and cardiovascular disease (CVD) [1], patients with end-stage renal disease (ESRD) on chronic dialysis often are prescribed medications [2, 3] with cardioprotective properties [4–8]. In the general population, β-adrenergic blocking agents (hereafter referred to as β-blockers) are recommended as first and second line agents across a number of cardiac conditions because of their ability to reduce CVD events in at-risk individuals [9–11]. Risk reduction estimates from meta-analyses and systematic reviews of β-blockers range from a 16 % reduction in mortality in patients with diabetes to a 35 % reduction in mortality primarily from arrhythmia-associated sudden death [12–14].

The evidence base for the use of β-blockers in patients on chronic dialysis originates primarily from observational studies [15–21] and a few randomized trials [22–24], which generally reaffirm that these agents are associated with a therapeutic benefit. For example, using combined data from Dialysis Morbidity & Mortality Studies Waves 3 and 4, Foley and colleagues reported that β-blockers were associated with a 16 % relative risk reduction in all-cause mortality [15]. We also recently reported a significant reduction in all-cause mortality and cardiovascular endpoints associated with β-blockers in a propensity-adjusted modeling of time-dependent exposure [25, 26].

However, when making a choice to prescribe a specific cardioprotective medication for hypertension in the setting of chronic dialysis, providers have to make clinically-relevant selections from a given drug class without having the benefit of a clear evidence base. In the case of β-blockers, subclass distinctions in pharmacological properties, such as degree of β-1 or cardiac selectivity, α-blockade, route of elimination, lipophilicity, and dialyzability, become relevant; such a choice might be further influenced by presence of heart failure. For example, atenolol and metoprolol have high β-1 selectivity and low-to-moderate lipophilicity, making them readily dialyzable and placing them in distinction to carvedilol and labetalol. On the other hand, while theoretically carvedilol may offer advantages in hypertensive and/or heart failure patients [27, 28], observational studies have reported no significant differences in HF readmissions between agents in these subclasses of β-blockers [29, 30].

Within-class comparative effectiveness studies of β-blockers have not been performed in the chronic dialysis setting. In the absence of clinical trial data, well-designed observational studies can provide important preliminary data on the relative effectiveness of different medications, particularly in a patient population widely excluded from trials. The goal of the present study was to compare mortality and cardiovascular event outcomes across two major subclasses of β-blockers, focusing on cardiac selectivity distinctions between agents. To investigate this, we analyzed linked data from the United States Renal Data System (USRDS) with Medicaid pharmacy claims [2, 31] in a large cohort of incident dialysis patients who were newly initiating a β-blocker.

Methods

Study design and data sources

We performed a retrospective cohort analysis of incident, Medicare and Medicaid (dually eligible) chronic dialysis patients, quantifying their exposure to cardioselective and non-selective β-blockers and assessing their outcomes over six years (2000–2005) [2, 31]. We used the dually eligible population because Medicare did not cover prescription medications during this time period. In addition, even with the implementation of drug coverage through Medicare Part D in 2006, medication exposure can still only be fully studied in the low-income subsidy patients (dually eligible), as many of these medications are filled through $4 prescriptions and claims are incomplete. Outcomes assessed were all-cause mortality and a combined outcome that included cardiovascular mortality and morbidity. The comparative effectiveness analyses were performed on new users of β-blockers as described below.

Data for these analyses were assembled from the USRDS and Medicaid (Centers for Medicare & Medicaid Services or CMS). From the USRDS, we obtained standard patient records that included information on demographics, comorbidities, functional status, and dialysis modality (from the Medical Evidence Form, or CMS 2728) recorded at the time of dialysis commencement. The USRDS also incorporated Medicare paid inpatient and outpatient medical claims, a federally-funded program for which the vast majority of adults with end stage renal disease are enrolled [1, 32]. We used Medicaid prescription drug claims to identify β-blocker exposure. Medicaid is a joint federal-state program designed to provide health care benefits to low-income persons: in the case of the dually eligible, Medicaid was the source of prescription drug coverage during the time period. These sources were linked using previously described methodology [31, 33] to permit identification of dually eligible dialysis patients in 2000–05.

Cohort creation

We created a cohort consisting of hypertensive individuals who were new users of β-blockers. This included people who had at least one prescription for a β-blocker during the follow-up period, but no use during the first 90-day run-in period as is described below in greater detail. To assure complete observability of the cohort, we employed several criteria as have also been described

elsewhere [31]. First, we limited the cohort to persons enrolled in a single state's Medicaid fee-for-service program. Persons with coverage through the Veterans Administration and those who had previously been transplanted and returning to chronic dialysis were excluded. Persons who received a transplant, died, or were not continuously eligible for Medicare and Medicaid during the first 90 days on dialysis were excluded. Additionally, persons who did not fill any prescriptions during the first 90 days were excluded (this lack of prescriptions was thought to reflect the Medicaid's spend-down requirements). Ohio residents were excluded since their claims do not include the days supplied of medication. We also excluded persons who were institutionalized during their entire follow-up period, were missing multiple data fields from their dialysis initiation Medical Evidence form (CMS 2728), and/or did not have hypertension documented on CMS 2728. Finally, we selected individuals who received at least one beta-blocker during their follow-up period.

The observation window began at the date the first β-blocker prescription was dispensed. Subjects were then followed until they incurred a first outcome event (death or cardiovascular event). They were censored when they lost Medicare or Medicaid eligibility, were transplanted, or reached the end of the observation window (12/31/2005).

Covariates and descriptive variables

Demographic and clinical variables, drawn from the CMS 2728 form, included age, sex, race by ethnicity, employment status, smoking status (current at time of dialysis initiation), substance abuse (alcohol or illicit drugs), ability to ambulate and to transfer, body mass index (BMI), cause of ESRD, comorbidities, dialysis duration or vintage (before medication initiation), and dialysis modality. Ethnicity was categorized into one of four mutually exclusive groups: non-Hispanic Caucasians, non-Hispanic African-Americans, Hispanics, and Others. Body mass index (BMI) defined as dry weight was classified into 4 categories: < 20 kg/ m^2, 20–24.99 kg/m^2, 25–29.99 kg/m^2, ≥ 30 kg/m^2. Cause of ESRD was categorized as diabetes, hypertension, glomerulonephritis, or other. Comorbidities consisted of diabetes, congestive heart failure, coronary artery disease, cerebrovascular disease, and peripheral vascular disease. Because the CMS 2728 form is structured such that diabetes and hypertension may be considered either a cause of ESRD or a comorbidity, for the purposes of the present analysis, these two covariates were each considered a comorbidity if they were listed as either on the CMS 2728 form [34, 35]. Dialysis modality at time of dialysis initiation was categorized as in-center hemodialysis or self-care dialysis (home hemodialysis or peritoneal dialysis).

Medication exposure

β-blockers were divided into two subclasses: cardio-selective (atenolol and metoprolol) and non-selective (carvedilol and labetalol). We excluded all other β-blockers as they accounted for fewer than 2 % of all prescriptions in a given year [33]. New β-blocker users were those who did not have any prescriptions for a β-blocker in the first 90 days following dialysis initiation. They also had to initiate use of β-blockers within the next 90-day window, e.g., days 91–180 on chronic dialysis, so as to limit bias from the potential accrual of new cardiovascular risks over time. Persons were assigned to a single β-blockers subclass: anyone who used medications from both subclasses of β-blockers during the follow-up period was excluded. However, switching was extremely rare: only 30 subjects switched from cardio-selective to non-selective agents and 28 subjects switched from non-selective to cardio-selective agent. Persons using other β-blockers were also excluded.

In order to determine whether the durations of exposure were comparable between β-blocker subclasses, we examined their proportion of days covered [36]. The proportion of days covered is computed from converting days supplied and dates from individual drug claims to a daily array. The proportion of days covered was adjusted for overlapping prescription fills, hospital, and skilled nursing facility days (since medications administered throughout the institutionalization would not result in an outpatient drug claim).

Outcomes

All-cause mortality (ACM) was ascertained from the USRDS Core CD, which specifies the date and cause of death. In addition, we created a combined cardiovascular morbidity and mortality (CVMM) event outcome, capturing the first event per person. CVMM was defined as an inpatient hospitalization (Medicare Part A claims) for myocardial infarction (ICD-9 codes 410.x0, 410.x1), ischemic heart disease (411.xx), revascularization (ICD9 procedure codes 36.xx except 36.9), congestive heart failure (428.xx, 402.x1, 404.x1, or 404.x3), cerebrovascular accident (433.xx, 434.xx, 435.x), or peripheral vascular disease (440.2-4, 443.1, 443.81, 443.9, 444.2x, 444.81, 445.0x). Cardiovascular-related mortality was derived from the USRDS listed cause of death (myocardial infarction, atherosclerotic heart disease, cardiomyopathy, cardiac arrhythmia, cardiac arrest, cerebrovascular accidents). Outcome events were quantified as time from initiation of their β-blocker to either the event or censoring.

Statistical analyses

To examine balance between subclasses (cardio-selective versus non-selective β-blockers) we generated contingency tables, comparing these groups using Pearson's chi-square test and assessing validity by examining expected cell counts for categorical measures. For continuous measures, descriptive statistics were generated, stratified histograms were examined, and two-sample *t*-tests performed. To investigate within-class comparative effectiveness, we examined these data using Kaplan-Meier survival curves for an unadjusted comparison by stratifying by subclass. We then fit Cox proportional hazards regression models for ACM and CVMM outcome to compare the subgroups, adjusting for potential confounding through covariate adjustment. Model sample sizes were different for two reasons. A patient could have had a cardiovascular event before receiving a β-blocker, thus being eligible for the ACM model but not the CVMM model. Alternatively, a patient could have been on a both a cardio-selective and non-selective β-blocker during their time to mortality, but only a single subclass during their time to CVMM, thus being eligible for the CVMM model but not the ACM model.

Exponentiation of the parameter estimates obtained from these models using appropriate contrast statements allowed us to calculate the hazard ratios (HRs) for evaluating cardio-selective relative to non-selective β-blockers. Cox proportionality assumptions were ascertained through visual assessment of the complementary log-log survival plots.

Statistical significance was inferred when $P < 0.01$. All statistical analyses were done with SAS 9.2 (SAS Institute, Inc.).

Sensitivity analyses

To test the robustness of our results, we performed several sensitivity analyses. First, we expanded the analysis to individuals who initiated cardio-selective versus non-selective β-blockers at any time while on dialysis. Second, to explore the potential impact of heart failure (HF) on our results, we modeled an interaction term between β-blocker subclass and HF as identified on the CMS 2728 form. This approach was selected because use of claims to determine true HF is particularly problematic in dialysis patients, with frequent misclassification of volume overload (typically resulting from inadequate ultrafiltration or missed dialysis treatments) as HF. Finally, we examined interaction terms for β-blocker subclass and race and coronary artery disease to verify the robustness of our analyses across these subpopulations.

Ethics, consent and permissions

The research protocol received an expedited approval and HIPAA waiver by the institutional review board (Human Subjects Committee, #11436) at the University of Kansas Medical Center. Data Use Agreements (DUA) between the University and the USRDS (DUA #s: 2007–10, 2009–19, and 2015–2) and CMS (DUA #s: 16977 and 19707) permitted the data linking across the USRDS, Medicare and Medicaid files.

Results

Of the initial 84,670 cohort, 52,922 with hypertension met criteria for observability (Fig. 1). More than one-third (37.2 % or 19,708) received a β-blocker prescription during their entire window of observation. There were 4938 who had no β-blocker use in the first 90 days on dialysis but started one in the next 90 days: they were included in the ACM model. The CVMM model sample was slightly smaller at 4537.

For the ACM model, new users included 3781 (76.6 %) who were exposed to atenolol or metopropolol (cardio-selective) and 1157 (23.4 %) who were exposed to carvedilol or labetalol (non-selective). In the CVMM model, 77.0 % of the new users were exposed to a cardio-selective β-blocker and the remaining 23.0 % received non-selective β-blockers. The baseline characteristics of the cohorts for both models are shown in Table 1. In both analytic cohorts (ACM and CVMM), non-selective β-blocker users were significantly younger by 2.1–2.5 years, less likely to be Caucasian and more likely to be African-American, and more likely to have heart failure. Primary cause of ESRD did not differ significantly between cardio-selective and non-selective β-blocker users in either model. The proportion of days covered for cardio-selective β-blockers was slightly higher (0.56 versus 0.54, $p = 0.0043$) in the ACM model, but they did not differ statistically in the CVMM Model. The overall distributions were quite comparable across both models, though, and as such we did not further adjust for proportion of days covered in the statistical models.

Nearly a third of each subclass cohort died, 33.0 % for cardio-selective β-blocker users and 32.7 % for non-selective β-blocker users. Cardiovascular causes accounted for 45.6 % of deaths: principally cardiac arrest with specific cause unknown (25.1 % of all deaths). CVMM rates (46.5 %) were also comparable for cardio-selective and non-selective β-blocker users. HF accounted for 53.3 % of hospitalization events, followed by CAD (including revascularization) at 20.8 %. Cerebrovascular events accounted for 14.6 % and peripheral vascular disease accounted for 11.3 % of hospitalizations.

Survival time and time to CVMM events by the Kaplan-Meier method are shown graphically in Fig. 2a (ACM) & Fig. 2b (CVMM). For both ACM and CVMM, individuals prescribed cardio-selective β-blockers eventually had superior outcomes compared to those prescribed non-selective β-blockers. In the case of ACM, 50 % mortality was reached approximately 35.6 months

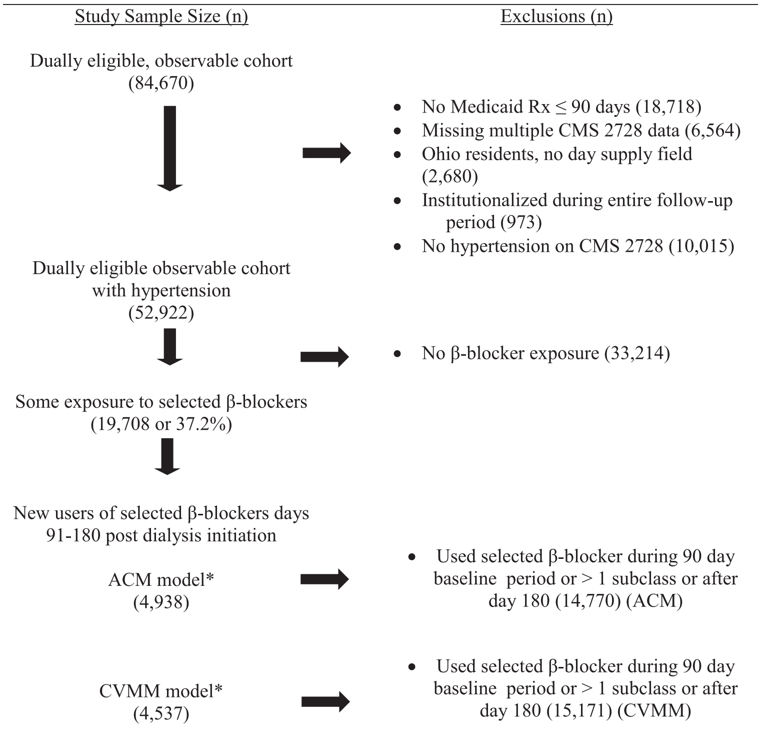

*Total individuals included differed in the models due to specific aspects of the study design. Thus some individuals could be in one model and not the other. For example, a person could be in the ACM model but not the CVMM model if they started a β-blocker prescription after a cardiovascular event; in distinction, as person could be in the CVMM model but not the ACM model if they switched β-blocker classes between a cardiovascular event and death (thus causing elimination from the ACM model).

Abbreviations: Rx, prescription; CMS, Centers for Medicare and Medicaid Services; ACM, all-cause mortality; CVMM, cardiovascular morbidity and mortality

Fig. 1 Flowchart demonstrating creation of the study cohort for new users of selected β-blockers

for cardio-selective β-blockers users and 29.4 months for non-selective β-blocker users. For CVMM, 50 % CVMM events were reached approximately 19.7 months for cardio-selective β-blockers users and 15.8 months for non-selective β-blocker users.

Adjusted for all other factors (Table 2), use of cardio-selective β-blockers, compared to non-selective β-blocker use, was associated with a lower risk of mortality (AHR = 0.84; 99 % CI = 0.72–0.97, $p = 0.0026$). Several other variables were significantly associated with a higher risk for mortality: age (AHR per decade = 1.15; 99 % CI = 1.00–1.33), Caucasian race (AHR = 1.29; 99 % CI = 1.10–1.33), low BMI (AHR = 1.38; 99 % CI = 1.11–1.72), comorbidity burden (AHR = 1.09; 99 % CI = 1.06–1.11), and self-care dialysis (AHR = 1.36; 99 % CI = 1.01–1.83). In the CVMM model, cardio-selective β-blockers were similarly associated with a lower risk for events (AHR = 0.86; 99 % CI = 0.75–0.99, $p = 0.0042$). Only age (AHR per decade = 1.11; 99 % CI = 1.06–1.16) and the comorbidity burden (AHR = 1.07; 99 % CI = 1.04–1.09) were significantly associated with CVMM events.

In the sensitivity analyses using all new users β-blockers, the effectiveness of cardio-selective β-blockers was slightly higher (ACM model- AHR = 0.79; 99 % CI = 0.72–0.87, $P < 0.0001$: CVMM model AHR = 0.80; 99 % CI = 0.73–0.89, $P < 0.0001$). In separately run sensitivity analyses, the interaction term for HF and β-blocker subclass was not significant in either the ACM model ($p = 0.72$) or the CVMM model ($p = 0.83$). Tests for interactions between African-American race and β-blockers (ACM model, $p = 0.90$; CVMM model, $p = 0.71$) and coronary artery disease and β-blockers (ACM model, $p = 0.30$; CVMM model, $p = 0.91$) were also not significant. Accordingly, we reported the models, above, without the interaction terms.

Discussion

With a theoretical potential for differential therapeutic effects, we examined within class effectiveness of cardio-selective versus non-selective β-blockers in patients on chronic dialysis. Among cohorts of new users of β-blockers, cardio-selective agents were associated

Table 1 Descriptive characteristics of new β-blockers medication users among chronic dialysis patients with hypertension across therapeutic subclasses

	All-cause mortality		CV event model	
	Cardio-selective	Non-selective	Cardio-selective	Non-selective
Number of cases	3781 (100 %)	1157 (100 %)	3495 (100 %)	1042 (100 %)
Age, mean years (SD)	60.4 (15.1)*	58.3 (15.9)*	60.1 (15.2)**	57.6 (16.0)**
Females, *n* (%)	2172 (57.5 %)	625 (54.0 %)	1992 (57.0 %)	559 (53.7 %)
Race/Ethnicity, *n* (%)				
African-American	1633 (43.2 %)*	580 (50.2 %)*	1531 (43.8 %)**	545 (52.3 %)**
Caucasian	1271 (33.6 %*	297 (25.7 %)*	1161 (33.2 %)**	246 (23.6 %)**
Hispanic	637 (16.9 %)*	212 (18.3 %)*	586 (16.8 %)**	191 (18.3 %)**
Other	240 (6.4 %)*	68 (5.9 %)*	217 (6.2 %)**	60 (5.7 %)**
BMI category, *n* (%)				
< 20 kg/m^2	374 (9.9 %)	101 (8.7 %)	345 (9.9 %)	90 (8.6 %)
20–24.9 kg/m^2	1101 (29.1 %)	355 (30.7 %)	1013 (29.0 %)	323 (31.0 %)
25–29.9 kg/m^2	1004 (26.6 %)	320 (27.7 %)	936 (26.8 %)	279 (26.8 %)
30+ kg/m^2	1255 (33.2 %)	369 (31.9 %)	1158 (33.1 %)	339 (32.5 %)
Missing	47 (1.2 %)	12 (1.0 %)	43 (1.2 %)	11 (1.0 %)
Current smoker, *n* (%)	284 (7.5 %)	63 (5.5 %)	261 (7.5 %)	55 (5.3 %)
Substance abuser,* *n* (%)	91 (2.4 %)*	47 (4.1 %)*	85 (2.4 %)**	47 (4.5 %)**
Unemployed, *n* (%)	3679 (97.3 %)	1129 (97.6 %)	3397 (97.2 %)	1014 (97.3 %)
Unable to ambulate, *n* (%)	207 (5.5 %)	52 (4.5 %)	196 (5.6 %)	47 (4.5 %)
Unable to transfer, *n* (%)	61 (1.6 %)	14 (1.2 %)	58 (1.7 %)	14 (1.3 %)
Cause of ESRD, *n* (%)				
Diabetes	2008 (53.1 %)	590 (51.0 %)	1837 (52.6 %)	530 (50.9 %)
Hypertension	1109 (29.3 %)	384 (33.2 %)	1031 (29.5 %)	350 (33.6 %)
Glomerulonephritis	304 (8.0 %)	84 (7.3 %)	287 (8.2 %)	75 (7.2 %)
Other	906 (9.9 %)	99 (8.6 %)	340 (9.7 %)	87 (8.4 %)
Comorbidities, n (%)				
Diabetes	2402 (63.5 %)	730 (63.1 %)	2198 (62.9 %)	646 (62.0 %)
Congestive heart failure	1287 (34.0 %)*	453 (39.2 %)*	1158 (33.1 %)**	399 (38.3 %)**
Coronary artery disease	1006 (26.6 %)	299 (25.8 %)	912 (26.1 %)	254 (24.4 %)
Peripheral vascular disease	585 (15.5 %)	151 (13.1 %)	536 (15.3 %)*	123 (11.8 %)*
Cerebrovascular accident	468 (12.4 %)	115 (9.9 %)	429 (12.3 %)	98 (9.4 %)
Modified Liu comorbidity	6.5 ± 3.6	6.61 ± 3.8	6.3 ± 3.6	6.4 ± 3.7
ACE/ARB use, baseline	37.3 %	35.4 %	36.7 %	34.4 %
CCB use, baseline	59.7 %*	61.9 %*	59.5 %**	63.2 %**
In-center hemodialysis *n* (%)	3582 (94.7 %)	1108 (95.8 %)	3313 (94.8 %)	995 (95.5 %)
Hemoglobin > = 11	893 (23.6 %)*	225 (19.5 %)*	822 (23.5 %)**	196 (18.8 %)**
Vintage (years) when drug initiated, mean (SD)	0.10 (0.07)	0.10 (0.07)	0.10 (0.07)	0.10 (0.07)
Proportion days covered, mean (SD)	0.56 (0.28)*	0.53 (0.28)*	0.56 (0.28)	0.54 (0.28)
Mortality, n (%)	1246 (33.0 %)	379 (32.7 %)		
CV event, n (%)			1627 (46.5 %)	485 (46.5 %)

BMI body mass index, *ESRD* end stage renal disease, *ACEI* angiotensin converting enzyme inhibitor, *ARB* angiotensin receptor blocker, *CCB* calcium channel blocker
*$p < 0.01$ for differences between subclasses within ACM model
**$p < 0.01$ for differences between subclasses within CVMM model

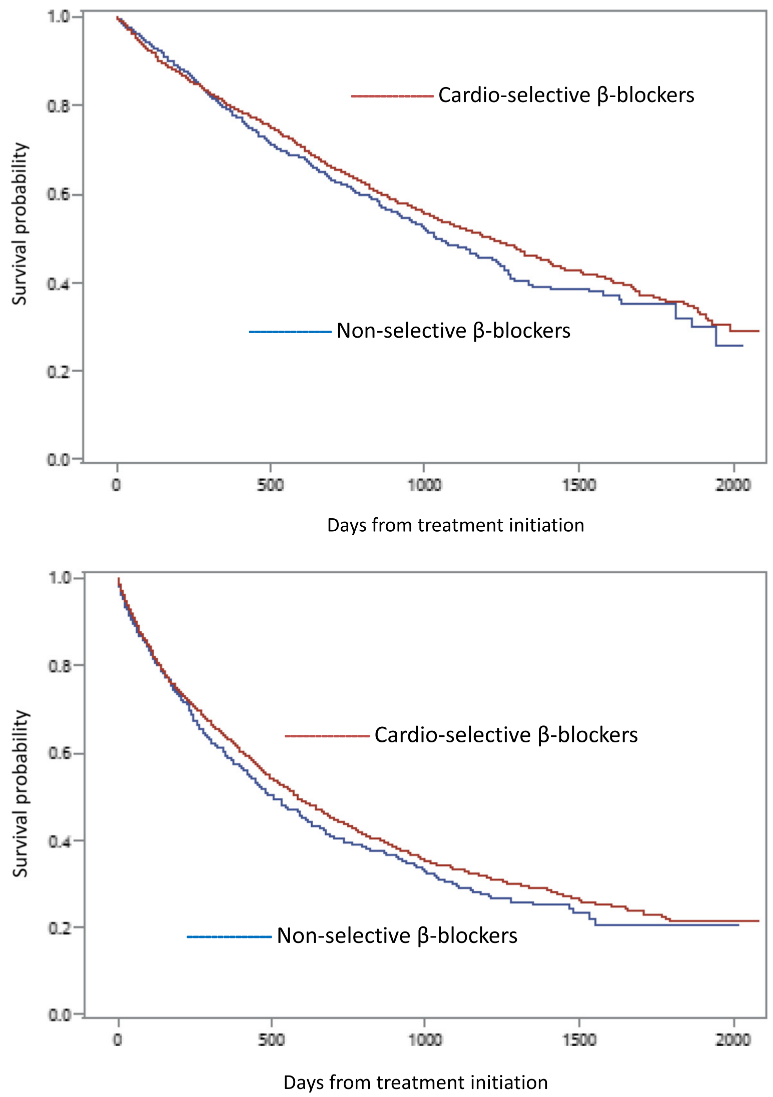

Fig. 2 Kaplan-Meier survival curves for new users of beta-blockers. **a** All-cause mortality model (top) . **b** Cardiovascular morbidity and mortality model (bottom)

with a significant 16 % reduction in mortality relative to non-selective β-blockers. A similar reduction in cardiovascular morbidity and mortality (14 %) was also noted in this large scale, observational study. Benefits were consistent regardless of underlying HF, CAD, and among African-American subjects.

Given the dearth of relevant literature, these findings constitute new insights for clinicians managing chronic dialysis patients. In an analysis of secondary data from Kaiser Permanente of Northern California, there were no significant differences in HF readmissions between metoprolol, atenolol, and carvedilol users with HF [29]. Only 5 % of the cohort was receiving chronic dialysis, and among those who were, most received either atenolol or metoprolol, similar to our cohort. A meta-analysis of carvedilol compared to β-1 selective agents netted eight trials, which when collectively analyzed, showed that carvedilol reduced all-cause mortality by 15 % in HF patients though there was not a significant reduction in HF readmissions [27]. Mortality benefits were higher (45 %) in AMI patients in three comparative trials but not consistently significant across fixed and random effect models. These studies, however, rarely included chronic dialysis patients, limiting their applicability to such patients.

It is widely appreciated that β-blockers are heterogeneous in their pharmacokinetics and potential mechanisms of action [37, 38]. In our analyses, we chose to focus on the clinical implications of the cardio-selectivity properties of β-blockers in the dialysis patient. Atenolol and metoprolol have greater β-1 selectivity and lower blood pressure through reducing cardiac output without effecting vascular resistance [27, 39]. In terms of pharmacokinetics, both are

Table 2 Comparative effectiveness of cardio-selective vs non-selective β-blockers in persons on chronic dialysis with respect to mortality (ACM) and cardiovascular morbidity-mortality (CVMM)

	ACM		CV event model	
	AHR	99 % CI	AHR	99 % CI
Cardio-selective vs. non-selective	0.84*	0.72–0.97	0.86*	0.75–0.99
Vintage (start of β-blocker)	1.71	0.69–4.28	0.85	0.38–1.90
Age, 10 year increments	1.15*	1.00–1.33	1.11*	1.06–1.16
Female sex	1.01	0.92–1.10	1.09	0.97–1.23
Race/Ethnicity				
Caucasian	1.29*	1.10–1.50	1.09	0.95–1.25
African-American	1.0	–	1.0	–
Hispanic	0.85	0.70–1.03	0.97	0.82–1.13
Other	0.79	0.59–1.06	0.98	0.77–1.25
BMI category				
< 20 kg/m^2	1.38*	1.11–1.72	1.16	0.94–1.43
20–24.9 kg/m^2	1.0	–	1.0	–
25–29.9 kg/.m^2	0.84	0.71–1.00	1.01	0.87–1.17
30+ kg/m^2	0.85	0.72–1.00	0.97	0.84–1.13
Missing BMI	1.00	0.55–1.80	1.18	0.81–1.73
Current smoker	1.05	0.83–1.38	1.18	0.71–1.96
Substance abuser	1.47	0.98–2.23	0.94	0.64–1.38
Unemployed	1.58	0.74–3.36	1.32	0.83–2.12
Inability to ambulate	1.25	0.93–1.69	1.00	0.75–1.32
Inability to transfer	1.20	0.72–1.99	0.85	0.50–1.46
Comorbidities				
Diabetes	0.93	0.80–1.09	1.04	0.91–1.19
Congestive heart failure	0.92	0.79–1.07	1.00	0.87–1.15
Coronary artery disease	1.04	0.89–1.21	1.06	0.93–1.22
Cerebrovascular accident	1.11	0.91–1.34	0.96	0.80–1.15
Peripheral vascular disease	1.00	0.83–1.20	1.09	0.92–1.29
Comorbidity burden (mod Liu)	1.09*	1.06–1.11	1.07*	1.04–1.09
Hemoglobin > = 11	1.00	0.86–1.17	0.96	0.84–1.11
Hemoglobin missing	1.05	0.83–1.33	0.98	0.80–1.20
Self-care dialysis	1.36*	1.01–1.83	1.16	0.90–1.50

AHR adjusted hazards ratio, *CI* confidence interval, *BMI* body mass index
*$p < 0.01$

removed by dialysis. In contrast, carvedilol and labetalol effect α, β-1, and β-2 receptors and are not removed by dialysis [37, 38]. β-blockers appear to exert some of their impact centrally and impact vagal tone; this would appear to give theoretical advantages to the more lipophilic, non-selective agents [40]. Carvedilol, labetalol, and metoprolol are moderately lipophilic [37, 38]: the clinical implications of this would bias our findings toward the null since we considered metoprolol use in conjunction with atenolol. Furthermore, rapid changes in drug levels associated with dialysis of more hydrophilic agents such as atenolol might also be theorized to predispose patients to sympathetic overload during the peri-dialysis period [38]. These theoretical issues, however, were not confirmed in our study. In fact, we observed the opposite impact.

Carvedilol, in particular, has been singled out as the favored β-blocker in the dialysis setting [38]. The non-selective β-blockers offer α-blockade resulting in vasodilation and lower peripheral resistance without changes in cardiac output [27, 37, 38]. In addition, carvedilol reduces cardiac adrenergic activity while β-1 selective agents increase sensitivity to adrenergic activity [11]. Carvedilol may have pleiotropic effects (antioxidant & vasodilating) and antiarrhythmic effects which might lead to less sudden death [9]. β-2 receptors play a critical role in potassium influx into cells, and β-2 receptor antagonists can increase the risk of hyperkalemia [41]. The clinical implications of this have never been fully explored, even though patients on hemodialysis can have tremendous shifts in potassium both during and between dialysis episodes. Emerging data also suggest that β-2 stimulation may actually reduce apoptosis of damaged myocytes; a combination of β-1 blockade with certain β-2 agonists has demonstrated positive effects on ventricular remodeling in animal models [42]. This emerging data combined with the results of our observational data suggests the need for more extensive studies on the role of the β-2 receptor in patients with ESRD.

Our study has several important limitations. First, as an observational study, our investigation cannot prove causality. Only a randomized clinical trial would be able to definitively answer whether cardio-selective β-blockers are truly superior to non-selective β-blockers in reducing all-cause mortality and cardiovascular events. The most obvious treatment selection concern is the presence of heart failure and the theoretical advantages offered by carvedilol. While HF was slightly more prevalent in the non-selective cohort (about 5 % higher), there was no significant interaction between HF and β-blocker subclass. We did lack important patient-level clinical measures such as blood pressure level and ejection fractions. These factors might be unbalanced between the treatment groups and, therefore, be a source of residual confounding. Nevertheless, the majority of observed differences between treatment groups at baseline were minimal, and there was also no apparent therapeutic advantage in the first year or so of follow-up, which suggests that there were no major differences in baseline clinical factors. Any unmeasured, residual confounders would need to be both common and substantial to account for the large effect size that we observed in this study. We did contemplate a

propensity adjustment, but the distributions of measured, baseline factors were so well-balanced that the approach would not have afforded much benefit.

We limited the look-back period for prior β-blocker use to 90 days to establish new use; this may be an imperfect approach, since patients may have been exposed to β-blockers in their more distant medical history, and therefore not been truly treatment naïve. We also limited the capture of new exposures to persons in the first six months of dialysis treatment so as to limit changes in underlying cardiovascular risks, but undoubtedly, subjects' clinical status may have changed during this period of time. We did not include any measure of dose which might reflect the extent of β-blockade, but there is little reason to believe that with these new users, clinicians were using radically different dosing approaches across the two subclasses. While the study period is dated, 2000–2005, there have not been any major therapeutic breakthroughs within either subclass. Outpatient prescription medications were not covered under Medicare during this period, requiring us to use a Medicare-Medicaid eligible cohort. There is no physiologic reason to argue why dually enrolled beneficiaries would experience a different response as compared to the entire chronic dialysis population. In fact, our study cohort was younger and included more women and minorities than most Medicare only cohorts, providing greater generalizability. In addition, more contemporary studies of Medicare Part D prescription drug data would be limited as many β-blockers are available as $4 prescriptions which are not well captured in Part D claims. Other important strengths include use of a large sample size, employment of a design which focused on new users of the medications, demonstration of comparable levels of exposure between cardio-selective and non-selective β-blockers, and consistency in the results across sensitivity analyses.

Conclusions

Among new initiating β-blocker users, chronic dialysis patients who received cardio-selective agents (metoprolol or atenolol) incurred a survival and cardiovascular endpoint advantage over their peers who received non-selective agents (carvedilol or labetalol). These findings may reflect the different mechanisms of action of these two medication subclasses. The initiation of dialysis is an appropriate time for providers to reconsider the ideal antihypertensive regimen for their patients. While it is unlikely that any pharmaceutical company would pursue a randomized clinical trial that compares subclasses of β-blockers in the dialysis population, the widespread use of these medications and their potential public health impact do point toward the need for a prospective comparative effectiveness trial.

Abbreviations

ACM: all-cause mortality; AHR: adjusted hazard ratio; BMI: body mass index; CAD: coronary artery disease; CI: confidence interval; CMS: Centers for Medicare & Medicaid Services; CVD: cardiovascular disease; CVMM: cardiovascular morbidity and mortality; ESRD: end-stage renal disease; HF: heart failure; USRDS: United States Renal Data System.

Competing interests

The authors declare that they have no competing interests.

Authors' contributions

TIS conceived of the study, participated in its design, oversaw programming and data analytics, drafted, and revised the manuscript. EFE participated in the conceptualization of the study questions and design, contributed to the interpretation of the findings, and help revised and approved the final manuscript. JDM and MAP helped with the operationalization of the study design, oversaw all programming activities, and lead the statistical analyses in addition to contributing to and approving the final manuscript. All authors read and approved the final manuscript.

Acknowledgements

Our thanks go out to Dr. Sally Rigler who assisted with early study design and variable definitions for several years as a member of our team.

Funding

Funding for this study was provided by NIH (NIDDK) grant R01 DK080111 (T.I.S.).

Disclaimer

The data reported here have been supplied by the United States Renal Data System (DUA#2007-10, 2009–19, & 2015–02) and the Centers for Medicare & Medicaid Services (DUA#16977 & 19707). The interpretation and reporting of these data are the responsibility of the author(s) and in no way should be seen as an official policy or interpretation of the U.S. government.

Author details

[1]Health Services Policy & Practice and the Center for Gerontology & Health Care Research, Brown University School of Public Health, 121 South Main St, Box-G-S121-6, Providence, RI 02912, USA. [2]Biostatistics, University of Kansas School of Medicine, Kansas City, KS, USA. [3]Preventive Medicine and Public Health, University of Kansas School of Medicine, Kansas City, KS, USA. [4]Medicine, University of Kansas School of Medicine, Kansas City, KS, USA.

Received: 25 July 2015 Accepted: 19 March 2016
Published online: 25 March 2016

References

1. United States Renal Data System. USRDS 2012 Annual Data Report: Atlas of End-Stage Renal Disease in the United States. In: National Institutes of Health, National Institute of Diabetes and Digestive and Kidney Diseases. 2012.
2. Wetmore JB, Mahnken JD, Rigler SK, Ellerbeck EF, Mukhopadhyay P, Hou Q, et al. Impact of race on cumulative exposure to antihypertensive medications in dialysis. Am J Hypertens. 2013;26(2):234–42.
3. St Peter WL, Sozio SM, Shafi T, Ephraim PL, Luly J, McDermott A, et al. Patterns in blood pressure medication use in US incident dialysis patients over the first 6 months. BMC Nephrol. 2013;14:249.
4. Dahlof B, Sever PS, Poulter NR, Wedel H, Beevers DG, Caulfield M, et al. Prevention of cardiovascular events with an antihypertensive regimen of amlodipine adding perindopril as required versus atenolol adding bendroflumethiazide as required, in the Anglo-Scandinavian Cardiac Outcomes Trial-Blood Pressure Lowering Arm (ASCOT-BPLA): a multicentre randomised controlled trial. Lancet. 2005;366(9489):895–906.
5. Jamerson K, Weber MA, Bakris GL, Dahlof B, Pitt B, Shi V, et al. Benazepril plus amlodipine or hydrochlorothiazide for hypertension in high-risk patients. N Engl J Med. 2008;359(23):2417–28.
6. Mohan IK, Khan M, Wisel S, Selvendiran K, Sridhar A, Carnes CA, et al. Cardioprotection by HO-4038, a novel verapamil derivative, targeted against ischemia and reperfusion-mediated acute myocardial infarction. Am J Physiol Heart Circ Physiol. 2009;296(1):H140–51.
7. Ahmed LA, Salem HA, Attia AS, El-Sayed ME. Enhancement of amlodipine cardioprotection by quercetin in ischaemia/reperfusion injury in rats. J Pharm Pharmacol. 2009;61(9):1233–41.

8. Kojima M, Sato K, Kimura G, Ueda R, Dohi Y. Carvedilol reduces elevated B-type natriuretic peptide in dialyzed patients without heart failure: cardioprotective effect of the beta-blocker. J Cardiovasc Pharmacol. 2007;49(4):191–6.
9. Dargie HJ. Effect of carvedilol on outcome after myocardial infarction in patients with left-ventricular dysfunction: the CAPRICORN randomised trial. Lancet. 2001;357(9266):1385–90.
10. Gottlieb SS, McCarter RJ, Vogel RA. Effect of beta-blockade on mortality among high-risk and low-risk patients after myocardial infarction. N Engl J Med. 1998;339(8):489–97.
11. Packer M, Coats AJ, Fowler MB, Katus HA, Krum H, Mohacsi P, et al. Effect of carvedilol on survival in severe chronic heart failure. N Engl J Med. 2001;344(22):1651–8.
12. Bouzamondo A, Hulot JS, Sanchez P, Cucherat M, Lechat P. Beta-blocker treatment in heart failure. Fundam Clin Pharmacol. 2001;15(2):95–109.
13. Cleophas TJ, Zwinderman AH. Beta-blockers and heart failure: meta-analysis of mortality trials. Int J Clin Pharmacol Ther. 2001;39(9):383–8.
14. Haas SJ, Vos T, Gilbert RE, Krum H. Are beta-blockers as efficacious in patients with diabetes mellitus as in patients without diabetes mellitus who have chronic heart failure? A meta-analysis of large-scale clinical trials. Am Heart J. 2003;146(5):848–53.
15. Foley RN, Herzog CA, Collins AJ. Blood pressure and long-term mortality in United States hemodialysis patients: USRDS Waves 3 and 4 Study. Kidney Int. 2002;62(5):1784–90.
16. Ishani A, Herzog CA, Collins AJ, Foley RN. Cardiac medications and their association with cardiovascular events in incident dialysis patients: cause or effect? Kidney Int. 2004;65(3):1017–25.
17. McCullough PA, Sandberg KR, Borzak S, Hudson MP, Garg M, Manley HJ. Benefits of aspirin and beta-blockade after myocardial infarction in patients with chronic kidney disease. Am Heart J. 2002;144(2):226–32.
18. Nakao K, Makino H, Morita S, Takahashi Y, Akizawa T, Saito A, et al. Beta-blocker prescription and outcomes in hemodialysis patients from the Japan Dialysis Outcomes and Practice Patterns Study. Nephron Clin Pract. 2009; 113(3):c132–9.
19. Tangri N, Shastri S, Tighiouart H, Beck GJ, Cheung AK, Eknoyan G, et al. beta-Blockers for prevention of sudden cardiac death in patients on hemodialysis: a propensity score analysis of the HEMO Study. Am J Kidney Dis. 2011;58(6):939–45.
20. Wetmore JB, Shireman TI. The ABCs of cardioprotection in dialysis patients: a systematic review. Am J Kidney Dis. 2009;53(3):457–66.
21. Abbott KC, Trespalacios FC, Agodoa LY, Taylor AJ, Bakris GL. beta-Blocker use in long-term dialysis patients: association with hospitalized heart failure and mortality. Arch Intern Med. 2004;164(22):2465–71.
22. Cice G, Ferrara L, D'Andrea A, D'Isa S, Di Benedetto A, Cittadini A, et al. Carvedilol increases two-year survivalin dialysis patients with dilated cardiomyopathy: a prospective, placebo-controlled trial. J Am Coll Cardiol. 2003;41(9):1438–44.
23. Cice G, Ferrara L, Di Benedetto A, Russo PE, Marinelli G, Pavese F, et al. Dilated cardiomyopathy in dialysis patients–beneficial effects of carvedilol: a double-blind, placebo-controlled trial. J Am Coll Cardiol. 2001;37(2):407–11.
24. Tory K, Horvath E, Suveges Z, Fekete A, Sallay P, Berta K, et al. Effect of propranolol on heart rate variability in patients with end-stage renal disease: a double-blind, placebo-controlled, randomized crossover pilot trial. Clin Nephrol. 2004;61(5):316–23.
25. Phadnis MA, Shireman TI, Wetmore JB, Rigler SK, Zhou X, Spertus JA, et al. Estimation of drug effectiveness by modeling three time-dependent covariates: an application to data on cardioprotective medications in the chronic dialysis population. Stat Biopharm Res. 2014;6(3):229–40.
26. Shireman TI, Phadnis MA, Wetmore JB, Zhou X, Rigler SK, Spertus JA, et al. Antihypertensive medication exposure and cardiovascular outcomes in hemodialysis patients. Am J Nephrol. 2014;40(2):113–22.
27. DiNicolantonio JJ, Lavie CJ, Fares H, Menezes AR, O'Keefe JH. Meta-analysis of carvedilol versus beta 1 selective beta-blockers (atenolol, bisoprolol, metoprolol, and nebivolol). Am J Cardiol. 2013;111(5):765–9.
28. Wali RK, Iyengar M, Beck GJ, Chartyan DM, Chonchol M, Lukas MA, et al. Efficacy and safety of carvedilol in treatment of heart failure with chronic kidney disease: a meta-analysis of randomized trials. Circ Heart Fail. 2011;4(1):18–26.
29. Go AS, Yang J, Gurwitz JH, Hsu J, Lane K, Platt R. Comparative effectiveness of beta-adrenergic antagonists (atenolol, metoprolol tartrate, carvedilol) on the risk of rehospitalization in adults with heart failure. Am J Cardiol. 2007;100(4):690–6.
30. Pasternak B, Svanstrom H, Melbye M, Hviid A. Association of treatment with carvedilol vs metoprolol succinate and mortality in patients with heart failure. JAMA Intern Med. 2014;174(10):1597–604.
31. Wetmore JB, Mahnken JD, Rigler SK, Ellerbeck EF, Mukhopadhyay P, Hou Q, et al. Association of race with cumulative exposure to statins in dialysis. Am J Nephrol. 2012;36(1):90–6.
32. Wetmore JB, Rigler SK, Mahnken JD, Mukhopadhyay P, Shireman TI. Considering health insurance: how do dialysis initiates with Medicaid coverage differ from persons without Medicaid coverage? Nephrol Dial Transplant. 2010;25(1):198–205.
33. Wetmore JB, Mahnken JD, Mukhopadhyay P, Hou Q, Ellerbeck EF, Rigler SK, et al. Geographic variation in cardioprotective antihypertensive medication usage in dialysis patients. Am J Kidney Dis. 2011;58(1):73–83.
34. Volkova N, McClellan W, Soucie JM, Schoolwerth A. Racial disparities in the prevalence of cardiovascular disease among incident end-stage renal disease patients. Nephrol Dial Transplant. 2006;21(8):2202–9.
35. Wetmore JB, Mahnken JD, Rigler SK, Ellerbeck EF, Mukhopadhyay P, Spertus JA, et al. The prevalence of and factors associated with chronic atrial fibrillation in Medicare/Medicaid-eligible dialysis patients. Kidney Int. 2012; 81(5):469–76.
36. Avorn J, Monette J, Lacour A, Bohn RL, Monane M, Mogun H, et al. Persistence of use of lipid-lowering medications: a cross-national study. JAMA. 1998;279(18):1458–62.
37. Furgeson SB, Chonchol M. Beta-blockade in chronic dialysis patients. Semin Dial. 2008;21(1):43–8.
38. McQuillan RF, Chan CT. The intuitive case for beta-blockers in patients with ESRD. Semin Dial. 2012;25(1):15–21.
39. Messerli FH, Grossman E. beta-Blockers in hypertension: is carvedilol different? Am J Cardiol. 2004;93(9A):7B–12.
40. Hjalmarson A. Cardioprotection with beta-adrenoceptor blockers. Does lipophilicity matter? Basic Res Cardiol. 2000;95 Suppl 1:I41–5.
41. Nowicki M, Miszczak-Kuban J. Nonselective Beta-adrenergic blockade augments fasting hyperkalemia in hemodialysis patients. Nephron. 2002;91(2):222–7.
42. Rinaldi B, Donniacuo M, Sodano L, Gritti G, Martuscelli E, Orlandi A, et al. Effects of chronic treatment with the new ultra-long-acting beta2 -adrenoceptor agonist indacaterol alone or in combination with the beta1 -adrenoceptor blocker metoprolol on cardiac remodelling. Br J Pharmacol. 2015;172(14):3627–37.

Submit your next manuscript to BioMed Central and we will help you at every step:

- We accept pre-submission inquiries
- Our selector tool helps you to find the most relevant journal
- We provide round the clock customer support
- Convenient online submission
- Thorough peer review
- Inclusion in PubMed and all major indexing services
- Maximum visibility for your research

Submit your manuscript at www.biomedcentral.com/submit

BioMed Central

ORIGINAL RESEARCH

Prognostic Benefits of Carvedilol, Bisoprolol, and Metoprolol Controlled Release/Extended Release in Hemodialysis Patients with Heart Failure: A 10-Year Cohort

Chao-Hsiun Tang, PhD; Chia-Chen Wang, MD; Tso-Hsiao Chen, MD, PhD; Chuang-Ye Hong, MD, PhD; Yuh-Mou Sue, MD

***Background*—**Heart failure is a highly prevalent cardiovascular complication among patients receiving long-term hemodialysis, but the benefits of carvedilol, bisoprolol, and metoprolol controlled release/extended release on the outcomes of these patients remain unclear. In this study, we address the use of these 3 β-blockers and their associations with mortality.

***Methods and Results*—**Long-term hemodialysis patients, aged ≥35 years, with new-onset heart failure and receiving various medications were identified through the use of 1999–2010 data from the Taiwan National Health Insurance Research Database. From the total of 4435 heart failure patients, we selected 1700 new users of the 3 β-blockers (study group) and 1700 nonusers (control group), by using matched cohorts according to their propensity scores, and then compared the 5-year all-cause mortality rates by using Cox proportional hazard regressions and time-dependent covariate adjustment. During 3944 person-years of follow-up, 666 (39.2%) deaths occurred within the study group, compared with 918 (54%) deaths during 2893 person-years of follow-up in the control group. The 5-year mortality rate for the study (control) group was 54.5% (70.3%); $P<0.001$. Adjusted hazard regression analyses revealed that the therapeutic effects of β-blockers remained significant for all-cause mortality (hazard ratio 0.80, 95% CI 0.72 to 0.90). Subgroup analyses revealed that patients in the study group receiving β-blockers plus renin-angiotensin system antagonists exhibited the lowest mortality rate, while the highest mortality rate was found among patients in the control group receiving neither β-blockers nor renin-angiotensin system antagonists.

***Conclusions*—**This study demonstrates that the 3 β-blockers were associated with improved survival in long-term hemodialysis patients with heart failure. **(*J Am Heart Assoc*. 2016;5:e002584 doi: 10.1161/JAHA.115.002584)**

Key Words: end-stage renal disease • heart failure • hemodialysis • mortality • β-blocker

Heart failure (HF) is known to be a highly prevalent cardiovascular complication among patients receiving long-term hemodialysis (HD).[1] The current treatment guidelines for HF, which include those provided by the American College of Cardiology Foundation/American Heart Association and the European Society of Cardiology, recommend the use of angiotensin-converting enzyme inhibitors (ACEIs) or angiotensin type II receptor blockers (ARBs) as a means of improving the general survival rates for all patients.[2,3] These 2 sets of guidelines also recommend the use of carvedilol, bisoprolol, or metoprolol controlled release (CR)/extended release (XL), in addition to an ACEI or ARB, as a means of improving HF symptoms and a potential survival benefit.[4–9] Indeed, in patients with HF symptoms, the European Society of Cardiology further recommends administering 1 of the 3 β-blockers as the initial therapy, followed by the use of an ACEI or ARB.[3,10,11] Among the general population as a whole, the most compelling evidence on survival in cases of severe chronic HF is provided by the use of carvedilol, followed by bisoprolol and metoprolol CR/XL.[4–11]

HF is among the most frequent cardiovascular complications in HD patients, and there is currently insufficient evidence to show the benefits of β-blockers in long-term HD patients with HF. This is essentially as a result of the exclusion of this population from large clinical trials. During the past 10 years, several retrospective and observational studies have been carried out with the overall aim of evaluating the therapeutic effects of β-blockers, although the focus has tended to be on dialysis patients, and not

From the School of Health Care Administration, College of Management (C.-H.T.), School of Medicine, College of Medicine (T.-H.C., C.-Y.H., Y.-M.S.), and Department of Internal Medicine, Wan Fang Hospital (T.-H.C., C.-Y.H., Y.-M.S.), Taipei Medical University, Taipei, Taiwan; School of Medicine, Fu-Jen Catholic University, New Taipei City, Taiwan (C.-C.W.).

Accompanying Tables S1 through S6 and Figures S1 and S2 are available at http://jaha.ahajournals.org/content/5/1/e002584/suppl/DC1

Correspondence to: Yuh-Mou Sue, MD, Department of Internal Medicine, Wan Fang Hospital, Taipei Medical University, 5th Floor, No. 111, Section 3, Xing Long Road, Taipei 116, Taiwan. E-mail: sueym@tmu.edu.tw

Received September 25, 2015; accepted November 10, 2015.

© 2016 The Authors. Published on behalf of the American Heart Association, Inc., by Wiley Blackwell. This is an open access article under the terms of the Creative Commons Attribution-NonCommercial License, which permits use, distribution and reproduction in any medium, provided the original work is properly cited and is not used for commercial purposes.

Downloaded from http://jaha.ahajournals.org/ at VA MED CTR BOISE on January 12, 2016

specifically on HF patients.[12–15] The results of these studies have, however, been inconclusive, with only a few providing evidence in support of the use of β-blockers in HD patients.[13–15] Several factors may have contributed to the mixed results of these studies, including underpowered and misclassified drug exposure over time, as well as a lack of adjustment for nonrandom treatment allocation. Only a study with a small sample size (114 patients) has reported a reduction in all-cause hospitalization and all-cause mortality as a result of the addition of carvedilol to ACEI use in long-term HD patients with HF.[16] However, it remains unclear as to whether β-blockers can be used as the initial therapy regimen for long-term HD patients with HF.

According to 2005–2012 data reported in the latest Taiwan Renal Registry Data System database, Taiwan has one of the highest prevalence rates (2926/1 million population) and incidence rates (426/1 million population) of end-stage renal disease in the world.[17] Appropriate treatment for long-term HD patients with HF will prolong their survival and improve their quality of life. To provide more evidence to improve treatment methods for these patients, we set out in the present study to investigate the effects of 3 β-blockers (carvedilol, bisoprolol, and metoprolol CR/XL) on mortality rates among long-term HD patients with HF based on real-world clinical practice information obtained from the Taiwan National Health Insurance (NHI) research database (NHIRD).

Methods and Materials

Study Design and Data Sources

We carried out a nationwide retrospective cohort study based on 1999–2010 data on all patients receiving HD obtained from the NHIRD. The NHIRD provides healthcare utilization data on >99% of the entire 23 million people enrolled in the NHI program and 95% of all hospitals in Taiwan, with the *International Classification of Diseases, 9th Revision* (ICD-9) codes being used to define the diseases. The NHIRD are all delinked information and contain all registry and claim data, including the (1) outpatient expenditure, (2) inpatient expenditure, (3) registry for medical personnel with data on each medical professional's date of birth, sex, profession, and specialty, (4) registry of contracted medical facilities with data on each medical institution's accreditation level and geographical location, and (5) registry for patients with catastrophic illness with data for 30 illness and injury categories. The data set has been used for epidemiologic research, and the results have been validated for several diseases, including acute kidney injury, chronic kidney disease, coronary artery disease, congestive HF, and diabetes mellitus.[18–21] This study was approved by the Joint Institutional Review Board of Taipei Medical University, and informed consent was waived because the personal information had been delinked in the NHIRD.

Study Population and Cohorts

We first defined and identified long-term HD patients who had undergone ≥26 HD sessions within 3 months of commencing HD. A total of 74 838 patients who received long-term HD were identified by using the catastrophic illness registry in the NHIRD from 2001 to 2010, with 1999–2010 NHIRD data being used for comorbidity evaluations and follow-up analysis purposes. We then defined the HF patients based on the ICD-9 codes. The codes for HF are 401.91, 402.01, 402.11, 404.01, 404.03, 404.11, 404.91, 404.93, and 428. Our sample included new-onset HF patients after they started to receive HD. The inclusion criteria for HF patients were (1) ≥3 outpatient visit claims with an HF diagnosis within 365 days or (2) 1 claim for incident hospitalization with an HF diagnosis. Figure 1 provides a schematic illustration of the sample selection. The β-blockers examined in this study were carvedilol, bisoprolol, or metoprolol CR/XL for ≥30 days, because only these 3 β-blockers are proved to have survival benefits for HF patients. The exclusion criteria were (1) patients diagnosed with HF before HD, (2) patients taking these β-blockers for <30 days, (3) patients taking β-blockers within the 3-month period before HF diagnosis (ie, washout period), (4) patients using β-blockers other than our 3 focus β-blockers, and (5) patients who did not take any antihypertensive drug. We also defined comorbidities by using the same criteria, according to the ICD-9 codes, as shown in Table 1.

The sample patients were further divided into 2 subgroups, the "study group" who were defined as new users of the 3 β-blockers following their HF diagnosis, and the "control group" who had never used any β-blockers after their HF diagnosis. We were ultimately left with 2095 patients as the study group and 2340 patients as the control group (Figure 1).

Propensity Score Computation and Matching

We used propensity score (PS) analyses to adjust for any differences in the baseline patient characteristics between the study and control groups and to reduce the biases in the estimation process that may be attributable to such differences.[22] The scores were computed by modeling a logistic regression with use of the variables of patient age and sex, HD duration at enrollment, the number of hospitalizations, Charlson comorbidity index, all comorbidities, and performed tests/procedures (Table 1) and medication being consumed at enrollment, as reported in Table 2. Prescription data were classified according to the Anatomical Therapeutic and Chemical Classification System. The logistic regression model used to calculate the PS and the distribution of PS among the

Downloaded from http://jaha.ahajournals.org/ at VA MED CTR BOISE on January 12, 2016

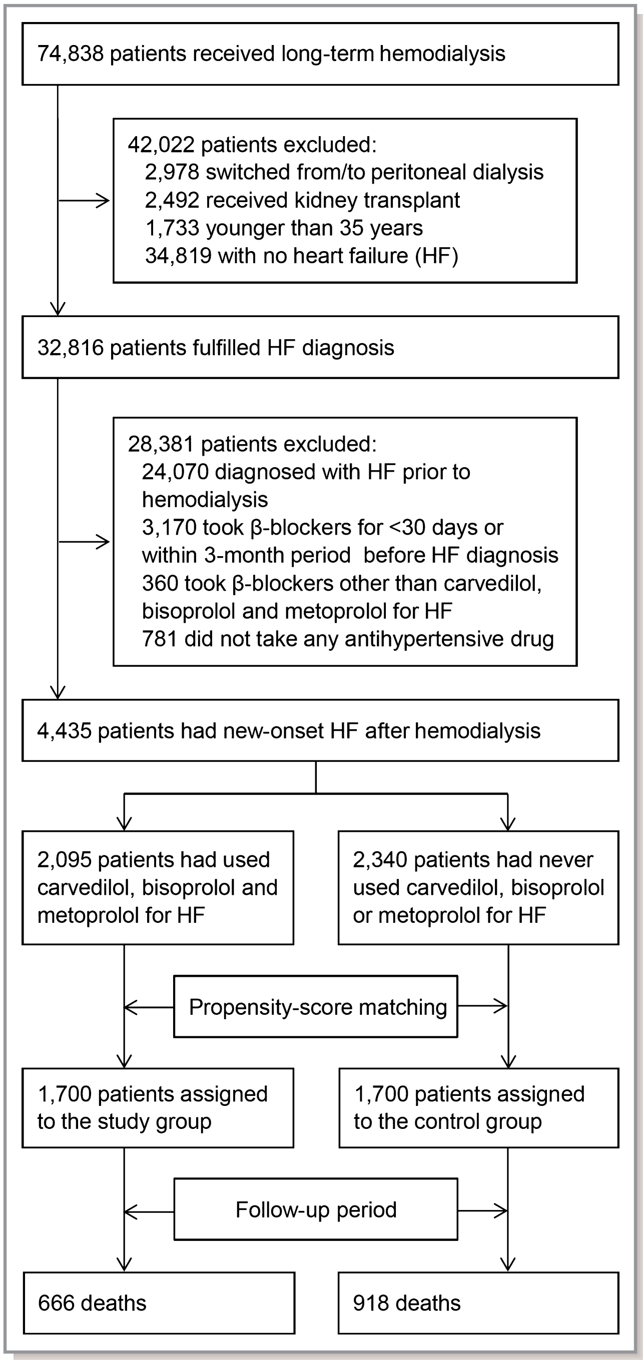

Figure 1. Enrollment of study participants.

treatment and control groups are shown in Tables S1 and S2. The c-statistic value for the model used to create the PS was 0.66, indicating that the predictive accuracy of the logistic model was fairly good.[23] We used the "nearest neighbor matching without replacement on the estimated PS" to match patients.[24] Patients receiving the 3 β-blockers were matched 1:1 to untreated patients with a difference in PS of ≤0.1. Finally, 81% of the treated patients were matched to a control and 1700 patients were identified in each group (Figure 1, Table 1, Table S3, and Table 2).

Outcome Measures

The main outcome of interest in this study was the all-cause mortality within the 5-year period after the index date, with the first β-blocker prescription after HF diagnosis being defined as the index date for the study group to prevent immortal time bias, and the date of HF diagnosis being defined as the index date for the control group. The HF patients were subsequently followed up for a period of up to 5 years or until the date of death. A death event was identified if the date of death was obtained from the NHIRD or the patients were disenrolled from the NHI program and had not been enrolled in the NHI beneficiary registry files. In Taiwan, patients receiving long-term HD rarely withdrew from the NHI because the NHI is a compulsory plan. The censor criteria included patients who were followed for up to 5 years until the last day of follow-up (December 31, 2010) or death (whichever happened first). Patients who switched from or to peritoneal dialysis and received a kidney transplant after starting long-term HD were already excluded (Figure 1). Further subgroup analysis was carried out according to the use of ACEIs or ARBs during the follow-up period.

Statistical Analysis

The first of the analyses in this study involved a comparison between the characteristics of the study group and those of the control group at the baseline. We estimated absolute standardized differences for all the covariates after matching to assess postmatch balance. Absolute standardized differences directly quantify balance in the means of covariates across the groups. The differences are displayed as percentages of pooled standardized differences. An absolute standardized difference of 0% on a covariate indicates no between-group imbalance for that covariate, and values <10% indicate inconsequential imbalance.[25] We used an independent-sample Student *t* test to analyze the continuous variables, with the categorical variables being analyzed by using the Pearson χ^2 test. We then charted the survival curves by using the Kaplan–Meier method and subsequently examined the treatment effect with use of the log-rank test.

Finally, we applied Cox regression univariate and multivariable analyses with and without adjustment for the demographic variables (sex and age), the clinically relevant variables (diabetes, ischemic heart disease, duration of dialysis at enrollment, number of hospitalizations, and the Charlson comorbidity index), the procedures (myocardial perfusion scan, coronary angiography, and percutaneous coronary intervention), and medication at enrollment (fibrates, insulins, H2-antagonists, and proton pump inhibitors) to assess the therapeutic effects on the probability of death. The proportional hazards assumption was also tested. The

Downloaded from http://jaha.ahajournals.org/ at VA MED CTR BOISE on January 12, 2016

Table 1. Baseline Characteristics of the Full Sample and the Propensity Score–Matched Sample

Characteristics	Full Cohort: Study Group (n=2095)	Full Cohort: Control Group (n=2340)	Full Cohort: *P* Value	Matched Cohort: Study Group (n=1700)	Matched Cohort: Control Group (n=1700)	Matched Cohort: ASD (%)
Sex: male, n (%)	1051 (50.2)	1135 (48.5)	0.25	844 (49.7)	834 (49.1)	1.2
Age at cohort entry (y), mean (SD)	65.6 (11.5)	69.1 (11.4)	<0.001	67.3 (11.1)	67.5 (11.5)	1.7
35 to 44 y, n (%)	81 (3.8)	71 (3.0)	<0.001	54 (3.2)	60 (3.5)	2.4
45 to 54 y, n (%)	366 (17.5)	225 (9.6)		210 (12.4)	205 (12.1)	
55 to 64 y, n (%)	522 (24.9)	470 (20.1)		398 (23.4)	399 (23.5)	
65 to 74 y, n (%)	635 (30.3)	796 (34.0)		578 (34.0)	570 (33.5)	
≥75 y, n (%)	491 (23.4)	778 (33.2)		460 (27.1)	466 (27.4)	
Charlson comorbidity index, mean (SD)*	3.64 (3.0)	3.60 (2.8)	<0.001	3.68 (2.9)	3.64 (2.9)	1.7
No. of hospitalizations, mean (SD)*	2.7 (2.6)	2.3 (2.3)	<0.001	2.6 (2.5)	2.4 (2.3)	3.5
Duration of dialysis at enrollment (mo), mean (SD)	32.1 (24.2)	30.1 (25.5)	<0.01	32.5 (24.3)	30.9 (25.5)	6.1
Comorbidities* (ICD-9 codes), n (%)						
Ischemic heart disease (411, 413, 414)	814 (38.9)	831 (35.5)	0.02	630 (37.1)	630 (37.1)	0
Myocardial infarction (410, 412)	99 (4.7)	98 (4.2)	0.38	85 (5.0)	79 (4.7)	1.7
Cardiac dysrhythmia (426, 427)†	254 (12.1)	296 (12.6)	0.61	203 (11.9)	207 (12.2)	0.7
Cerebrovascular disease (430 to 438)	359 (17.1)	531 (22.7)	<0.001	324 (19.1)	328 (19.3)	0.6
Peripheral artery disease (440.2, 443)	95 (4.5)	91 (3.9)	0.28	69 (4.1)	73 (4.3)	1.2
Hypertension (401 to 405)	1621 (77.4)	1769 (75.6)	0.14	1300 (76.5)	1285 (75.6)	2.1
Diabetes mellitus (250)	1065 (50.8)	1217 (52.0)	0.47	858 (50.5)	914 (53.8)	6.6
COPD (491 to 493, 495 to 496)	250 (11.9)	379 (16.2)	<0.001	227 (13.4)	228 (13.4)	0.2
Cirrhosis of liver (571)	70 (3.3)	115 (4.9)	<0.01	64 (3.8)	61 (3.6)	0.9
Cancer (140 to 208)	205 (9.8)	245 (10.5)	0.46	174 (10.2)	174 (10.2)	0
Tests or procedures,* n (%)						
Echocardiography	1409 (67.3)	1452 (62.1)	<0.001	1126 (66.2)	1074 (63.2)	6.4
Myocardial perfusion scan	314 (15.0)	230 (9.8)	<0.001	242 (14.2)	183 (10.8)	10.5
Coronary angiography	223 (10.6)	171 (7.3)	<0.001	166 (9.8)	137 (8.1)	6.0
Percutaneous coronary intervention	131 (6.3)	82 (3.5)	<0.001	97 (5.7)	67 (3.9)	8.2
Coronary artery bypass graft surgery	14 (0.7)	11 (0.5)	0.38	12 (0.7)	8 (0.5)	3.1
24-Hour electrocardiogram	219 (10.5)	240 (10.3)	0.81	180 (10.6)	178 (10.5)	0.4
Permanent pacemaker implantation	15 (0.7)	26 (1.1)	0.17	13 (0.8)	17 (1.0)	2.5

ASD indicates absolute standardized difference; COPD, chronic obstructive pulmonary disease; ICD-9, *International Classification of Diseases, 9th Revision*.
*Within the 2-year period before the index date.
†Cardiac dysrhythmia includes both conduction disorders (ICD-9 code 426: atrioventricular block, bundle branch block, and anomalous atrioventricular excitation) and cardiac dysrhythmias (ICD-9 code 427: paroxysmal supraventricular and ventricular tachycardia, atrial fibrillation and flutter, ventricular fibrillation and flutter, cardiac arrest, and premature beats).

difference between the 2 groups was considered significant if the 2-sided $P<0.05$. All of the analyses in this study were carried out by using SAS 9.3 software (SAS Institute Inc).

Sensitivity Analysis

In addition to the main analysis, we performed additional analyses to assess the reliability of our results. The US National Kidney Foundation provided a Kidney Disease Outcomes Quality Initiative evidence-based clinical practice guideline for long-term HD patients with cardiovascular disease in 2005.[26] The guideline suggested the treatment of HF in patients on long-term HD with conventional therapies according to expert opinion, and the evidence for the use of a β-blocker was moderately strong (only carvedilol). To evaluate the effect of the initiative guideline on the therapeutic results

Downloaded from http://jaha.ahajournals.org/ at VA MED CTR BOISE on January 12, 2016

Table 2. Concomitant Medication at Enrollment for the Full Sample and the Propensity Score–Matched Sample*

Variables	Full Cohort			Matched Cohort		
	Study Group (n=2095)	Control Group (n=2340)		Study Group (n=1700)	Control Group (n=1700)	
	No. (%)	No. (%)	*P* Value	No. (%)	No. (%)	ASD (%)
ACEIs or ARBs	752 (35.9)	579 (24.7)	<0.001	494 (29.1)	486 (28.6)	1.0
Calcium channel blockers	1014 (48.4)	1011 (43.2)	<0.001	767 (45.1)	781 (45.9)	1.7
α-Blockers	121 (5.8)	123 (5.3)	0.44	91 (5.4)	90 (5.3)	0.3
Hydralazine	68 (3.3)	70 (3.0)	0.62	51 (3.0)	52 (3.1)	0.3
Nitrates	784 (37.4)	779 (33.3)	<0.01	602 (35.4)	596 (35.1)	0.7
Digoxin	153 (7.3)	139 (5.9)	0.07	102 (6.0)	107 (6.3)	1.2
Antiarrhythmics	208 (9.9)	243 (10.4)	0.63	165 (9.7)	157 (9.2)	1.6
Platelet inhibitors	567 (27.1)	579 (24.7)	0.07	442 (26.0)	438 (25.8)	0.5
Warfarin	46 (2.2)	59 (2.5)	0.48	37 (2.2)	43 (2.5)	2.3
Statins	275 (13.1)	244 (10.4)	<0.01	214 (12.6)	202 (11.9)	2.2
Fibrates	100 (4.8)	125 (5.3)	0.40	80 (4.7)	107 (6.3)	7.0
Oral hypoglycemic drugs	456 (21.8)	502 (21.5)	0.78	364 (21.4)	390 (22.9)	3.7
Insulins	470 (22.4)	570 (24.4)	0.14	352 (20.7)	431 (25.4)	11.1
H2-antagonists or PPIs	525 (25.1)	747 (31.9)	<0.001	419 (24.7)	537 (31.6)	15.5
NSAIDs	953 (45.5)	1075 (45.9)	0.80	762 (44.8)	780 (45.9)	2.1
Benzodiazepines†	688 (32.8)	735 (31.4)	0.29	541 (31.8)	548 (32.2)	1.0

ACEI indicates angiotensin-converting enzyme inhibitor; ARB, angiotensin type II receptor blocker; ASD, absolute standardized difference; NSAID, nonsteroidal anti-inflammatory drug; PPI, proton pump inhibitor.
*Within the 3-month period before the index date.
†Refers to benzodiazepines used as anxiolytics, hypnotics, and sedatives.

of our 10-year cohort study, we first conducted discrete analyses for patients with index dates from 2001 to 2005 and from 2006 to 2010 (before and after publication of the guideline).

Second, persistence in therapy is a potential confounding factor in this study. The patients who received β-blockers, ACEIs, or ARBs might not continue therapy during the entire follow-up period. Because the consumption of β-blocker would accumulate over time, when we followed the death event of the HF patients, the exposure duration by month of β-blocker therapy was treated as a time-dependent covariate to reflect its changing nature. In this analysis, we used the counting process method by allowing our β-blocker therapy variable to vary over time. The time-dependent variable of each individual was followed and updated at every monthly interval. For example, if an individual started β-blocker therapy for 10 months, quit for 3 months, then restarted for 2 months before dying, we would have 2 changes of β-blocker therapy status. We then set these 3 observations equal to 10 months with β-blocker therapy, 3 months without β-blocker therapy, and 2 months with β-blocker therapy then censored. Similarly, the exposure duration of ACEI or ARB therapy was treated as a time-dependent covariate because its use also has survival benefits for the HF patients.[21]

In addition, we conducted analysis by defining the observation period beginning at intervals of 30 and 45 days after the initial HF diagnosis to assess the therapeutic effects on the probability of death and to minimize the risk of potential bias.

Results

Demographic Characteristics

A total of 3400 long-term HD patients with HF were included in the PS matching in this study (Figure 1). The baseline patient characteristics before and after cohort matching are reported in Table 1, which shows that there were no significant differences in sex, age, comorbidities, tests, and procedures between the 2 groups after the cohort matching analyses, with the exceptions of myocardial perfusion scan. Table 2 also shows that concomitant medications at the time of enrollment were similar in the 2 groups, with the exceptions of insulins and H2-antagonists or proton pump inhibitors.

Downloaded from http://jaha.ahajournals.org/ at VA MED CTR BOISE on January 12, 2016

Outcomes

The primary outcome in this study is all-cause mortality during a follow-up period of up to 5 years. Among the matched cohort patients of the study group, classified by their first use of β-blockers during the follow-up period, 1008 (59.3%) patients had taken carvedilol, 629 (37%) patients had taken bisoprolol, and 63 (3.7%) patients had taken metoprolol CR/XL. The respective mean daily doses of carvedilol, bisoprolol, and metoprolol CR/XL were 16.4, 4.4, and 65.4 mg.

The respective mean (SD) durations of carvedilol, bisoprolol, and metoprolol CR/XL use were 104.5 (93.5) days/person-year, 102.2 (101.1) days/person-year, and 82.5 (69.2) days/person-year. During the 3944 person-year follow-up period, 666 deaths (39.2%) occurred in the study group, while in the 2893 person-year follow-up period in the control group, there was a substantially higher total of 918 deaths (54.0%). The respective all-cause mortality rates at the 12-, 24-, 36-, 48-, and 60-month follow-up periods were 19.1%, 29.1%, 38.5%, 46.2%, and 54.5%, for the study group, while the respective mortality rates for the control group over the same periods were 34.2%, 48.7%, 58.6%, 65.4%, and 70.3%. The incident rate ratios of all-cause mortality for the study and control groups from 2001 to 2005 and from 2006 to 2010 are shown in Table S4.

The Kaplan–Meier analyses of the survival proportion of the study and control groups are illustrated in Figure 2A, which shows that the study group had significantly higher survival benefits than the control group (log-rank test, $P<0.001$). We further identified the individual survival benefits of carvedilol, bisoprolol, and metoprolol CR/XL. As we can see from Figure 2B, each of the 3 β-blockers showed significant survival benefits compared with the control group, but there were no discernible differences in survival benefits between any 2 of the 3 β-blockers.

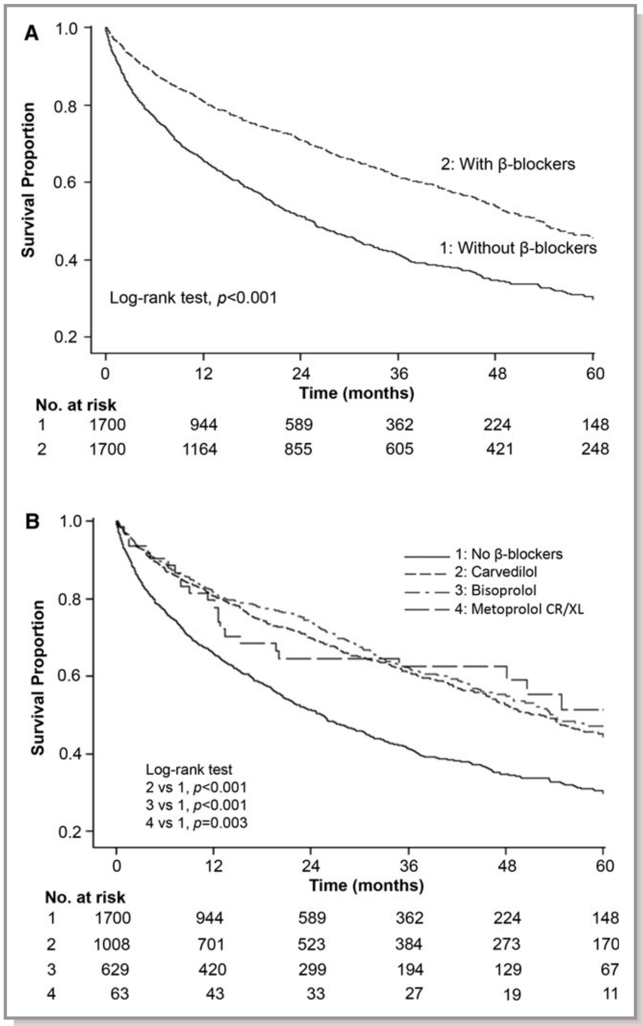

Figure 2. Kaplan–Meier estimates of hemodialysis patient survival rates in a propensity-matched inception cohort of patients with heart failure. A, Survival of patients with/without β-blockers. B, Survival of patients receiving carvedilol, bisoprolol, metoprolol CR/XL, or no β-blockers. No differences are discernible between the survival benefits for any 2 of these 3 β-blockers.

Multivariable Analysis

The Cox proportional hazard regressions on all-cause mortality are shown in Table 3. In the final model, the study group was found to have an 20% lower risk of all-cause mortality than the control group (hazard ratio [HR], 0.80, 95% CI, 0.72 to 0.90; $P<0.001$) after adjustment for the exposure duration of β-blocker therapy and the exposure duration of ACEI or ARB therapy. The survival curves of the final model after adjustment for the exposure duration of β-blocker therapy and the exposure duration of ACEI or ARB therapy are shown in Figure 3. Detailed results from 2001 to 2005 and from 2006 to 2010 can be found in Table S5 and Figures S1 and S2.

The interactions between the therapeutic effect and the clinical parameters are illustrated in Figure 4, which also reveals significant interactions when considering the sex, Charlson comorbidity index, and HD sessions per month.

HR by Subgroup

The medications used at enrollment (including ACEIs or ARBs, calcium channel blockers, hydralazine, and nitrates) were similar in both groups after cohort matching. To clarify their therapeutic effects, we further analyzed ACEI or ARB use during the follow-up period. As shown in Table 4 and Figure 5, the patients in the study group who received β-blockers plus ACEIs or ARBs had the best survival benefits, followed by those who received β-blockers alone. The patients in the control group who took neither of these 2 groups of drugs had the highest mortality rate.

Downloaded from http://jaha.ahajournals.org/ at VA MED CTR BOISE on January 12, 2016

Table 3. Cox Proportional Hazard Regression on All-Cause Mortality for the Study Group Versus the Control Group

Models and Adjustments	HR	95% CI	*P* Value
Univariate model	0.56	0.51 to 0.62	<0.001
Multivariate model			
Adjusted for diabetes	0.57	0.51 to 0.63	<0.001
Adjusted for ischemic heart disease	0.56	0.51 to 0.62	<0.001
Adjusted for duration of dialysis at enrollment	0.57	0.51 to 0.63	<0.001
Adjusted for No. of hospitalization	0.55	0.50 to 0.61	<0.001
Adjusted for Charlson comorbidity index	0.56	0.51 to 0.62	<0.001
Adjusted for various procedures*	0.55	0.50 to 0.61	<0.001
Adjusted for medication at enrollment†	0.58	0.52 to 0.64	<0.001
Final model‡	0.56	0.50 to 0.62	<0.001
Final model adjusted with time-dependent covariates			
Adjusted for the exposure duration of β-blocker therapy	0.76	0.68 to 0.85	<0.001
Adjusted for the exposure duration of β-blocker therapy and the exposure duration of ACEI or ARB therapy	0.80	0.72 to 0.90	<0.001

ACEI indicates angiotensin-converting enzyme inhibitor; ARB, angiotensin receptor blocker; HR, hazard ratio.
*The procedures include myocardial perfusion scan, coronary angiography, and percutaneous coronary intervention.
†The medications include fibrates, insulins, H2-antagonists, and proton pump inhibitors.
‡The control variables include in the final model demographic variables (sex and age), clinically relevant variables (diabetes, ischemic heart disease, duration of dialysis at enrollment, No. of hospitalizations, and Charlson comorbidity index), procedures (myocardial perfusion scan, coronary angiography, and percutaneous coronary intervention), and medications at enrollment (fibrates, insulins, H2-antagonists, and proton pump inhibitors).

Sensitivity Analyses for All-Cause Death

We further analyzed the risk of all-cause mortality with observation period beginning at intervals of 30 and 45 days after initial HF diagnosis. The results showed that the patients in the study group still had a lower risk of all-cause mortality than those in the control group with an observation period beginning at intervals of 30 and 45 days after initial HF diagnosis (Table S6).

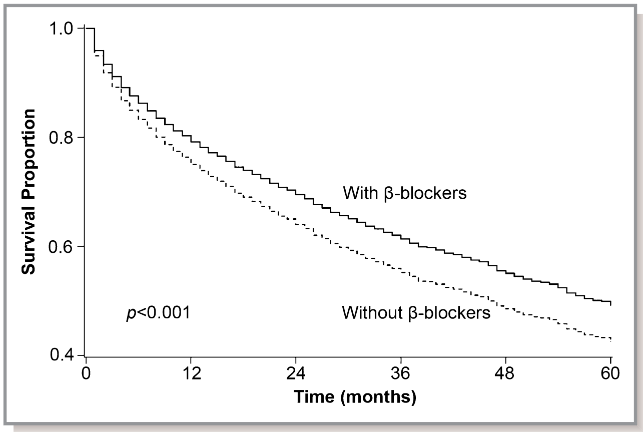

Figure 3. The survival curves of hemodialysis patients with heart failure in a propensity-matched inception cohort after adjustment for the exposure duration of β-blocker therapy and the exposure duration of ACEI or ARB therapy. ACEI indicates angiotensin-converting enzyme inhibitor; ARB, angiotensin type II receptor blocker.

Discussion

The clinical therapeutic guidelines published by the American Heart Association and the European Society of Cardiology recommend carvedilol, bisoprolol, and metoprolol CR/XL for the general treatment of HF, but the therapeutic effects of the

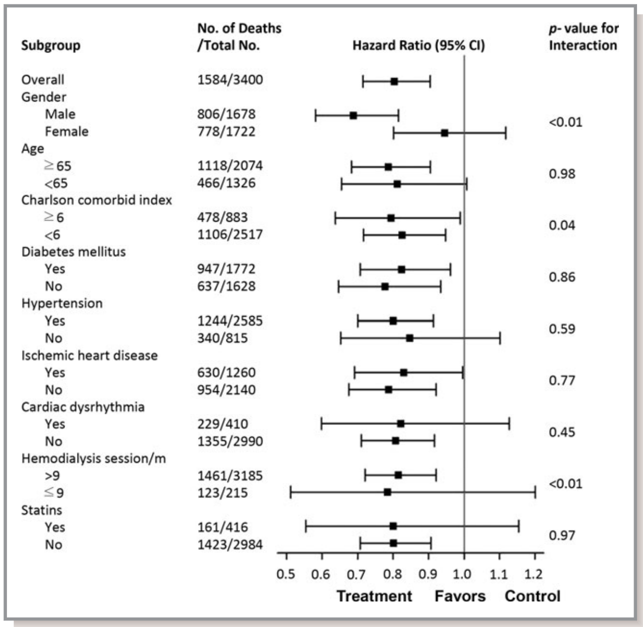

Figure 4. Hazard ratios for all-cause mortality from the final multivariate model and interaction term for selected subgroups.

DOI: 10.1161/JAHA.115.002584

Downloaded from http://jaha.ahajournals.org/ at VA MED CTR BOISE on January 12, 2016

Table 4. Hazard Ratios for All-Cause Mortality by Medication During the Follow-up Period

Subgroups	Total No.	Exposure Time, Person-Years	Death No.	Final Model		
				HR	95% CI	*P* Value
Control group	1700	2893	918			
No β-Blockers, ACEIs, or ARBs	689	958	398	1.74	1.44 to 2.11	<0.001
ACEIs or ARBs	1011	1935	520	1.08	0.90 to 1.31	0.42
Study group	1700	3944	666			
β-Blockers alone	366	597	146	As reference		
β-Blockers plus ACEIs or ARBs	1334	3347	520	0.67	0.55 to 0.81	<0.001

ACEI indicates angiotensin-converting enzyme inhibitor; ARB, angiotensin type II receptor blocker; HR, hazard ratio.

3 β-blockers in HF patients receiving long-term HD have not been rigorously studied.[2,3] In our population-based study, we examine whether therapy with the 3 β-blockers could enhance the survival rate of long-term HD patients with HF.

We demonstrate that therapy based on these 3 β-blockers is associated with a 20% lower risk of all-cause mortality in long-term HD patients with HF (HR 0.80, 95% CI 0.72 to 0.90; $P<0.001$). To reduce any potential bias, we used propensity scores to identify matched cohorts for further analyses, with the results being found to be robust ($P<0.001$) even after adjustment for clinical variables, the exposure duration of β-blocker therapy and the exposure duration of ACEI or ARB therapy (Table 3). We have also found that patients in the study group who received β-blockers plus ACEIs or ARBs had the best survival rates (Table 4).

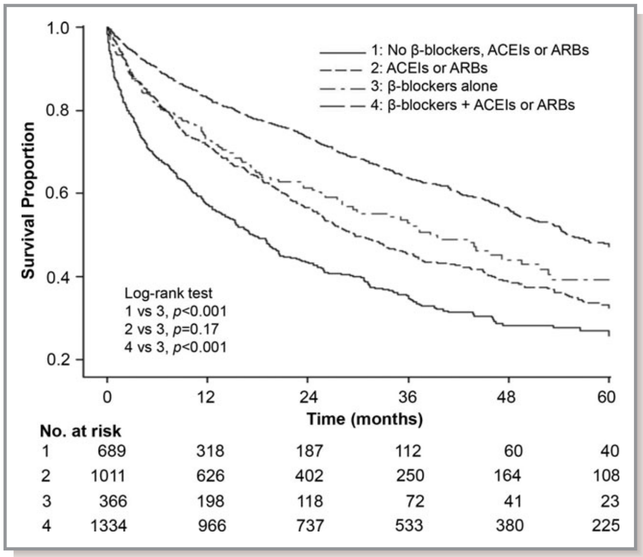

Figure 5. Kaplan–Meier estimates of hemodialysis patients' survival rates in a propensity-matched inception cohort of patients with heart failure, by β-blocker, ACEI, or ARB use in the follow-up period. ACEI indicates angiotensin-converting enzyme inhibitor; ARB, angiotensin type II receptor blocker.

Treatment for HF in the General Population

HF with preserved ejection fraction (HFpEF) may be as common as HF with reduced ejection fraction (HFrEF). Almost 47% of community patients with HF have HFpEF,[27] whereas in Chinese dialysis patients with underlying HF, 55% have evidence of HFpEF.[28] According to epidemiological studies, the identification of HF is validated by using ICD-9 codes[27,29]; however, identifying HFpEF or HFrEF on the basis of ICD-9 codes in this study was impossible because of a lack of detailed echocardiography information.

The benefits of the 3 β-blockers, ACEIs, and ARBs in the general population with HFrEF are indicated based on improved cardiac performance, cardiac remodeling, the number of hospitalizations, and survival.[2,3] A prospective study of 41 791 patients treated with ACEIs or ARBs from the Swedish Heart Failure Registry also revealed an HR of 0.91 (95% CI 0.85 to 0.98) for community patients with HFpEF[30]; thus, ACEIs or ARBs may be used in both HFpEF or HFrEF patients.

β-Blockers are also found to have the benefits of lowering blood pressure, intervening with sympathetic activation, reducing sudden death, and promoting antiremodeling effects.[31–33] However, no prospective or randomized studies have yet been able to convincingly demonstrate that β-blockers reduce morbidity and mortality in a general population with HFpEF.[2,3] The results of a recent meta-analysis of 21 206 patients enrolled in 12 clinical studies demonstrated that β-blocker exposure in patients with HFpEF was associated with a 9% reduction in all-cause mortality (95% CI 0.87 to 0.95)[34]; based on these findings, carvedilol, bisoprolol, and metoprolol CR/XL could be used in patients with HFpEF or HFrEF.

Treatment for HF in HD Patients

Compared with the general population, HD patients have a more activated sympathetic nervous system, a higher prevalence of HF and ischemic heart disease, and a higher risk of sudden

Downloaded from http://jaha.ahajournals.org/ at VA MED CTR BOISE on January 12, 2016

cardiac arrest[35–37]; however, the benefits of ACEIs, ARBs, and β-blockers in HD patients with HFrEF have rarely been evaluated. Only one small prospective study evaluating the use of carvedilol in addition to an ACEI in 114 patients suggested that the treatment had improved survival benefits.[16] Our previous population-based study (involving 4771 patients) showed that ACEIs or ARBs improved the survival benefits of long-term HD patients with HF regardless of whether they were HFpEF or HFrEF (HR 0.80, 95% CI 0.72 to 0.89).[21] In HD patients with HFrEF, the only β-blocker to be studied, and found to have survival benefits, was carvedilol; no studies on either bisoprolol or metoprolol CR/XL treatment have been reported.[16] Similarly, no studies have been carried out to evaluate the benefits of ACEIs, ARBs, and β-blockers in HD patients with HFpEF. We have shown that the 3 β-blockers evaluated in the present study could improve survival in long-term HD patients after time-dependent adjustment for the exposure duration of β-blocker therapy and the exposure duration of ACEI or ARB therapy (HR 0.80, 95% CI 0.72 to 0.90). In summary, the results support our hypothesis and show that β-blockers can have survival benefits on HD patients with HF, as demonstrated by the 20% reduction in all-cause mortality.

β-Blockers as the Initial Therapy for HD Patients With HF

In our real-world clinical analysis, as many as 59.3% of patients received carvedilol to treat their HF, followed by bisoprolol (in 37% of patients). A meta-analysis previously carried out on a general population with HF evaluated the prognostic benefit of β-blockers in patients not receiving ACEIs, with the results revealing that in the absence of an ACEI or ARB at baseline, the risk ratio for β-blockers vis-à-vis placebo was 0.73 (95% CI 0.53 to 1.02), compared with a risk ratio of 0.76 (95% CI 0.71 to 0.83) when these agents were present.[11] The results of the subgroup analysis undertaken in the present study (reported in Table 4 and illustrated in Figure 5) demonstrate that patients who received β-blockers, but not ACEIs or ARBs, exhibited a similar prognostic benefit for all-cause mortality compared with the ACEI or ARB users in the control group. These findings indicate that these 3 β-blockers could be used as the initial therapy for long-term HD patients with HF.

The effects of β-blocker dialyzability on mortality in long-term HD patients were studied recently.[38] Weir et al[38] examined the dialyzability of β-blockers and mortality among older patients receiving HD, although not specifically HF patients, with the results showing that compared with a β-blocker with low dialyzability (carvedilol or bisoprolol), a β-blocker with high dialyzability (metoprolol) was associated with a higher rate of mortality. However, our subgroup analysis results revealed that each of these 3 β-blockers showed significant survival benefits, with no difference in such benefits being discernible between any 2 of the 3 β-blockers (Figure 2B). The possible explanation was that the metoprolol prescribed in the study by Weir et al[38] is a short-acting β-blocker and cannot be extrapolated to the metoprolol CR/XL we studied, which is an extended-release formulation.[7]

Limitations

The implementation of a large, randomized trial would be a significant challenge, essentially because almost 50% of long-term HD patients with new-onset HF are already being treated with β-blockers (Figure 1). For this reason, the matched cohort with time-dependent covariate adjustment used in this study was notable for its national sample size with the use of real-world data to confirm the benefits of β-blockers against new-onset HF in long-term HD patients.

Nevertheless, we caution against any attempt at generalizing our results, essentially because our study has a number of limitations. First, the major drawback of our study is its observational nature, which meant that we could not determine the underlying kidney and heart diseases and randomly assign the patients. Although we have used matched cohorts, there may still be residual confounding factors.

Second, we could not use the assigned ICD-9 codes to identify HF severity (New York Heart Association functional class) or any detailed information on echocardiography (left ventricular end-diastolic and end-systolic volume, ejection fraction, and diastolic function), all of which are associated with mortality. The occurrence of HF was based on the ICD-9 codes registered by the physicians responsible for the treatment of patients. It was verified in general population and our prior study in long-term HD patients with HF.[21,29] However, dyspnea caused by HF or just fluid overload in long-term HD patients made a differential diagnosis difficult if the patient was diagnosed clinically without echocardiography. We also lacked important clinical characteristics and laboratory data on the study population (eg, history of smoking and alcohol consumption, blood pressure, heart rate, electrolytes, and nutrition status). The proportion of patients undergoing echocardiography (coronary angiography) was 66% (10%), and, indeed, those who did not undergo echocardiography may have been clinically diagnosed with HF.

Third, to avoid hypotension during HD, the predialysis dosage of cardiovascular medicine before HD therapy is sometimes reduced in patients with long-term HD; therefore, the β-blockers used in this study may not reflect actual use.

Conclusion

The findings of this study provide clinicians with additional evidence on the therapeutic effects of carvedilol, bisoprolol,

Downloaded from http://jaha.ahajournals.org/ at VA MED CTR BOISE on January 12, 2016

and metoprolol CR/XL in long-term HD patients with HF. In the absence of data from large randomized trials, the findings of this nationwide retrospective cohort study demonstrate that the 3 β-blockers were associated with improved survival in long-term HD patients with HF.

Sources of Funding

This work was supported by grants provided by Wan Fang Hospital, Taipei Medical University, Taipei, Taiwan (grants 100wf-eva-15, 102TMU-WFH-08 and 103TMU-WFH-15).

Disclosures

The authors declare that they have no competing interests.

References

1. Stack AG, Bloembergen WE. A cross-sectional study of the prevalence and clinical correlates of congestive heart failure among incident US dialysis patients. *Am J Kidney Dis*. 2001;38:992–1000.
2. Yancy CW, Jessup M, Bozkurt B, Butler J, Casey DE Jr, Drazner MH, Fonarow GC, Geraci SA, Horwich T, Januzzi JL, Johnson MR, Kasper EK, Levy WC, Masoudi FA, McBride PE, McMurray JJ, Mitchell JE, Peterson PN, Riegel B, Sam F, Stevenson LW, Tang WH, Tsai EJ, Wilkoff BL. 2013 ACCF/AHA guideline for the management of heart failure: executive summary: a report of the American College of Cardiology Foundation/American Heart Association Task Force on Practice Guidelines. *Circulation*. 2013;128:1810–1852.
3. McMurray JJ, Adamopoulos S, Anker SD, Auricchio A, Bohm M, Dickstein K, Falk V, Filippatos G, Fonseca C, Gomez-Sanchez MA, Jaarsma T, Kober L, Lip GY, Maggioni AP, Parkhomenko A, Pieske BM, Popescu BA, Ronnevik PK, Rutten FH, Schwitter J, Seferovic P, Stepinska J, Trindade PT, Voors AA, Zannad F, Zeiher A. ESC guidelines for the diagnosis and treatment of acute and chronic heart failure 2012: the Task Force for the Diagnosis and Treatment of Acute and Chronic Heart Failure 2012 of the European Society of Cardiology. Developed in collaboration with the Heart Failure Association (HFA) of the ESC. *Eur Heart J*. 2012;33:1787–1847.
4. Packer M, Bristow MR, Cohn JN, Colucci WS, Fowler MB, Gilbert EM, Shusterman NH. The effect of carvedilol on morbidity and mortality in patients with chronic heart failure. U.S. Carvedilol Heart Failure Study Group. *N Engl J Med*. 1996;334:1349–1355.
5. Packer M, Coats AJ, Fowler MB, Katus HA, Krum H, Mohacsi P, Rouleau JL, Tendera M, Castaigne A, Roecker EB, Schultz MK, DeMets DL. Effect of carvedilol on survival in severe chronic heart failure. *N Engl J Med*. 2001;344:1651–1658.
6. Packer M, Fowler MB, Roecker EB, Coats AJ, Katus HA, Krum H, Mohacsi P, Rouleau JL, Tendera M, Staiger C, Holcslaw TL, Amann-Zalan I, DeMets DL. Effect of carvedilol on the morbidity of patients with severe chronic heart failure: results of the carvedilol prospective randomized cumulative survival (COPERNICUS) study. *Circulation*. 2002;106:2194–2199.
7. Effect of metoprolol CR/XL in chronic heart failure: metoprolol CR/XL randomised intervention trial in congestive heart failure (MERIT-HF). *Lancet*. 1999;353:2001–2007.
8. Hjalmarson A, Goldstein S, Fagerberg B, Wedel H, Waagstein F, Kjekshus J, Wikstrand J, El Allaf D, Vitovec J, Aldershvile J, Halinen M, Dietz R, Neuhaus KL, Janosi A, Thorgeirsson G, Dunselman PH, Gullestad L, Kuch J, Herlitz J, Rickenbacher P, Ball S, Gottlieb S, Deedwania P. Effects of controlled-release metoprolol on total mortality, hospitalizations, and well-being in patients with heart failure: the metoprolol CR/XL randomized intervention trial in congestive heart failure (MERIT-HF). MERIT-HF Study Group. *JAMA*. 2000;283:1295–1302.
9. The Cardiac Insufficiency Bisoprolol Study II (CIBIS-II): a randomised trial. *Lancet*. 1999;353:9–13.
10. Willenheimer R, van Veldhuisen DJ, Silke B, Erdmann E, Follath F, Krum H, Ponikowski P, Skene A, van de Ven L, Verkenne P, Lechat P. Effect on survival and hospitalization of initiating treatment for chronic heart failure with bisoprolol followed by enalapril, compared with the opposite sequence: results of the randomized Cardiac Insufficiency Bisoprolol Study (CIBIS) III. *Circulation*. 2005;112:2426–2435.
11. Krum H, Haas SJ, Eichhorn E, Ghali J, Gilbert E, Lechat P, Packer M, Roecker E, Verkenne P, Wedel H, Wikstrand J. Prognostic benefit of beta-blockers in patients not receiving ACE-inhibitors. *Eur Heart J*. 2005;26:2154–2158.
12. Wetmore JB, Shireman TI. The ABCs of cardioprotection in dialysis patients: a systematic review. *Am J Kidney Dis*. 2009;53:457–466.
13. Foley RN, Herzog CA, Collins AJ. Blood pressure and long-term mortality in united states hemodialysis patients: USRDS waves 3 and 4 study. *Kidney Int*. 2002;62:1784–1790.
14. Abbott KC, Trespalacios FC, Agodoa LY, Taylor AJ, Bakris GL. Beta-blocker use in long-term dialysis patients: association with hospitalized heart failure and mortality. *Arch Intern Med*. 2004;164:2465–2471.
15. Berger AK, Duval S, Krumholz HM. Aspirin, beta-blocker, and angiotensin-converting enzyme inhibitor therapy in patients with end-stage renal disease and an acute myocardial infarction. *J Am Coll Cardiol*. 2003;42:201–208.
16. Cice G, Ferrara L, D'Andrea A, D'Isa S, Di Benedetto A, Cittadini A, Russo PE, Golino P, Calabro R. Carvedilol increases two-year survivalin dialysis patients with dilated cardiomyopathy: a prospective, placebo-controlled trial. *J Am Coll Cardiol*. 2003;41:1438–1444.
17. Lin YC, Hsu CY, Kao CC, Chen TW, Chen HH, Hsu CC, Wu MS. Incidence and prevalence of ESRD in Taiwan renal registry data system (TWRDS): 2005–2012. *Acta Nephrol*. 2014;28:65–69.
18. Wu VC, Wu CH, Huang TM, Wang CY, Lai CF, Shiao CC, Chang CH, Lin SL, Chen YY, Chen YM, Chu TS, Chiang WC, Wu KD, Tsai PR, Chen L, Ko WJ. Long-term risk of coronary events after AKI. *J Am Soc Nephrol*. 2014;25:595–605.
19. Hsu TW, Liu JS, Hung SC, Kuo KL, Chang YK, Chen YC, Hsu CC, Tarng DC. Renoprotective effect of renin-angiotensin-aldosterone system blockade in patients with predialysis advanced chronic kidney disease, hypertension, and anemia. *JAMA Intern Med*. 2014;174:347–354.
20. Chang YT, Wu JL, Hsu CC, Wang JD, Sung JM. Diabetes and end-stage renal disease synergistically contribute to increased incidence of cardiovascular events: a nationwide follow-up study during 1998–2009. *Diabetes Care*. 2014;37:277–285.
21. Tang CH, Chen TH, Wang CC, Hong CY, Huang KC, Sue YM. Renin-angiotensin system blockade in heart failure patients on long-term haemodialysis in Taiwan. *Eur J Heart Fail*. 2013;15:1194–1202.
22. Rosenbaum PR, Rubin DB. The central role of the propensity score in observational studies for causal effects. *Biometrika*. 1983;70:41–55.
23. Hosmer DW Jr, Lemeshow S, Sturdivant RX. *Applied Logistic Regression*. 3rd ed. Hoboken, New Jersey: John Wiley & Sons; 2013.
24. D'Agostino RB Jr. Propensity score methods for bias reduction in the comparison of a treatment to a non-randomized control group. *Stat Med*. 1998;17:2265–2281.
25. Normand ST, Landrum MB, Guadagnoli E, Ayanian JZ, Ryan TJ, Cleary PD, McNeil BJ. Validating recommendations for coronary angiography following acute myocardial infarction in the elderly: a matched analysis using propensity scores. *J Clin Epidemiol*. 2001;54:387–398.
26. K/DOQI clinical practice guidelines for cardiovascular disease in dialysis patients. *Am J Kidney Dis*. 2005;45:S1–S153.
27. Owan TE, Hodge DO, Herges RM, Jacobsen SJ, Roger VL, Redfield MM. Trends in prevalence and outcome of heart failure with preserved ejection fraction. *N Engl J Med*. 2006;355:251–259.
28. Wang AY, Wang M, Lam CW, Chan IH, Lui SF, Sanderson JE. Heart failure in long-term peritoneal dialysis patients: a 4-year prospective analysis. *Clin J Am Soc Nephrol*. 2011;6:805–812.
29. Svanstrom H, Pasternak B, Hviid A. Association of treatment with losartan vs candesartan and mortality among patients with heart failure. *JAMA*. 2012;307:1506–1512.
30. Lund LH, Benson L, Dahlstrom U, Edner M. Association between use of renin-angiotensin system antagonists and mortality in patients with heart failure and preserved ejection fraction. *JAMA*. 2012;308:2108–2117.
31. Francis GS, Benedict C, Johnstone DE, Kirlin PC, Nicklas J, Liang CS, Kubo SH, Rudin-Toretsky E, Yusuf S. Comparison of neuroendocrine activation in patients with left ventricular dysfunction with and without congestive heart failure. A substudy of the studies of left ventricular dysfunction (SOLVD). *Circulation*. 1990;82:1724–1729.
32. Goldberger JJ. Prevention of sudden cardiac death. *Heart Dis*. 2000;2:305–313.
33. Sharpe N, Doughty RN. Left ventricular remodelling and improved long-term outcomes in chronic heart failure. *Eur Heart J*. 1998;19(suppl B):B36–B39.
34. Liu F, Chen Y, Feng X, Teng Z, Yuan Y, Bin J. Effects of beta-blockers on heart failure with preserved ejection fraction: a meta-analysis. *PLoS One*. 2014;9:e90555.

Downloaded from http://jaha.ahajournals.org/ at VA MED CTR BOISE on January 12, 2016

35. Bakris GL, Hart P, Ritz E. Beta blockers in the management of chronic kidney disease. *Kidney Int*. 2006;70:1905–1913.

36. Alpert MA. Sudden cardiac arrest and sudden cardiac death on dialysis: epidemiology, evaluation, treatment, and prevention. *Hemodial Int*. 2011;15 (suppl 1):S22–S29.

37. Zoccali C, Mallamaci F, Parlongo S, Cutrupi S, Benedetto FA, Tripepi G, Bonanno G, Rapisarda F, Fatuzzo P, Seminara G, Cataliotti A, Stancanelli B, Malatino LS. Plasma norepinephrine predicts survival and incident cardiovascular events in patients with end-stage renal disease. *Circulation*. 2002;105:1354–1359.

38. Weir MA, Dixon SN, Fleet JL, Roberts MA, Hackam DG, Oliver MJ, Suri RS, Quinn RR, Ozair S, Beyea MM, Kitchlu A, Garg AX. Beta-blocker dialyzability and mortality in older patients receiving hemodialysis. *J Am Soc Nephrol*. 2015;26:987–996.

Downloaded from http://jaha.ahajournals.org/ at VA MED CTR BOISE on January 12, 2016

SUPPLEMENTAL MATERIAL

Table S1. The logistic regression model used to calculate the propensity score.

Table S2. Distribution of propensity score among the treat and control groups of the full and matched cohorts.

Table S3. Baseline characteristics of the excluded and included patients in the study group.

Table S4. Incident rate ratios of all-cause mortality for the study group vs the control group.

Table S5. Cox proportional hazard regression on all-cause mortality for the study group vs the control group.

Table S6. Cox proportional hazard regression on all-cause mortality for the study group vs the control group with observation period beginning at different intervals after initial heart failure diagnosis.

Figure S1. The survival curves of hemodialysis patients with heart failure in a propensity-matched inception cohort, 2001-2005.

Figure S2. The survival curves of hemodialysis patients with heart failure in a propensity-matched inception cohort, 2006-2010.

This supplementary material has been provided by the authors to give readers additional information about their work.

**Table S1. The logistic regression model used to calculate the propensity score**					
Parameters	Estimate	Standard Error	Wald Chi-Square	OR (95% CI)	*p*-value
Intercept	-1.3105	0.3754	12.1886		0.0005
Gender					
Male	0.0144	0.0638	0.0511	1.015 (0.895-1.150)	0.8211
Female	Ref				
Age					
35 – 44	Ref				
45 – 54	0.2359	0.1896	1.5486	1.266 (0.873-1.836)	0.2133
55 – 64	-0.1469	0.1810	0.6593	0.863 (0.606-1.231)	0.4168
65 – 74	-0.4757	0.1786	7.0979	0.621 (0.438-0.882)	0.0077
≥75	-0.6810	0.1809	14.1656	0.506 (0.355-0.722)	0.0002
Year at cohort entry					
2001	Ref				
2002	0.1376	0.3207	0.1841	1.148 (0.612-2.152)	0.6679
2003	0.3254	0.3050	1.1385	1.385 (0.762-2.517)	0.2860
2004	0.7462	0.2974	6.2933	2.109 (1.177-3.778)	0.0121
2005	0.7359	0.2936	6.2823	2.087 (1.174-3.711)	0.0122
2006	0.9493	0.2919	10.5774	2.584 (1.458-4.579)	0.0011
2007	0.6303	0.2911	4.6861	1.878 (1.061-3.323)	0.0304
2008	0.7389	0.2904	6.4746	2.094 (1.185-3.699)	0.0109
2009	0.5877	0.2905	4.0934	1.800 (1.019-3.180)	0.0431
2010	0.9116	0.2913	9.7901	2.488 (1.406-4.405)	0.0018
Hospitalization					
0	Ref				
1	-0.0862	0.0991	0.7567	0.917 (0.755-1.114)	0.3844
2	0.1634	0.1076	2.3081	1.178 (0.954-1.454)	0.1287
≥3	0.4694	0.0984	22.7489	1.599 (1.319-1.939)	<0.0001
Charlson comorbidity index					
0-2	Ref				
3-5	0.4386	0.0884	24.6111	1.551 (1.304-1.844)	<0.0001
≥6	0.4268	0.0981	18.9192	1.532 (1.264-1.857)	<0.0001
Comorbidities					
COPD	-0.3196	0.0930	11.8052	0.726 (0.605-0.872)	0.0006
Cirrhosis of liver	-0.5741	0.1622	12.53	0.563 (0.410-0.774)	0.0004
Cerebrovascular disease	-0.3157	0.0809	15.2311	0.729 (0.622-0.855)	<0.0001
Peripheral vascular disease	0.0975	0.1580	0.3813	1.102 (0.809-1.503)	0.5369
Cardiac dysrhythmia	0.0183	0.0998	0.0337	1.018 (0.838-1.238)	0.8543
Ischemic heart disease	0.1345	0.0698	3.7176	1.144 (0.998-1.312)	0.0538
Myocardial infarction	-0.0464	0.1569	0.0875	0.955 (0.702-1.298)	0.7674
Medications at cohort entry					
ACEIs	0.6254	0.1008	38.5292	1.869 (1.534-2.277)	<0.0001
ARBs	0.6735	0.0968	48.3868	1.961 (1.622-2.371)	<0.0001
Calcium channel blockers	0.0317	0.0694	0.2094	1.032 (0.901-1.183)	0.6473
α-blockers	0.0553	0.1428	0.1499	1.057 (0.799-1.398)	0.6987
Hydralazine	-0.0462	0.1860	0.0616	0.955 (0.663-1.375)	0.8040
Nitrates	0.1040	0.0713	2.1275	1.110 (0.965-1.276)	0.1447
Digoxin	0.1423	0.1308	1.1825	1.153 (0.892-1.490)	0.2768
Antiarrhythmics	-0.0512	0.1080	0.2253	0.950 (0.769-1.174)	0.6350

ACEI, angiotensin-converting-enzyme inhibitor; ARB, angiotensin receptor blocker; CI, confidence interval; COPD, chronic obstructive pulmonary disease; OR, odds ratio.

Table S2. Distribution of propensity score among the treat and control groups of the full and matched cohorts

	Full cohort		Matched cohort	
	Study group	Control group	Study group	Control group
No. of observation	2,095	2,344	1,700	1,700
Mean	0.5123618	0.4358370	0.4744712	0.4735048
Standard deviation	0.1379661	0.1276677	0.1196014	0.1197777
Minimum	0.1027599	0.1055752	0.1027599	0.1055752
Lower quartile	0.4125809	0.3421911	0.3909842	0.3901366
Median	0.5103717	0.4342087	0.4768925	0.4722592
Upper quartile	0.6059143	0.5211757	0.5503173	0.5510161
Maximum	0.9071249	0.8648796	0.8582428	0.8648796

**Table S3. Baseline characteristics of the excluded and included patients in the study group**			
Characteristics	Excluded patients (n = 395)	Included patients (n = 1,700)	*p*-value
Gender: male, n (%)	207 (52.4)	844 (49.7)	0.32
Age at cohort entry (y), mean (SD)	58.1 (10.3)	67.3 (11.1)	<0.001
35-44, n (%)	27 (6.8)	54 (3.2)	<0.001
45-54, n (%)	156 (39.5)	210 (12.4)	
55-64, n (%)	124 (31.4)	398 (23.4)	
65-74, n (%)	57 (14.4)	578 (34.0)	
≥75, n (%)	31 (7.9)	460 (27.1)	
Charlson comorbidity index, mean (SD)[a]	3.47 (3.1)	3.68 (2.9)	0.20
No. of hospitalization, mean (SD)[a]	3.3 (2.8)	2.6 (2.5)	<0.001
Duration of dialysis at enrollment (m), mean (SD)	30.6 (23.8)	32.5 (24.3)	0.17
Comorbidities[a] (ICD-9 codes), n (%)			
Ischemic heart disease (411, 413, 414)	184 (46.6)	630 (37.1)	<0.001
Myocardial infarction (410, 412)	14 (3.5)	85 (5.0)	0.22
Cardiac dysrhythmia (426, 427)	51 (12.9)	203 (11.9)	0.59
Cerebrovascular disease (430-438)	35 (8.9)	324 (19.1)	<0.001
Peripheral artery disease (440.2, 443)	26 (6.6)	69 (4.1)	0.03
Hypertension (401-405)	321 (81.3)	1300 (76.5)	0.04
Diabetes mellitus (250)	207 (52.4)	858 (50.5)	0.49
Chronic obstructive pulmonary disease	23 (5.8)	227 (13.4)	<0.001
Cirrhosis of liver (571)	6 (1.5)	64 (3.8)	0.03
Cancer (140-208)	31 (7.6)	174 (10.2)	0.15
Tests or procedures,[b] n (%)			
Echocardiography	283 (71.7)	1126 (66.2)	0.04
Myocardial perfusion scan	72 (18.2)	242 (14.2)	0.05
Coronary angiography	57 (14.4)	166 (9.8)	<0.01
Percutaneous coronary intervention	34 (8.6)	97 (5.7)	0.03
Coronary artery bypass graft	2 (0.5)	12 (0.7)	0.66
24h electrocardiogram	39 (9.9)	180 (10.6)	0.68
Permanent pacemaker implantation	2 (0.5)	13 (0.8)	0.58
Concomitant medication at enrollment[c]			
ACEIs or ARBs	258 (65.3)	494 (29.1)	<0.001
Calcium channel blockers	247 (62.5)	767 (45.1)	<0.001
α-blockers	30 (7.6)	91 (5.4)	0.09
Hydralazine	17 (4.3)	51 (3.0)	0.19
Nitrates	182 (46.1)	602 (35.4)	<0.001
Digoxin	51 (12.9)	102 (6.0)	<0.001
Antiarrhythmics	43 (10.9)	165 (9.7)	0.48
Platelet inhibitors	125 (31.7)	442 (26.0)	0.02
Warfarin	9 (2.3)	37 (2.2)	0.90
Statins	61 (15.4)	214 (12.6)	0.13
Fibrates	20 (5.1)	80 (4.7)	0.76
Oral hypoglycemic drugs	92 (23.3)	364 (21.4)	0.41
Insulins	118 (29.9)	352 (20.7)	<0.001
H2-antagonists or PPIs	106 (26.8)	419 (24.7)	0.37
NSAIDs	191 (48.4)	762 (44.8)	0.20
Benzodiazepines	147 (37.2)	541 (31.8)	0.04

ACEI, angiotensin-converting-enzyme inhibitor; ARB, angiotensin receptor blocker; COPD, chronic obstructive pulmonary disease; NSAID, non-steroidal anti-inflammatory drugs; PPI, proton pump inhibitors.

[a]Within the two-year period prior to the index date.

[b]Within the three-month period before the index date.

Table S4. Incident rate ratios of all-cause mortality for the study group vs the control group

Outcomes	Study group	Control group
2001-2010		
No. of patients	1,700	1,700
No. of all-cause mortality	666	918
Exposure time, patient-year	3,944	2,893
Incidence rate/1000 patient-years	168.9	317.3
Incidence rate ratio (95% CI)	0.53 (0.49-0.58)	1 (reference)
2001-2005		
No. of patients	499	517
No. of all-cause mortality	257	375
Exposure time, patient-year	1,747	1,196
Incidence rate/1000 patient-years	147.1	313.5
Incidence rate ratio (95% CI)	0.47 (0.41-0.53)	1 (reference)
2006-2010		
No. of patients	1,201	1,183
No. of all-cause mortality	409	543
Exposure time, patient-year	2,197	1,697
Incidence rate/1000 patient-years	186.2	320.0
Incidence rate ratio (95% CI)	0.58 (0.53-0.64)	1 (reference)

CI, confidence interval.

Table S5. Cox proportional hazard regression on all-cause mortality for the study group vs the control group

Outcomes	HR	95% CI	*p*-value
2001-2010			
Univariate model	0.56	0.51-0.62	<0.001
Final multivariate model[a]	0.56	0.50-0.62	<0.001
Final model adjusted with time-dependent covariates			
Adjusted for the exposure duration of β-blocker therapy	0.76	0.68-0.85	<0.001
Adjusted for the exposure duration of β-blocker therapy and the exposure duration of ACEI or ARB therapy	0.80	0.72-0.90	<0.001
2001-2005			
Univariate model	0.50	0.43-0.59	<0.001
Final multivariate model[a]	0.51	0.43-0.60	<0.001
Final model adjusted with time-dependent covariates			
Adjusted for the exposure duration of β-blocker therapy	0.80	0.67-0.96	<0.05
Adjusted for the exposure duration of β-blocker therapy and the exposure duration of ACEI or ARB therapy	0.84	0.70-0.98	<0.05
2006-2010			
Univariate model	0.61	0.53-0.69	<0.001
Final multivariate model[a]	0.59	0.52-0.67	<0.001
Final model adjusted with time-dependent covariates			
Adjusted for the exposure duration of β-blocker therapy	0.73	0.63-0.84	<0.001
Adjusted for the exposure duration of β-blocker therapy and the exposure duration of ACEI or ARB therapy	0.77	0.66-0.90	<0.001

ACEI, angiotensin-converting-enzyme inhibitor; ARB, angiotensin receptor blocker; CI, confidence interval; HR, hazard ratio.

[a]The control variables include in the final model demographic variables (sex and age), clinically relevant variables (diabetes, ischemic heart disease, duration of dialysis at enrollment, no. of hospitalization and Charlson comorbidity index), procedures (myocardial perfusion scan, coronary angiography, and percutaneous coronary intervention), and medications at enrollment (fibrates, insulins, H2-antagonists and proton pump inhibitors).

Table S6. Cox proportional hazard regression on all-cause mortality for the study group vs the control group with observation period beginning at different intervals after initial heart failure diagnosis

Models and Adjustments	HR	95% CI	*p*-value
Model 1, beginning at interval of 30 days after HF diagnosis			
Univariate model	0.57	0.51-0.63	<0.001
Final multivariate model[a]	0.55	0.50-0.62	<0.001
Final model adjusted with time-dependent covariates			
Adjusted for the exposure duration of β-blocker therapy	0.81	0.70-0.93	<0.01
Adjusted for the exposure duration of β-blocker therapy and the exposure duration of ACEI or ARB therapy	0.86	0.75-0.97	<0.05
Model 2, beginning at interval of 45 days after HF diagnosis			
Univariate model	0.57	0.52-0.64	<0.001
Final multivariate model[a]	0.56	0.50-0.62	<0.001
Final model adjusted with time-dependent covariates			
Adjusted for the exposure duration of β-blocker therapy	0.83	0.72-0.96	0.01
Adjusted for the exposure duration of β-blocker therapy and the exposure duration of ACEI or ARB therapy	0.87	0.76-0.98	<0.05

ACEI, angiotensin-converting-enzyme inhibitor; ARB, angiotensin receptor blocker; CI, confidence interval; HF, heart failure; HR, hazard ratio.

[a]The control variables include in the final model demographic variables (sex and age), clinically relevant variables (diabetes, ischemic heart disease, duration of dialysis at enrollment, no. of hospitalization and Charlson comorbidity index), procedures (myocardial perfusion scan, coronary angiography, and percutaneous coronary intervention), and medications at enrollment (fibrates, insulins, H2-antagonists and proton pump inhibitors).

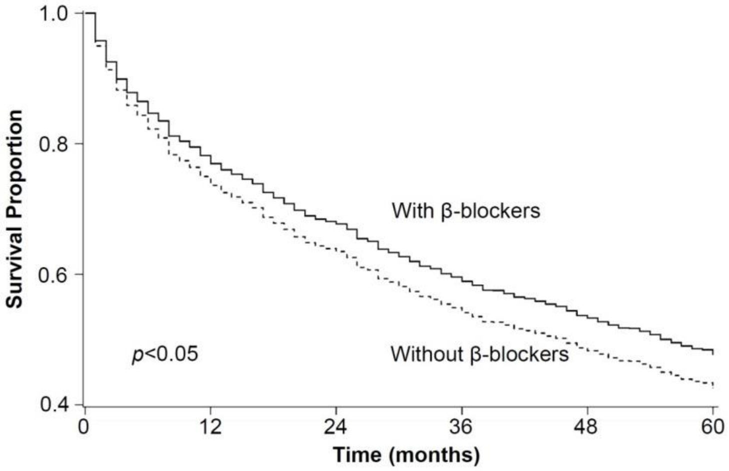

Figure S1. The survival curves of hemodialysis patients with heart failure in a propensity-matched inception cohort after adjusted for the exposure duration of β-blocker therapy and the exposure duration of angiotensin-converting-enzyme inhibitor or angiotensin receptor blocker therapy, 2001-2005.

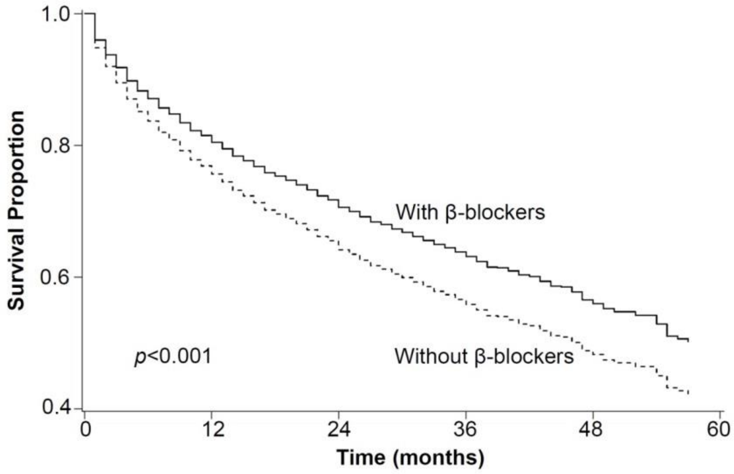

Figure S2. The survival curves of hemodialysis patients with heart failure in a propensity-matched inception cohort after adjusted for the exposure duration of β-blocker therapy and the exposure duration of angiotensin-converting-enzyme inhibitor or angiotensin receptor blocker therapy, 2006-2010.

Journal of the American Heart Association
OPEN ACCESS

Prognostic Benefits of Carvedilol, Bisoprolol, and Metoprolol Controlled Release/Extended Release in Hemodialysis Patients with Heart Failure: A 10−Year Cohort

Chao-Hsiun Tang, Chia-Chen Wang, Tso-Hsiao Chen, Chuang-Ye Hong and Yuh-Mou Sue

J Am Heart Assoc. 2016;5:e002584; originally published January 6, 2016;
doi: 10.1161/JAHA.115.002584
The *Journal of the American Heart Association* is published by the American Heart Association, 7272 Greenville Avenue, Dallas, TX 75231
Online ISSN: 2047-9980

The online version of this article, along with updated information and services, is located on the World Wide Web at:
http://jaha.ahajournals.org/content/5/1/e002584

Data Supplement (unedited) at:
http://jaha.ahajournals.org/content/suppl/2016/01/06/JAHA.115.002584.DC1.html

Subscriptions, Permissions, and Reprints: The *Journal of the American Heart Association* is an online only Open Access publication. Visit the Journal at http://jaha.ahajournals.org for more information.

Downloaded from http://jaha.ahajournals.org/ at VA MED CTR BOISE on January 12, 2016
