# Supplementary material for: Cardio-selective versus non-selective β-blockers for cardiovascular events and mortality in long-term dialysis patients: A systematic review and meta-analysis
Source: PLoS One. 2022 Dec 19;17(12):e0279171. doi: 10.1371/journal.pone.0279171 (PMC9762568; doi:10.1371/journal.pone.0279171)
Supplement: S5 File — (DOCX) [file pone.0279171.s005.docx]

**S5 File. GRADE evidence profile**

| **Outcome** | **Certainty assessment** | | | | | | | **Number of patients** | | **Effect** | **Certainty** | **Importance** |
| --- | --- | --- | --- | --- | --- | --- | --- | --- | --- | --- | --- | --- |
|  | **№ of studies** | **Study design** | **Risk of bias** | **Inconsistency** | **Indirectness** | **Imprecision** | **Other considerations** | **Cardio-selective β-blockers** | **Non-selective β-blockers** | **Pooled HR  (95% CI)** |  |  |
| All caused mortality | 4 | observational studies (Cohort) | serious | not serious | not serious | serious | none | 31221 | 22894 | **0.83** (0.69 to 0.99) | ⨁⨁◯◯ Low | CRITICAL |
| Cardiovascular events | 3 | observational studies  (Cohort) | serious | not serious | not serious | not serious | none | 30306 | 21771 | **0.85** (0.81 to 0.89) | ⨁⨁⨁◯ Moderate | CRITICAL |

**CI:** confidence interval; **HR:** hazard Ratio
